# BAG6 restricts pancreatic cancer progression by suppressing the release of IL33-presenting extracellular vesicles and the activation of mast cells

**DOI:** 10.1038/s41423-024-01195-1

**Published:** 2024-06-28

**Authors:** Bilal Alashkar Alhamwe, Viviane Ponath, Fahd Alhamdan, Bastian Dörsam, Clara Landwehr, Manuel Linder, Kim Pauck, Sarah Miethe, Holger Garn, Florian Finkernagel, Anna Brichkina, Matthias Lauth, Dinesh Kumar Tiwari, Malte Buchholz, Daniel Bachurski, Sabrina Elmshäuser, Andrea Nist, Thorsten Stiewe, Lisa Pogge von Strandmann, Witold Szymański, Vanessa Beutgen, Johannes Graumann, Julia Teply-Szymanski, Corinna Keber, Carsten Denkert, Ralf Jacob, Christian Preußer, Elke Pogge von Strandmann

**Affiliations:** 1 Institute for Tumor Immunology, Philipps-University, 35043 Marburg, Germany; 2 Core Facility Extracellular Vesicles, Philipps-University, 35043 Marburg, Germany; 3 Department of Anesthesiology, Critical Care, and Pain Medicine, Cardiac Anesthesia Division, Boston Children’s Hospital, Boston, USA; 4 Department of Immunology and Anaesthesia, Harvard Medical School, Boston, MA USA; 5 Translational Inflammation Research Division & Core Facility for Single Cell Multiomics, Philipps-University, 35043 Marburg, Germany; 6 Core Facility Bioinformatics, Philipps-University, 35043 Marburg, Germany; 7 Clinic for Gastroenterology, Endocrinology and Metabolism; Center for Tumor and Immune Biology, Philipps-University, 35043 Marburg, Germany; 8 Institute of Systems Immunology, Philipps-University, 35043 Marburg, Germany; 9 Cluster of Excellence on Cellular Stress Responses in Aging-Associated Diseases (CECAD), University of Cologne, Cologne, Germany; 10 Institute of Molecular Oncology and Genomics Core Facility, Member of the German Center for Lung Research (DZL), Philipps-University, 35043 Marburg, Germany; 11 Institute of Lung Health, Justus Liebig University, 35392 Giessen, Germany; 12 Institute of Translational Proteomics & Core Facility Translational Proteomics, Biochemical/Pharmacological Centre, Philipps-University, 35043 Marburg, Germany; 13 Institute of Pathology, Philipps-University Marburg and University Hospital Marburg (UKGM), Marburg, Germany; 14 Department of Cell Biology and Cell Pathology, Philipps-University, 35043 Marburg, Germany

**Keywords:** Pancreatic cancer, EVs, BAG6, Mast cells, Immunotherapy, Tumour immunology

## Abstract

Recent studies reveal a critical role of tumor cell-released extracellular vesicles (EVs) in pancreatic cancer (PC) progression. However, driver genes that direct EV function, the EV-recipient cells, and their cellular response to EV uptake remain to be identified. Therefore, we studied the role of Bcl-2-associated-anthanogene 6 (BAG6), a regulator of EV biogenesis for cancer progression. We used a Cre recombinase/LoxP-based reporter system in combination with single-cell RNA sequencing to monitor in vivo EV uptake and tumor microenvironment (TME) changes in mouse models for pancreatic ductal adenocarcinoma (PDAC) in a *Bag6* pro- or deficient background. In vivo data were validated using mouse and human organoids and patient samples. Our data demonstrated that *Bag6*-deficient subcutaneous and orthotopic PDAC tumors accelerated tumor growth dependent on EV release. Mechanistically, this was attributed to mast cell (MC) activation via EV-associated IL33. Activated MCs promoted tumor cell proliferation and altered the composition of the TME affecting fibroblast polarization and immune cell infiltration. Tumor cell proliferation and fibroblast polarization were mediated via the MC secretome containing high levels of PDGF and CD73. Patients with high *BAG6* gene expression and high protein plasma level have a longer overall survival indicating clinical relevance. The current study revealed a so far unknown tumor-suppressing activity of BAG6 in PDAC. *Bag6*-deficiency allowed the release of EV-associated IL33 which modulate the TME via MC activation promoting aggressive tumor growth. MC depletion using imatinib diminished tumor growth providing a scientific rationale to consider imatinib for patients stratified with low BAG6 expression and high MC infiltration.

**Figure:**
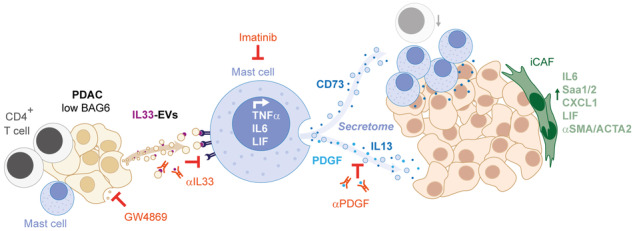
EVs derived from BAG6-deficient pancreatic cancer cells induce MC activation via IL33/Il1rl1. The secretome of activated MCs induces tumor proliferation and changes in the TME, particularly shifting fibroblasts into an inflammatory cancer-associated fibroblast (iCAF) phenotype. Blocking EVs or depleting MCs restricts tumor growth.

EVs derived from BAG6-deficient pancreatic cancer cells induce MC activation via IL33/Il1rl1. The secretome of activated MCs induces tumor proliferation and changes in the TME, particularly shifting fibroblasts into an inflammatory cancer-associated fibroblast (iCAF) phenotype. Blocking EVs or depleting MCs restricts tumor growth.

## Introduction

Pancreatic ductal adenocarcinoma (PDAC) exhibits an extremely poor prognosis with a median survival of 6 months reflecting a high medical need for novel therapy options [1]. A key feature of PDAC progression is the intercellular communication within the tumor tissue which establishes a tumor-promoting environment. Emerging evidence indicates that EVs critically contribute to this cell-cell communication promoting tumor progression and metastasis [2, 3]. EVs, phospholipid bilayer-surrounded nanoparticles, are secreted by any cell into the extracellular environment and loaded with biomolecules including proteins and RNA from the secreting cell. They impact cellular signaling pathways in cancer cells and cells of the TME via interaction with surface receptors/ligands or upon internalization by recipient cells [3]. Cancer progression occurs through several mechanisms and examples in PDAC include EV-mediated liver fibrosis [4], or promotion of metastasis [5–7], e.g., via dysregulation of liver metabolism [8]. Although EV-mediated oncogenic activities were described, the factors directing the formation of tumor-promoting EVs released by PDAC cells, the critical recipient cells, and their downstream effects on the TME are only partially understood.

Our previous work showed that the chaperone BAG6, which is known to regulate intracellular membrane vesicle trafficking [9], is involved in EV biogenesis and protein cargo sorting [10]. This activity was dependent on acetylation of p53 by the BAG6/CBP/p300-acetylase complex, followed by the recruitment of components of the endosomal sorting complexes required for transport (ESCRT) [10]. Here, the recruitment of EV-associated mediators with anti-tumor activity was reported. The protein was identified as a positive regulator of T [11], natural killer (NK) [12], and dendritic cell activity [13]. Acting as a chaperone, BAG6 is moreover involved in cellular processes such as the ubiquitin-proteasome system and protein quality control [14, 15], translational control [16], and mito- and autophagy [17–19]. Almost nothing is known about the role of BAG6 or BAG6-regulated EVs in PDAC progression.

The role of non-malignant host cells in the TME is often complex and context-dependent. This also holds true for mast cells (MCs) in PDAC. They have been associated with tumor promotion, angiogenesis, and immunosuppression in different tumor entities, but were also reported to exhibit anti-tumor activity [20]. Earlier studies reported an essential role of MCs for PDAC tumorigenesis, which was mainly attributed to MC-released IL13 supporting tumor cell proliferation [21, 22], and myc-dependent promotion of angiogenesis [23]. However not much is known about the mechanisms responsible for MC activation in the TME and how or to what extent MCs impact tumor growth and composition.

In this study, we investigated the role of BAG6 in PDAC tumorigenesis. *Bag6*-deficient tumors grew much faster compared to *Bag6*-expressing tumors and were characterized by decreased T cell infiltration and accumulation of inflammatory cancer-associated fibroblasts (iCAFs) indicative of a tumor-promoting TME. This phenotype was rescued upon in vivo inhibition of vesicle release and MCs were identified as EV-recipient cells. The activation of MCs was attributed to engagement of the IL33R with EV-associated IL33. The induced MC secretome promoted tumor cell proliferation and changed the phenotype of tumor-infiltrating cells. Thus, Bag6 deficiency caused EV-dependent and MC-mediated changes in both, tumor cells and the TME fueling tumor progression. BAG6 expression levels correlate with longer survival and inversely correlate with MC infiltration. This suggests that MC depletion is a promising therapeutic approach for patients with low BAG6 expression and high MC cell infiltration.

## Results

### *Bag6* deficiency in tumor cells accelerated tumor growth and induced changes in the TME in PDAC mouse models

Addressing a putative role of the chaperone BAG6 in pancreatic cancer (PC) we measured the BAG6 protein level in plasma samples of PC patients (quantified using Olink Explore 3072 analysis). This analysis revealed that PC patients with low BAG6 expression levels in the serum have significantly shorter overall survival compared to patients with high expression (Fig. S1A). Of note, BAG6 expression declined in human and mouse PDAC cell lines under hypoxic conditions, which are indicative of PDAC [24] (Fig. S1B, C). The potential role of BAG6 in the progression and pathology of PDAC was investigated in the following experiments.

**Fig. 1:**
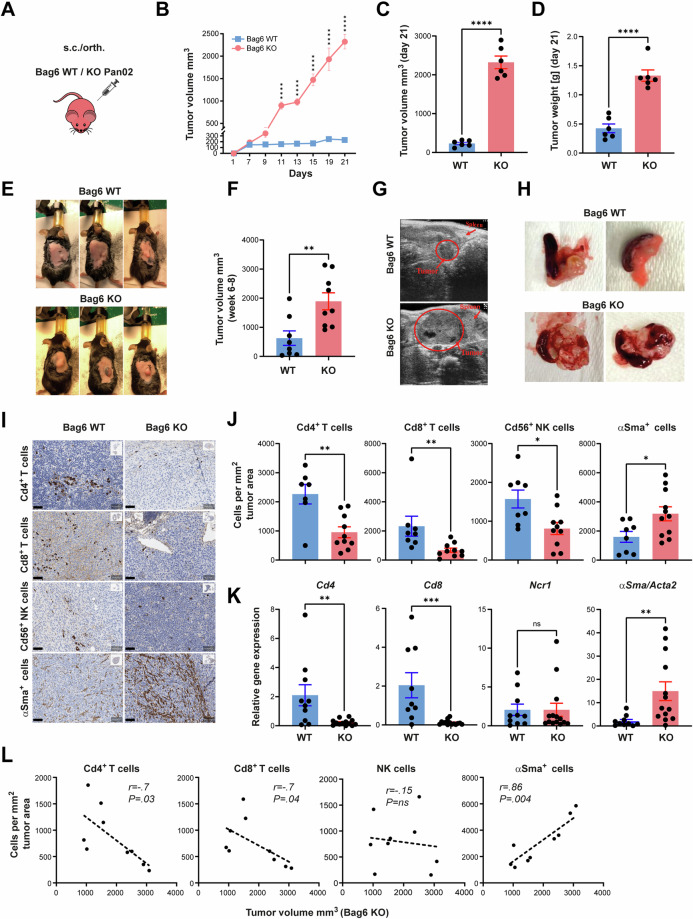
Loss of *Bag6* accelerated pancreatic tumor growth and altered the TME in mouse models. **A** Reporter mice were s.c or orth. transplanted with WT or KO Pan02 cells. **B** Tumor growth of s.c. tumors. **C** Tumor volume at day 21. **D** Tumor weight (mean ± SEM, n = 6). **E** Representative images of s.c. tumors in each group. **F** Tumor volume in orth. model (mean ± SEM, n = 8–9). **G**, **H** Representative images of orth. tumor growth measured via ultrasound. **I** Representative images of immune marker expression in tumor tissue. **J** Immune cells counted per square millimeter of tumor area (mean ± SEM, n = 7–10). **K** Relative gene expression of immune markers normalized to *Rpl32* in tumor tissue from WT and KO tumors (mean ± SEM, n = 8–14). **L** Scatter plot between tumor volume in the KO group and cell types as indicated (n = 9), nonparametric Spearman correlation test. Statistical significance: (**B**) two-way ANOVA followed by Bonferroni corrections for multiple comparisons test; ((**C**), (**D**), (**F**), (**I**), (**J**)) unpaired Mann–Whitney U test; *P < 0.05; **P < 0.01; ***P < 0.001; ****P < 0.0001; n.s. (not significant)

Pan02 Bag6 knock-out (Bag6 KO) and Pan02 wild-type tumor cells (Bag6 WT) were transplanted into immune-competent mice (Fig. 1A). The s.c. tumor growth in the absence of Bag6 was significantly increased according to the volume (Fig. 1B, C) and weight (Fig. 1D). Representative images of s.c. tumors are shown in (Fig. 1E). A similar tumor promotion was observed in the orthotopic model (Fig. 1F) and representative images of the tumors depict the differences in tumor volume (Fig. 1G, H). The Bag6 knockout did not change the growth kinetics in vitro (Fig. S3D).

Immunohistochemistry staining revealed a significant reduction in the infiltration of Cd4^+^ T cells, Cd8^+^ T cells, and Cd56^+^ NK cells in the tumor tissue of the KO group, whereas αSma-expressing fibroblasts were more abundant (Fig. 1I, J). The gene expression of *Cd4* and *Cd8* was also reduced in the tumor tissue, and the expression of *αSma* was increased in KO tumors (Fig. 1K). However, no significant changes were noticed in the expression of the NK cell marker *Ncr1*/*Nkp46* (Fig. 1K).

Furthermore, Spearman correlation analysis validated a negative correlation between the tumor volume and the number of infiltrated Cd4^+^ and Cd8^+^ T cells in KO tumor tissue, whereas the number of infiltrated αSma^+^ cells increased with tumor volume. NK cell infiltration did not correlate with the tumor volume (Fig. 1L). This phenotype was already observed at day 9 (Supplementary Fig. S1D, E), before the onset of significantly accelerated tumor growth suggesting that the altered TME is rather a prerequisite than a result of advanced tumor growth.

These results indicate that Bag6 conferred tumor-suppressing activity in the PDAC models. The absence of Bag6 allowed the establishment of a tumor-promoting TME characterized by reduced infiltration of T cells and enhanced abundance of fibroblasts, known to restrict or support PDAC progression [25], respectively.

### Accelerated tumor growth and remodeling of the TME were mediated by tumor cell-released EVs

**Fig. 2:**
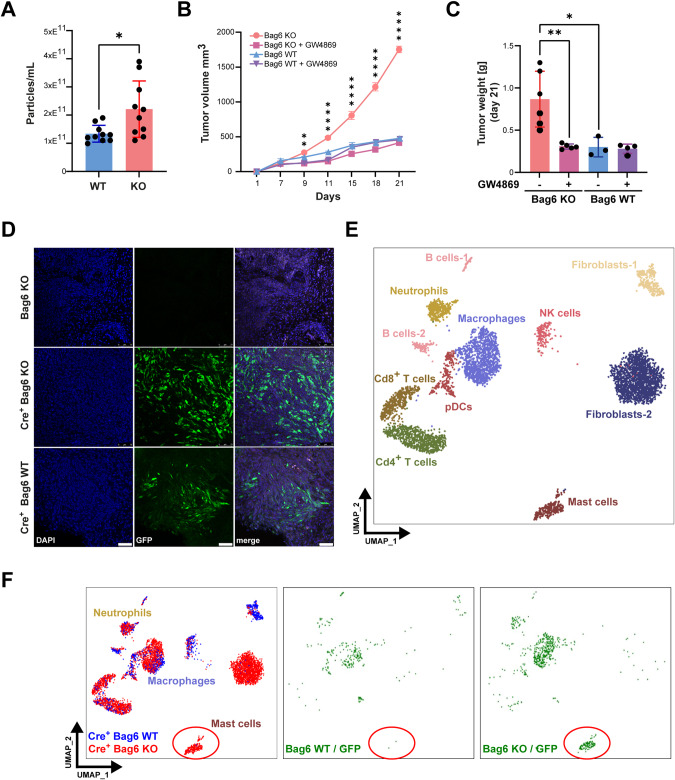
Monitoring of EV uptake in vivo via *Cre-LoxP* and single-cell sequencing. **A** Quantification of particles isolated from the supernatant (SN) of WT/KO Pan02 cells (mean ± SEM, n = 10). **B**, **C** *s.c*. tumor growth and weight of Bag6 WT/KO groups upon treatment with GW4869 (n = 3–6). **D** Confocal images of tumor tissues. GFP^+^ cells correspond to *cre* recombination events and *cre*-negative Bag6 KO tumors were used as negative control. **E** UMAP depiction of cell characterization based on cell markers. **F** UMAP projections of cre^+^ KO (red, left panel) and cre^+^ WT tumors (blue, left panel). Recombination events in WT (middle) and KO (right) are highlighted in green. Statistical significance: (**A**, **C**) unpaired Mann-Whitney U test; (**B**) or two-way ANOVA followed by Bonferroni corrections for multiple comparisons test; *P < .05; **P < 0.01; ***P < 0.001; ****P < 0.0001; n.s. (not significant); UMAP Uniform Manifold Approximation and Projection

Both, Pan02 WT and Bag6 KO cells released EVs, in which the number of EVs from KO cells was slightly higher (Fig. 2A). The inhibition of the in vivo synthesis and release of EVs using GW4869, an EV inhibitor previously described [26] resulted in the reduction of tumor growth and weight (Fig. 2B, C) of Bag6 KO but not WT tumors. This demonstrated the crucial role of EVs in the Bag6 phenotype and tumor progression. The treatment of cells with GW4869 had no toxic effect on tumor cells (Fig. S3D).

EVs from KO and WT cells did not show any differences in size distribution, percentage of tetraspanin positive events (Cd9, Cd63, Cd81), or EV marker expression (Tsg101, Alix, Flotillin-1, Hsp70) (Fig. S2A–D). According to the MISEV guidelines [27], transmission electron microscopy of total EV preparations was performed to validate the expected EV morphology and purity (Fig. S2E).

A more comprehensive proteome characterization using mass spectrometry revealed however distinct protein loading (Fig. S3A, B) with 69 over- and 277 under-represented proteins in KO EVs. WASH complex subunit 4 (Washc4) one of the top hits detected in KO EVs is involved in endosomal sorting and actin cytoskeleton remodeling [28] and other factors contributing to EVs biogenesis, cargo sorting, as well as cancer-related proteins were differently expressed (see Fig. S3A, B).

To investigate the in vivo EV transfer, we employed a *Cre-LoxP* reporter system [29]. We first validated Bag6 expression and *cre*-mRNA expression in tumor cells by Western blotting and PCR, respectively (Fig. S3C, E). The uptake of the *cre recombinase* mRNA delivered via vesicles from transplanted tumor cells is expected to be translated to induce the expression of the GFP reporter gene in the recipient non-malignant host cells (Fig. S4A). To validate this approach, Bag6 KO and WT cells, both releasing EVs containing *cre*-mRNA, and Bag6 KO cells without *cre* were transplanted orthotopically into mice. Immunofluorescence staining of GFP unraveled recombination events in the tumor tissue of animals transplanted with tumor cells releasing *cre*-positive EVs, while absent in mice transplanted with *cre*-negative tumor cells (Fig. 2D). These data suggest that the method is eligible to identify EV-recipient cells within the tumor tissue.

Next, Bag6 KO- and WT-derived orthotopic tumors (n = 2) were resected, digested, and single-cell suspensions subjected to targeted single-cell sequencing (Fig. S4B). Cell clusters were annotated according to their surface markers expression, including B cells, Cd4^+^ T cells, Cd8^+^ T cells, fibroblasts, plasmacytoid dendritic cells (pDCs), macrophages, NK cells, and MCs (Fig. 2E). These cells were further divided into cells derived from KO or WT tumors (Fig. 2F, left panel) and GFP-positive/negative cells (Fig. 2F, middle and right). Overall recombination and vesicle uptake were observed in macrophages, neutrophils, and MCs. Strikingly, the MC cluster in which recombination was observed in almost 100% of the cells was exclusively detectable in KO but absent in WT tumor tissues. This finding suggests that MCs may contribute to the aggressive phenotype of KO tumors. Of note, the phenotype of fibroblasts, which did not show much recombination was also different between KO and WT, portending that fibroblasts are indirectly affected, potentially via MCs. In line, the analysis of the sequencing data using the CellChat Cell-Cell Communication Atlas Explorer [30], unraveled that MCs communicate predominantly with fibroblasts, tumor cells, Cd4^+^ T cells, and macrophages (Fig. S4C).

### Deletion of *Bag6* induced MC activation and infiltration in the TME

**Fig. 3:**
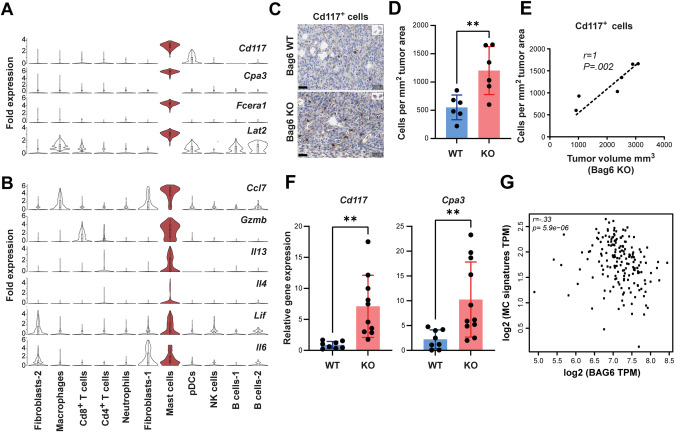
KO tumors were infiltrated by activated MCs. **A**, **B** Violin plots depicting MC markers and cytokines produced by activated MCs in KO tumor tissue. **C** Representative Cd117^+^ cells immunohistochemistry staining in tumor tissue of WT/KO groups. **D** Absolute Cd117^+^ cell counts per square millimeter (mean ± SEM, n = 6). **E** Spearman correlation analysis between tumor size and Cd117^+^ cell expression in the KO tumor tissue (mean ± SEM, n = 6). **F** Relative gene expression of MC markers (*Cd117* and *Cpa3*) in WT/KO tumor tissue normalized to *Rpl32 (*mean ± SEM, n = 8–11). **G** Spearman correlation analysis between MC signature gene expression (*CD117*, *FCERA1*, and *CPA3*) and *BAG6* in PDAC tumors (GEPIA2 analysis ^28^). Statistical significance: (**D**, **F**) unpaired Mann–Whitney U test; *P < 0.05; **P < 0.01

The KO-specific MC cluster was characterized by high expression of MC markers including *c-Kit (Cd117), Cpa3*, *Fcera1*, and *Lat2* (Fig. 3A). Additionally, these MCs showed increased *Gzmb* and cytokine expression (*Cxcl7*, *Il13*, *Il4*, *Lif*, and *Il6*) (Fig. 3B, Fig. S5A), indicative of activated MCs.

The immunohistochemistry of tumor tissue revealed an increase in infiltrated MCs (Cd117^+^ cells) (Fig. 3C, D) which correlated with the volume of the KO tumors (Fig. 3E). In line, RT-qPCR revealed the upregulation of MC-specific genes *Cd117* and *Cpa3* in the tumor tissue (Fig. 3F). Of note, the human MC gene signature (*CD117, FCERA1, CPA3*) and *BAG6* expression correlate inversely in human PDAC tissue (Fig. 3G), in accordance with a putative role for BAG6 in regulating MC infiltration in humans.

**Fig. 4:**
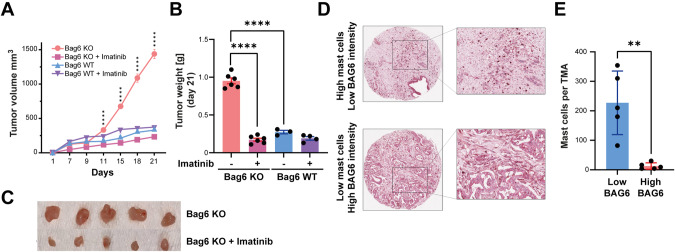
MC depletion reduced tumor growth. **A** Tumor growth curves of Bag6 WT/KO tumors (s.c.) treated twice weekly with imatinib or DMSO control, presented as volume. **B** Tumor weight (mean ± SEM, n = 3–6 mice). **C** Representative images of resected tumors from imatinib- and DMSO-treated animals. **D** Representative MC and BAG6 immunohistochemistry staining of Tissue Microarrays (TMAs). **E** Quantification of CD117^+^ cells in BAG6 high and low samples (mean ± SEM, n = 5). Statistical significance: (**A**) two-way ANOVA followed by Bonferroni corrections for multiple comparisons test; (**B**, **E**) unpaired Mann–Whitney U test; *P < 0.05; **P < 0.01; ***P < 0.001; ****P < 0.0001; n.s. (not significant)

To directly test the contribution of MCs to KO tumor progression we applied imatinib, a tyrosine kinase inhibitor that targets Cd117 and depletes MCs [31]. We observed a significant reduction in KO tumor growth and weight after imatinib treatment (Fig. 4A–C) demonstrating the critical role of MCs in tumor progression. The tumor growth and weight of WT tumors remained unaffected after imatinib treatment (Fig. 4A, B) and imatinib had no or minimal direct toxic or inhibitory effects on tumor cells in vitro (Fig. S5B).

To address MC infiltration in human PDAC, we used immunohistochemistry to stain both MCs (CD117) and BAG6 in TMAs (n = 69). We assessed MC infiltration and then selected 5 patients with the highest and 5 patients with the lowest MC infiltration prior to classification into high or low BAG6 expression. Representative images revealed that high MC infiltration corresponded to low BAG6 protein intensity, and vice versa (Fig. 4D, E; Fig. S5C) suggesting that patients with high MC infiltration and low BAG6 expression might benefit from imatinib therapy.

### *Bag6*-deficient tumor cells release Il33-presenting EVs which induced MC activation

**Fig. 5:**
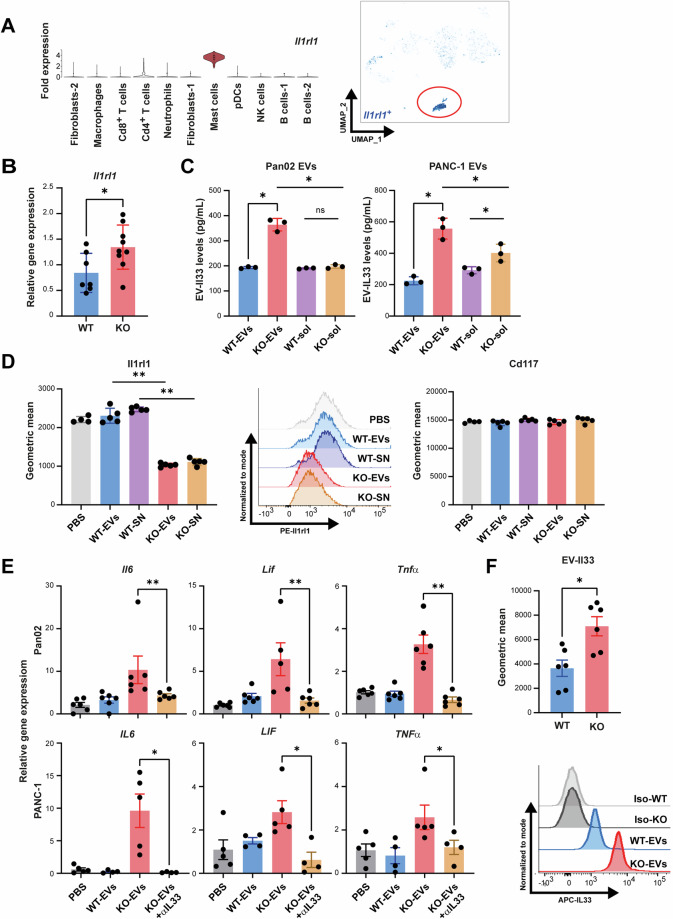
EV-associated IL33 induced MC activation. **A** Violin plots and UMAP depiction of Il1rl1 (Il33 receptor) expression in KO tumors. Expression of Il1rl1 was low on Treg cells and similar between KO and WT (Fig. S5D). Surface expression of Il1rl1 measured using flow cytometry was detectable on MC/9 cells but absent on WT and KO Pan02 cells (Fig. S5E). **B** RT-qPCR of *Il1rl1* gene expression in tumor tissue normalized to *Rpl32* (mean ± SEM, n = 7–9). **C** ELISA to detect mouse and human IL33 in EVs and EV purified soluble fractions (-sol) from WT and KO PDAC cells (mean ± SEM, n = 3). **D** Murine MCs pre-treated with EVs or crude supernatant (SN) from KO and WT Pan02 cells were analyzed for Ilrl1(IL33 receptor) expression. Cd117 expression was used as a control (mean ± SEM, n = 4–5). **E** Cytokine gene expression analysis of mouse and human MCs stimulated with KO- or WT-EVs isolated from Pan02 and PANC-1 cells, respectively. Data were normalized to *Rpl32* (mean ± SEM, n = 4–6, 2 independent experiments). **F** Microbead assay to measure Il33 expression on WT-/KO-EVs from Pan02 cells (mean ± SEM, n = 6). Statistical significance: (**A**–**F**) unpaired Mann–Whitney U test t-test; *P < 0.05; **P < 0.01

Single-cell sequencing of KO tumors revealed a notable and MC-specific expression of *Il1rl1* (ST2/Il33 receptor) (Fig. 5A), which was confirmed by higher overall expression of *Il1rl1* in KO tumor tissues (Fig. 5B). Thus, we tested whether MC activation was driven via Il33/Il1rl1 interaction. First, EVs were collected from mouse Pan02 and human PANC-1 cells with either WT or KO genetic background to perform an IL33/Il33-specific ELISA. The amount of EV-associated IL33/Il33 protein was elevated in KO-EVs (Fig. 5C). IL33 was not increased in the soluble fraction of the EVs purification from Pan02 WT or KO cells (WT-sol, KO-sol), whereas a moderate increase was observed in the corresponding sample of PANC-1 cells. Thus, the contribution of soluble IL33 cannot be excluded. Next, to explore the binding interaction between Il33 and Il1rl1, MCs were incubated with EVs or crude supernatant (SN) from WT or KO Pan02 cells, and Il1rl1 receptor expression on the MCs was assessed using flow cytometry (Fig. 5D). A significant reduction in Il1rl1 level upon incubation with KO (EVs and SN) was observed, indicating blocking of the receptor. The Il1rl1 signal was not changed when incubating MCs with WT samples (EVs and SN). The quenching was specific for I1rl1 since the detection of Cd117 remained unchanged upon pre-incubation with EVs.

The stimulation of mouse or human MCs with KO-EVs, but not with WT-EVs induced the expression of cytokines including *Il6*, *Lif*, and *Tnfα* (Fig. 5E), cytokines that are inducible via the Il33 signaling cascade [32]. Of note, inhibition of this signaling pathway using anti-IL33 blocking antibodies diminished the expression of these cytokines (Fig. 5E). In line, CRISPR/Cas9-mediated knock-out of *Il33* in Bag6 KO cells, or MC pre-treatment with a blocking anti-Il1rl1/ST2 receptor, abrogated the EV-mediated MC activation (Fig. S6A, B).

To validate vesicle-associated Il33 expression directly, Il33 was detected using flow cytometry on beads-coupled EVs. The expression level of Il33 was significantly higher on EVs from KO cells (Fig. 5F), which is in line with the ELISA data (Fig. 5C). These data demonstrate that IL33/Il1rl1 engagement drives MC activation.

### *Bag6* regulates Il33 protein level via degradation and inhibition of release

**Fig. 6:**
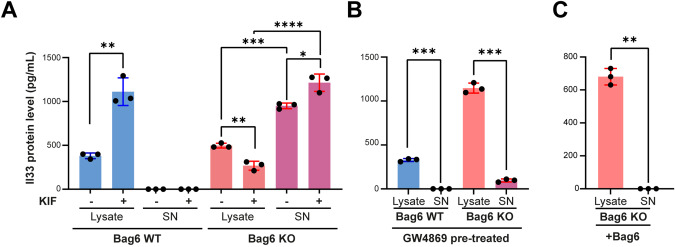
Bag6-mediated regulation of Il33 protein level and release. **A**, **B** ELISA to detect IL33 in cell lysate or supernatant (SN) of WT/KO Pan02 cells pretreated with or without Kifunensine (KIF) (150 µM) in (**A**) or with GW4869 (20 µM) in (**B**). Treatment was performed for 24 h followed by replacement of medium and 24 h incubation (n = 3 ± SEM). **C** ELISA to detect IL33 in cell lysate and SN after rescue of the Bag6 expression in Bag6 KO Pan02 cells (n = 3 ± SEM). Statistical significance: (**A**–**C**) unpaired t-test; *P < 0.05; **P < 0.01; ***P < 0.001; ****P < 0.0001

Given the role of Bag6 in protein biogenesis and endoplasmic reticulum-associated degradation (ERAD) [33] we tested whether kifunensine (KIF), an inhibitor of BAG6-mediated ERAD [34], affected IL33 protein level. Treatment of cells with KIF resulted in an accumulation of Il33 in the lysates of Pan02 WT cells, an effect that was not seen in Pan02 KO cells, indicating that Il33 is degraded via the proteasomal pathway in a Bag6-dependent manner (i). Notably, increased cellular Il33 protein levels in WT cells did not result in Il33 release, which was only detectable in KO cells suggesting that Bag6 counteracts Il33 secretion (Fig. 6A). Secretion of Il33 in association with exosomes was recently observed in the context of chronic airway disease and shown to be mediated via the neutral sphingomyelinase 2 pathway (nSmase) [35], an enzyme also involved in the Bag6-mediated loading and release of EVs via the endosomal sorting complexes required for transport (ESCRT) pathway [10]. We therefore investigated whether Il33 release is mediated via the nSmase pathway from Pan02 cells. Bag6 WT/KO cells were treated with the nSmase inhibitor GW4869. Remarkably, this led to Il33 accumulation in KO cells, while WT cells remained unaffected (Fig. 6B), indicating that EV-associated Il33 release in KO cells is mediated through the nSmase pathway, which is inactive in WT cells. Finally, we rescued the expression of Bag6 in KO cells, which reproduced the WT Il33 phenotype (Fig. 6C). In summary, these data show that Bag6 promoted Il33 degradation (i) and inhibited the nSmase-dependent release of Il33 (ii).

### The secretome of EV-activated MCs promoted tumor growth

**Fig. 7:**
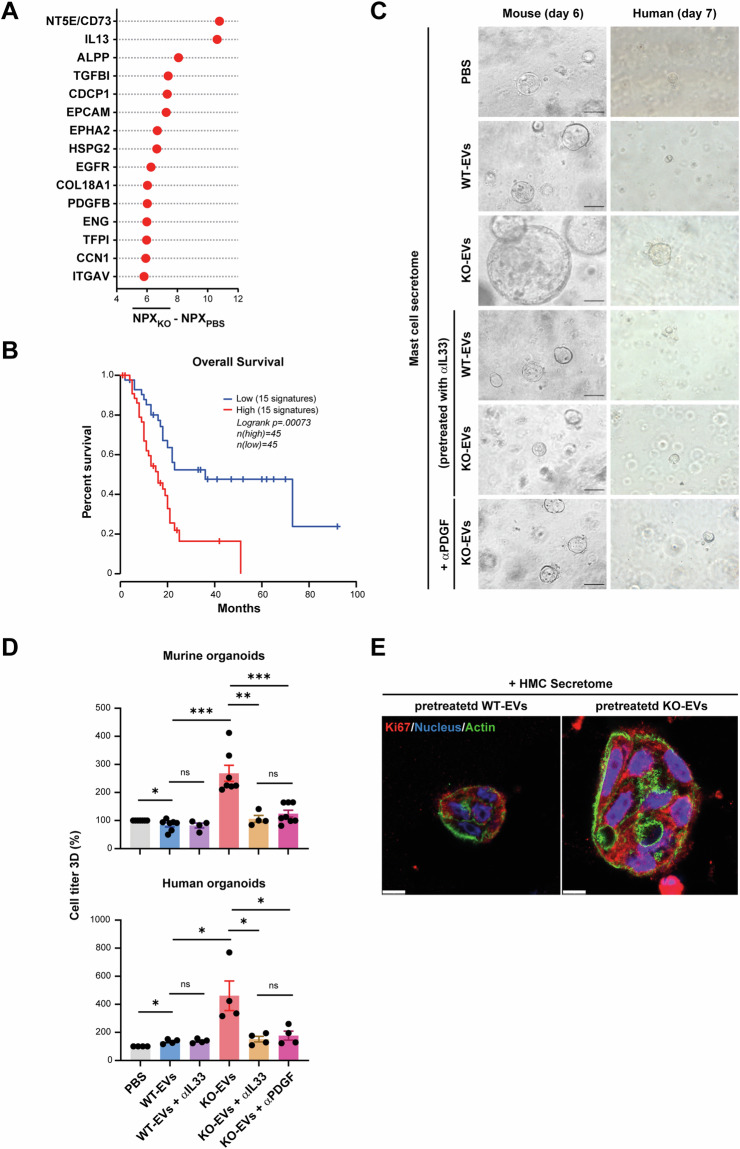
The secretome of MCs activated with KO-EVs promoted tumor growth. **A** The top 15 upregulated proteins in the secretome of human MCs pre-treated with PANC-1 KO-EVs (n = 2) are depicted. Normalized protein expression values (NPX) were averaged and the effect size calculated as compared to a PBS control is indicated (full protein list in Supplementary Table S1 and Fig. S6D for the global distribution of the effect size for the 15 upregulated proteins). **B** Kaplan Meier analysis of the top 15 upregulated proteins correlated with survival (TCGA data via GEPIA2). **C** Representative images of KPC mouse and PDAC human organoids after treatment with MC secretome from mouse/human MCs that were pre-treated with WT- or KO-EVs from Pan02 or PANC1, respectively as well αIL33 and αPDGF as indicated. **D** Quantification of organoid sizes determined via cell titer (mean ± SEM, n = 4–6). **E** The organoids were stained with anti-Ki67 antibodies (red), with Hoechst 33342 (nuclei, blue) and Alexa 546 phalloidin (actin, green) to visualize cellular structures. Scale bars: 10 µm. Statistical significance: (**D**) unpaired Mann–Whitney U test; *P < 0.05; **P < 0.01; ***P < 0.001

Next, the secretome of human MCs exposed to KO-EVs derived from PANC-1 cells was collected and released proteins were quantified using antibody-based proximity extension assay (Fig. S6C). Of note, the 15 top-upregulated proteins (Fig. 7A) reflect a spectrum of regulatory mediators, such as CD73, IL13, and TGFβ, which influence immune cell and fibroblast functions (for global distribution see Fig. S6D, full list Supplementary Table S1). Furthermore, we observed high levels of factors associated with tumor cell proliferation (CDCP1, EpCAM, EGFR, and PDGFB). This MC secretome signature of the up-regulated factors is associated with shorter survival in PDAC patients (TCGA expression database, Fig. 7B) further indicating the critical role of the MC secretome in promoting tumor progression in PDAC patients.

Finally, the impact of the MC secretome on tumor cell proliferation was assessed (Fig. 7C, D). Initially, human and mouse MCs were stimulated with either WT- or KO-EVs, in the presence or absence of anti-IL33 antibodies (Fig. S6E). Subsequently, the obtained secretomes were co-cultured with human or mouse PDAC organoids. An increase in size, number, and cell titer of organoids when cultured with the secretome from MCs stimulated with KO-EVs was observed in both models (Fig. 7C, D). This effect was absent when human/mouse MCs were pretreated with anti-IL33 antibodies. Notably, we observed no changes in the organoids when cultured with secretomes obtained from MCs stimulated with WT-EVs or PBS, regardless of the presence or absence of anti-IL33.

Due to the important role of PDGF in promoting tumor growth [36] and its high abundance in the MC secretome (Fig. 7A), the secretome of human/mouse MCs treated with KO-EVs was incubated with anti-PDGF antibody during organoid stimulation. Anti-PDGF antibodies effectively blocked the growth of organoids and significantly reduced the cell titer (Fig. 7C, D). Whole-mount staining of organoids allowed the visualization of single cells and confirmed the increased proliferation after KO-secretome pretreatment (Fig. 7E).

Further analysis revealed an increase in gene expression of cell cycle-associated factors in the KO-EV group and the corresponding pathways, which is in line with the induction of organoid proliferation (Fig. S6F, G). These findings suggest the crucial role of EV-associated IL33 as an upstream stimulator for MCs in the absence of BAG6, and PDGF as a downstream stimulator promoting tumor growth.

### The activated MC secretome-induced iCAF polarization

**Fig. 8:**
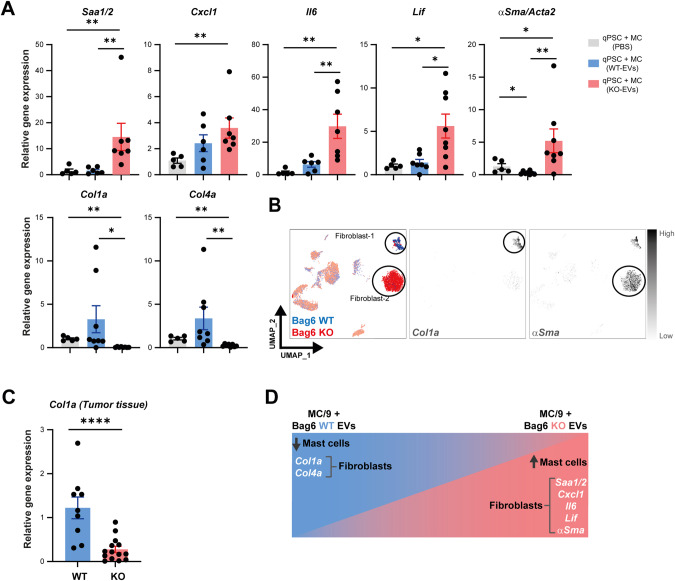
The secretome from activated MCs induced an iCAF phenotype. **A** mPSCs were treated with MC secretome of MCs pre-treated with WT- or KO-EVs. Gene expression of iCAF markers and mCAF markers was determined by RT-qPCrR. (mean ± SEM, n = 6). **B** UMAP projection of fibroblast markers *Col1a* and *aSMA* in WT/KO tumors. **C** *Col1a* expression in KO and WT tumors determined by RT-qPCR. Data were normalized to *Rpl32* (mean ± SEM, n = 9–14). **D** Graphical summary of mPSC/fibroblast phenotypes in vivo or after MC secretome treatment. Statistical significance: unpaired Mann–Whitney U test; *P < 0.05; **P < 0.01, ***P < 0.001, ****P < 0.0001

Given the differences in the phenotype and infiltration of fibroblasts in the KO/WT tumor tissues (Figs. 1I–K and 2F (left UMAP), we investigated the impact of the MC secretome on mouse pancreatic stellate cells (PSCs) in vitro. PSCs were seeded in Matrigel drops and maintained in starvation medium to induce quiescence (q)PSCs. Culturing qPSC with the secretome from MCs stimulated with KO-EVs resulted in an iCAF phenotype, characterized by the expression of *Saa1/2*, *Cxcl1*, *Il6*, *Lif*, and *αSma* genes (Fig. 8A, upper panel). In contrast, qPSC cultured with the secretome from MCs stimulated with WT-EVs exhibited a myofibroblast-like cancer-associated fibroblast (myCAF) phenotype (upregulation of *Col1a* and *Col4a*) (Fig. 8A, lower panel). In line with these findings, single-cell sequencing data and gene expression analysis of tumor tissues from WT and KO groups displayed an upregulation of *Col1a* in the WT and *αSma* in the KO group, respectively (Fig. 8B, C) corresponding to the fibroblasts-1 and -2 clusters (Fig. 2E, F). The genes are differentially regulated in vivo and in vitro in fibroblasts upon MC secretome treatment and are summarized in (Fig. 8D).

In summary, this study unravels that the loss of BAG6 provoked the release of EV-associated IL33 which activated MCs. The secretome of these activated cells (i) directly stimulated tumor cell proliferation and (ii) exerted an impact on fibroblasts supporting a tumor-promoting environment. Depletion of MCs using imatinib restricted tumor development in a *Bag6*-deficient mouse model, indicating that patients with low BAG6 expression and high MC infiltration may benefit from imatinib therapy (see graphical abstract for summary).

## Discussion

Although cancer-derived EVs are reported to play a key role in PC progression, their primary target cells and induced cellular responses in vivo are largely unknown. In this study, we investigated the role of the chaperone BAG6 for the activity of EVs in PDAC. We identified recipient cells of tumor-released EVs in vivo using a reporter approach based on vesicle-mediated recombination. Simultaneously we analyzed the cellular response of these non-malignant cells upon vesicle uptake using single-cell sequencing. This revealed a hitherto unreported tumor-suppressive role of BAG6 in PDAC which was attributed to its role in the formation and release of EVs. Deletion of BAG6 strongly accelerated tumor growth in an MC-dependent manner and this effect was rescued upon in vivo inhibition of the EV release.

This study is the first that highlights the major role of PDAC-EVs in MC activation as a key element in orchestrating the TME and promoting tumor cell growth. This is exclusively observed in the context of low or absent BAG6 and dependent on IL33-engagement of Il1rl1 on MCs.

Few studies addressed the role of MCs in PDAC [21–23, 37–39] and reported their impact on angiogenesis and tumor cell migration predominantly via the release of Il13, which was among the top up-regulated factors released by MCs upon IL33-dependent stimulation (Fig. 7A). Reflecting the scenario that the combination of secretome mediators rather than a single factor released by MCs promotes tumor progression, we observed that the MC secretome signature correlated with the survival of patients. Among these factors are growth factors including PDGF, shown to be responsible for the accelerated tumor cell proliferation of organoids (Fig. 7C, D). The top-upregulated mediator was CD73, an enzyme involved in the adenosinergic pathway that plays a critical role in reshaping an immunosuppressive TME [40]. This factor may thus contribute to the ability of activated MCs to remodel the TME (Fig. 1), and targeting CD73 is considered as a therapeutic strategy in PDAC [41].

The activation of MCs and the tumor-promoting secretome was mainly induced via IL33 receptor engagement in MCs (Fig. 7C, D) both in murine and human organoids. A secretion of IL33 associated with exosomes was recently observed in the context of chronic airway disease and shown to be mediated via the ceramide biosynthetic enzyme neutral sphingomyelinase 2 pathway [35], also involved in BAG6-mediated vesicle biogenesis [42].

IL33 is an alarmin belonging to the IL1 cytokine family, which is released upon tissue damage in the context of cell death. Proteases from target cells such as MCs can cleave IL33 extracellularly to allow binding and activation via I1rl1 and IL33 is critically involved in innate and adaptive immunity in allergic and non-allergic inflammation [43]. Recent studies address the role of IL33 in the context of PC and both, tumor-promoting and tumor-suppressing activities are discussed. IL33 exhibited tumor restriction in PDAC models by stimulating or restoring anti-tumor activity of CD8^+^ T and NK cells and was shown to act as an immunoadjuvant to enhance antigen-specific tumor immunity [44–46]. In line, blocking tumor-associated macrophages which decreases TNFα and elevates IL33 resulted in increased DC activity and infiltration of cytotoxic T cells, with anti-tumor activity [47]. In contrast, another study identified Il33 as a main target of the gene regulatory programs that allow the “acinar-to-neoplasia” switch and the initiation of PC in the context of *KRAS* mutations [48, 49]. Moreover, exogenous Il33 mimicked this effect and was associated with an accelerated appearance of pancreatic neoplasia [48, 49]. High Il33 expression in tumor cells recruits and activates T_H_2 and ILC2 cells which secrete pro-tumorigenic cytokines such as Il4, Il5, and Il13 [50]. Interestingly, the intratumoral fungal mycobiome was identified as the inducer of the increased Il33 in tumor cells [50]. The context-dependent discrepancies of the IL33 effect on tumor growth may depend on specific recruitments of non-malignant immune cells to the tumor varying in different cancer subtypes. Our study provides evidence that specifically the presence of IL33-activated MCs fuels tumor growth and confers pro-tumorigenic activity, suggesting that corresponding patients may benefit from MC depletion. Although single agent treatment with imatinib in a small number of PC patients was not associated with significant control of cancer progression [51, 52] we provide evidence that a targeted therapy of PDAC patients stratified for high MC infiltration is promising.

## Materials and methods

The sources and identifiers of all key reagents and resources are listed in Supplementary Tables 1–6 or in the Supplementary Materials. Extended details on EVs characterization, isolation, EV proteomics, EV-IL33 ELISA, immunoblotting, immunohistochemistry, and immunofluorescence are listed in the Supplementary Materials and Methods.

### Cell lines, plasmids, and culturing media

Murine pancreatic adenocarcinoma cell line Pan02 (RRID: CVCL_D627), human PANC-1 (CRL-2553™), PaTu8988T (ACC 162), and the murine pancreatic stellate cells (mPSCs) (kindly provided by A. Neesse [53]) were cultured with Dulbecco’s Modified Eagle Medium (DMEM) (Gibco, NY, USA) supplemented with 10% fetal bovine serum (FBS) (Gibco).

Human MC line HMC-1 (1 SCC067) was grown in Iscove’s Modified Dulbecco’s Medium (IMDM) (Gibco), supplemented with 10% FBS and the murine MC line MC/9 (CRL-3616™) was grown in high glucose DMEM with 2 mM l-glutamine, 0.05 mM 2-mercaptoethanol, 10% T-Cell Culture Supplement (Corning™ 354115) and 10% FBS. All cells were kept at 37 °C in a humidified atmosphere containing 5% CO_2_ and tested for mycoplasma contamination at least bi-monthly.

The *BAG6*/*Bag6* knockout (KO) was performed based on Zhang Lab’s reagents and protocols [54] (guide RNA sequences in Supplementary Table S2). The pcDNA3.1 CMV-CFP-Ubc-Cre-zipcode-zeo plasmid was transfected for stable Cre expression (kindly provided by Jacco van Rheenen [29]). Three KO clones were pooled for further experiments. Bag6 protein expression and *Cre* expression were measured using Western blot and PCR, respectively (Fig. S2F, H). Il33 knockout (KO) was performed in Bag6 WT/KO Pan02 cells using the Il33 CRISPR/Cas9 KO Plasmid (m2) according to the manufacturer’s instructions (Santa Cruz Biotechnology, USA; Cat. No. sc-429508-KO-2). Re-expression of BAG6 was achieved using a pcDNA3.1 expression construct previously described [42].

To investigate Il33 protein level and release in Bag6 WT/KO Pan02 cells, cells were treated with GW4869 (20 µM) and KIF (150 µM) as indicated in the figure legends.

### Animals and in vivo experiments

All mouse experiments were performed according to the German Animal Welfare Law (TierSchG) and the ARRIVE guidelines. Experiments were approved by the Regierungspräsidium Gießen (G16/2016, G11/2018, and G14/2024) based on recommendations of their animal welfare committee. Male/female at least 10 weeks old mice were randomized in cages before starting any procedure. Wild-type *C57BL/6* mice were purchased from Charles River (Wilmington, Massachusetts, USA) and reporter mice *B6.129(Cg)-Gt(ROSA)26Sortm*^*4(ACTB-tdTomato,-EGFP)Luo/J*^ were purchased from JAX Laboratories (Bar Harbor, Maine, USA). For the orthotopic (orth.) model, mice were anesthetized, and a ca. 1 cm incision was made into the side of the abdominal cavity. The tail of the pancreas was taken out and injected with 20 µL of cold Matrigel (Corning, 356230) containing (1 × 10^6^) Pan02 cells. We used a 27 G needle for injection, then the incision was closed, and tumor growth was followed by ultrasound. In the subcutaneous (s.c.) model, mice were anesthetized and inoculated with (1 × 10^6^) Pan02 cells using a 29G needle. For GW4869 or imatinib treatment, a subgroup of mice transplanted with Bag6 WT/KO Pan02 cells received intraperitoneal (i.p.) injections of GW4869 (Selleckchem, S7609, Pittsburgh, USA) (50 mg/kg BW) or imatinib (Selleckchem, S2475) (60 mg/kg BW), dissolved in 2% DMSO + 30% PEG 300 + 2% Tween 80 and diluted to working concentrations with 2% DMSO (25 µL for GW4869 and 50 µL for imatinib) before injection, twice a week, to inhibit EV production or to deplete MCs, respectively. The control groups received 2% DMSO accordingly. Tumor sizes were measured using calipers at the indicated time points. The tumor volume (V) in each model was calculated using the formula V = (L × W^2^)/2, where L is the length and W is the width dimension.

### Human samples

All human sample analyses were approved by the local ethics committee of Philipps University Marburg (Reference Nr: 76/17 and amendments). Plasma was provided by the *Comprehensive Biobank Marburg* (CBBMR) after informed written consent by the patients (patient characteristics see Supplementary Table S3).

Human pancreatic tissue microarrays (TMAs) were embedded in paraffin donor blocks and placed into pre-punched holes on recipient paraffin blocks using the AlphaMetrix manual tissue arrayer (AlphaMetrix Biotech, Rödermark, Germany). Blocks were sealed and incubated first at 56 °C for 10 min and then at 4 °C for 30 min. TMA blocks (n = 69) were cut into 2 μm sections for immunohistochemical staining. Human organoid samples (passage 14) were derived from a PDAC patient (60 years, male).

### Tumor preparation for single-cell sequencing

The tumor was carefully resected, washed, and cleaned from fat, necrotic tissue, and blood vessels, then cut into small pieces of 2–4 mm^3^ and transferred to gentleMACS C Tube with 5 ml RPMI medium supplemented with 10% FBS and 0.5 mg/mL Collagenase D (Cat. 11088866001 Merck, Germany) + 10 µg/mL of DNase I (Merck, Cat. 11284932001). The cell suspension preparation was performed using the gentleMACS Dissociator after two steps of digestion. The cell suspension passed through 30 μm strainers twice. The cells were then centrifuged (300 × *g*, 4 °C for 10 min) and resuspended in 1 ml of MACS buffer before being counted for single-cell sequencing.

### Targeted single-cell sequencing

Employing the BD Rhapsody™ Single-Cell Analysis System (Becton Dickinson, San Diego, California, USA) and BD protocol (Doc ID: 210966), each single tumor cell suspension was incubated with an individual oligo-labeled multiplex tag antibody directed to mouse MHC-I (BD Mouse Single Cell Sample Multiplexing Kit; Cat. 626545). Subsequently, labeled cells were pooled and incubated with a panel of oligo-labeled AbSeq antibodies listed in Supplementary Table S4. Thereafter, cells were stained with 2 mM Calcein AM (Thermo Fisher Scientific, Cat. C1430) and 0.3 mM Draq7 (Cat. 564904) for viability evaluation. About 20,000 cells were loaded on a BD Rhapsody Cartridge (Cat. 400000847) followed by Cell Capture Beads (Cat. 650000089). After cell lysis, the beads with attached cell-specific mRNAs and antibody-specific oligos were extracted and cDNA was generated. Libraries were prepared according to the BD Rhapsody™ System mRNA Targeted, Sample Tag, and BD® AbSeq Library Preparation Protocol (Doc ID: 214508), with a BD Rhapsody™ Immune Response Panel Mm (Cat. 633753) supplemented by an additional customized gene panel (see Supplementary Table S5.).

The sequencing was performed on an Illumina NextSeq 550 by the Genomics Core Facility of the Philipps University Marburg. Raw data was aligned and converted into an expression matrix using the BD Rhapsody Seven Bridges Analysis platform using the “BD Rhapsody™ Targeted Analysis Pipeline (Revision 15)” App. Further analysis was performed using scanpy [wolf_scanpy_2018] and cirrocumulus [li2020cumulus] [55].

### Bulk RNA sequencing of organoids and bioinformatics analysis

RNA was isolated using RNeasy Plus kit (Qiagen, Hilden, Germany, [Cat. / ID: 74034]). RNA quality was assessed using the Bioanalyzer RNA 6000 Nano Kit (Agilent, Santa Clara, California, USA). RNAseq libraries were prepared from total RNA with the QuantSeq 3′ mRNA-Seq Library Prep Kit FWD for Illumina (Lexogen, Vienna, Austria) in combination with the UMI Second Strand Synthesis Module for QuantSeq FWD (Illumina, Read 1, Lexogen) according to the manufacturer’s instructions. The quality of sequencing libraries was controlled on a Bioanalyzer 2100 using the Agilent High Sensitivity DNA Kit (Agilent). Pooled sequencing libraries were quantified and sequenced on the NextSeq550 platform (Illumina) with 75 bases single reads. Raw reads were aligned to the *Mus musculus* genome (genome sequence and gene annotation from Ensembl version 104) using STAR version 2.7.10 [Dobin2013]. PCR-duplication artifacts were filtered using unique-molecular-identifiers provided by the QuantSeq protocol using umi-tools version 1.1.2 [smith2017umi]. Reads were quantified with exons of protein-coding transcripts using custom Python scripts. Due to the low number of useable reads, genes were filtered to those having a) more than 0 reads in all samples and b) at least 10 reads in one condition before applying statistical analysis using edgeR (version 3.38.0) [Robinson2009], in paired sample mode to block batch effects. For visualization, ‘counts per million’ normalization was used.

Reactome and gene ontology databases were utilized to perform functional pathway enrichment analyses and investigate biological processes, respectively.

### RNA isolation, RT-qPCR, and PCR

RNA from frozen tumor tissue and cell lines were isolated using NucleoSpin RNA kit (Macherey-Nagel, Düren, Germany) or RNeasy Plus kit, respectively, according to manufacturers’ protocols. cDNA synthesis was performed using Revert Aid first strand cDNA synthesis kit (Thermo Fisher Scientific, Waltham, Massachusetts, USA). The qPCR analyses were performed using Agilent’s Real Time PCR (qPCR) (Mx3000) and the PCR using Bio-Rad T100 thermal cycler (Bio-Rad Laboratories, Hercules, California, USA). The *Rpl32/RPL32* genes were used for normalization and the relative expression level of genes of interest was calculated by the ΔΔCt-method. The primer sequences are listed in Supplementary Table S2.

### KPC mouse organoids

Pancreatic cancer organoids were derived from the KPC mouse model according to published procedures [56]. The KPC organoids (5000 cells in 20 µL Matrigel per 96 well) were cultured in organoid growth medium [56] plus the secretome (ratio 1:1) derived from mouse MCs pre-treated according to the following conditions:

1. PBS: Mast cells pre-treated with PBS.
2. KO-EVs: Mast cells pre-treated with Bag6 KO EVs.
3. WT-EVs: Mast cells pre-treated with Bag6 WT EVs.
4. KO-EVs + αIL33: Mast cells pre-treated with anti-Il33 (50 ng/mL) antibody (Thermo Fisher, PA5-96929, USA) and Bag6 KO EVs.
5. WT-EVs + αIL33: Mast cells pre-treated with anti-Il33 (50 ng/mL) antibody (Thermo Fisher, PA5-96929, USA) and Bag6 WT EVs.
6. KO-EVs + αPDGF: Mast cells pre-treated with Bag6 KO EVs, thereafter, the anti-PDGF (20 ng/mL) antibody (06-127, Merck, Germany) was added to the secretome.

After 5 days, phase contrast images were taken by confocal microscopy (Leica DMI3000 B), and cell percentage was assessed using the Cell Titer 3D GLO kit (Promega, Walldorf, Germany), and control PBS was set to 100%.

### Human organoids

Human organoids were derived from PDAC tumor biopsies (University Hospital Marburg) and cultured in 96 well plates (2500 cells per well). The organoids were washed twice with PBS. TrypLE™ Express Enzyme (Thermo Fisher Scientific) was added into the respective wells and the plate was incubated at 37 °C, 5% CO_2_ for 5–8 min to dissociate the organoids into single cells. Afterward, the basic medium (Advanced DMEM/F12, HEPES, Glutamax, all from GIBCO, order Nr. 12634010, 15-630-080, 35050061, respectively) was added to stop the reaction and collect the organoids into 1.5 mL tubes. The tubes were centrifuged at 400 × *g* for 5 min. The supernatant (SN) was removed, and cell pellets were resuspended in 500 µL of basic medium. Cells were counted in a hemocytometer and seeded in 10 µL Matrigel (2500 cells/well) in a 96-well plate. The organoids were then cultured in organoid growth medium plus the secretome (ratio 1:1) derived from human MCs pre-treated according to the same condition described in KPC mouse organoids. After 7 days, phase contrast images were taken by confocal microscopy (Leica DMI3000 B) and cell percentage was assessed using the Cell Titer 3D GLO kit (Promega), control PBS was set to 100%. The organoids growth medium contained the following components A83-01 (R&D Systems, 2939/10); mEGF (PeproTech, *Cranbury, New Jersy, USA*, 315-09-100ug); mNoggi (PeproTech, 120-10C-100); hFGF10 (PeproTech, 100-26); Y-27632 (PeproTech, 1293823-10 mg); Gastrin I (Sigma-Aldrich, G9020-250UG); N-acetylcysteine (Sigma–Aldrich, A9165-5G); Nicotinamide (Sigma-Aldrich, 72340-100G); B-27 Supplement (GIBCO, 17504044); Wnt3a (Home Made); R-Spondin I (Home Made); Advanced DMEM/F12 (GIBCO, 12634010)].

### Fibroblast stimulation

Pancreatic stellate cells (mPSC) kindly provided by A. Neesse [53] were maintained in T75 flasks and cultured in DMEM with 10% FBS. Cells were split every 4–7 days. Cells were harvested after trypsinization (using 1x Trypsin/EDTA, Sigma T-4174) and reseeded at a concentration of 2.5 × 10^4^ cells/mL. To generate the quiescent (q)PSC, cells were embedded in a 70 μL Matrigel drop (Growth Factor Reduced (GFR) Basement Membrane Matrix, Corning, 356230) in a 1:1 ratio with DMEM (10% FBS) on a 3.5 cm suspension dish (Sarstedt). The embedded Matrigel was cultured in 0.5% FBS-containing DMEM for 48 h (starvation). Thereafter, the medium was replaced by PBS or the secretome of mouse MCs (pre-treated with Bag6 KO-EVs or Bag6 WT-EVs or MC medium as a control). After 48 h, we performed RT-qPCR to determine the phenotype of the fibroblasts. Each drop was harvested in ice-cold PBS. After centrifugation (300 × *g*, 4 °C, 5 min) the cell pellet was resuspended in ice-cold PBS and incubated for 30 min at 4 °C. Finally, the cells were centrifuged (300 × *g*, 4 °C, 5 min), and pellets were frozen at −80 °C until further analysis. The experimental design is summarized in (Fig. S6B). Primer sequences are listed in Supplementary Table S2.

### Il33 binding assay

Mouse MCs were cultured in 48-well plates (3.5 × 10^5^ cells/well) and incubated with PBS (negative control), Bag6 WT-EVs, Bag6 WT supernatant (crude SN), Bag6 KO-EVs or Bag6 KO crude SN isolated from Pan02 cells. MCs were harvested after 1 h of incubation, prior to flow cytometry to measure Il1rl1/Il33 receptor and Cd117 (control) surface expression (BD FACS Canto II*)*. Antibodies are listed in Supplementary Table S6.

### Flow cytometry beads analysis

To detect the EV-associated Il33 via flow cytometry bead assay, the same number of EVs (around 5 × 10^9^ particles/samples in 20 µL PBS) from Bag6 WT and Bag6 KO were incubated with Polybead® Microspheres 4.50 µm (Polysciences Europe, GmbH, Hirschberg an der Bergstrasse, Germany) overnight at 4 °C. The next day, EVs were blocked with equal volumes of 2% bovine serum albumin (BSA) in PBS (Carl Roth, Karlsruhe, Germany) for 2 h at RT. The EV-bead mixture was washed with FACS buffer (PBS + 1%FBS) and centrifuged for 5 min at 2000 rpm. The EVs were then incubated with anti-Il33 or with the isotype control for 1 h. Then, samples were washed with FACS buffer and centrifuged for 5 min at 2000 rpm. Finally, the samples were resuspended in 500 µL FACS buffer and measured by flow cytometry (BD FACS Canto II*)*. FlowJo (version 10.6.1, BD Bioscience) was used for analysis. To measure ST2/Il1rl1 expression on Bag6 WT/KO Pan02 cells and MCs, the cells were stained with ST2/Il1rl1 antibody, and the geometric mean of fluorescence was measured by flow cytometry. Antibodies are listed in Supplementary Table S6.

### Statistics

Graphical representation of the data was obtained using GraphPadPrism® software (version 9.1; Graph Pad Software, La Jolla, California, USA) and FlowJo (version 10.6.1, BD Bioscience). Significance differences for the in vivo and in vitro experiments were evaluated using statistical tests as indicated in the figure legends. All data are biological replicates and represent at least 2 independent experiments.

### Supplementary information

Original blots 0064R

Supplementary files

Supplementary figure S1

Supplementary figure S2

Supplementary figure S3

Supplementary figure S4

Supplementary figure S5

Supplementary figure S6

Supplementary figure S7

Table S1

Table S2

Table S3

Table S4

Table S5

Table S6

## Data Availability

Mass spectrometric raw data, full analysis code with package versions/settings and statistical output were uploaded to the ProteomeXchange Consortium via the MassIVE partner repository (https://massive.ucsd.edu/) with the identifier PXD047563 (MassIVE ID: MSV000093583; 10.25345/C58P5VM5M) and available using a password: alhamwe_Bag6. Raw RNAseq data was deposited at EBI ArrayExpress under accession E-MTAB-13570 and the scRNAseq expression matrix was deposited under E-MTAB-13573. Data are available using the following links: E-MTAB-13570: E-MTAB-13573: All other data are available in the manuscript and the Supplementary Materials. Please contact the corresponding author for any additional information.
