# Supplementary material for: BAG6 restricts pancreatic cancer progression by suppressing the release of IL33-presenting extracellular vesicles and the activation of mast cells
Source: Cell Mol Immunol. 2024 Jun 28;21(8):918–31. doi: 10.1038/s41423-024-01195-1 (PMC11291976; doi:10.1038/s41423-024-01195-1)
Supplement: Supplementary file 1 — Original blots 0064R [file 41423_2024_1195_MOESM1_ESM.pptx]

## Slide 1
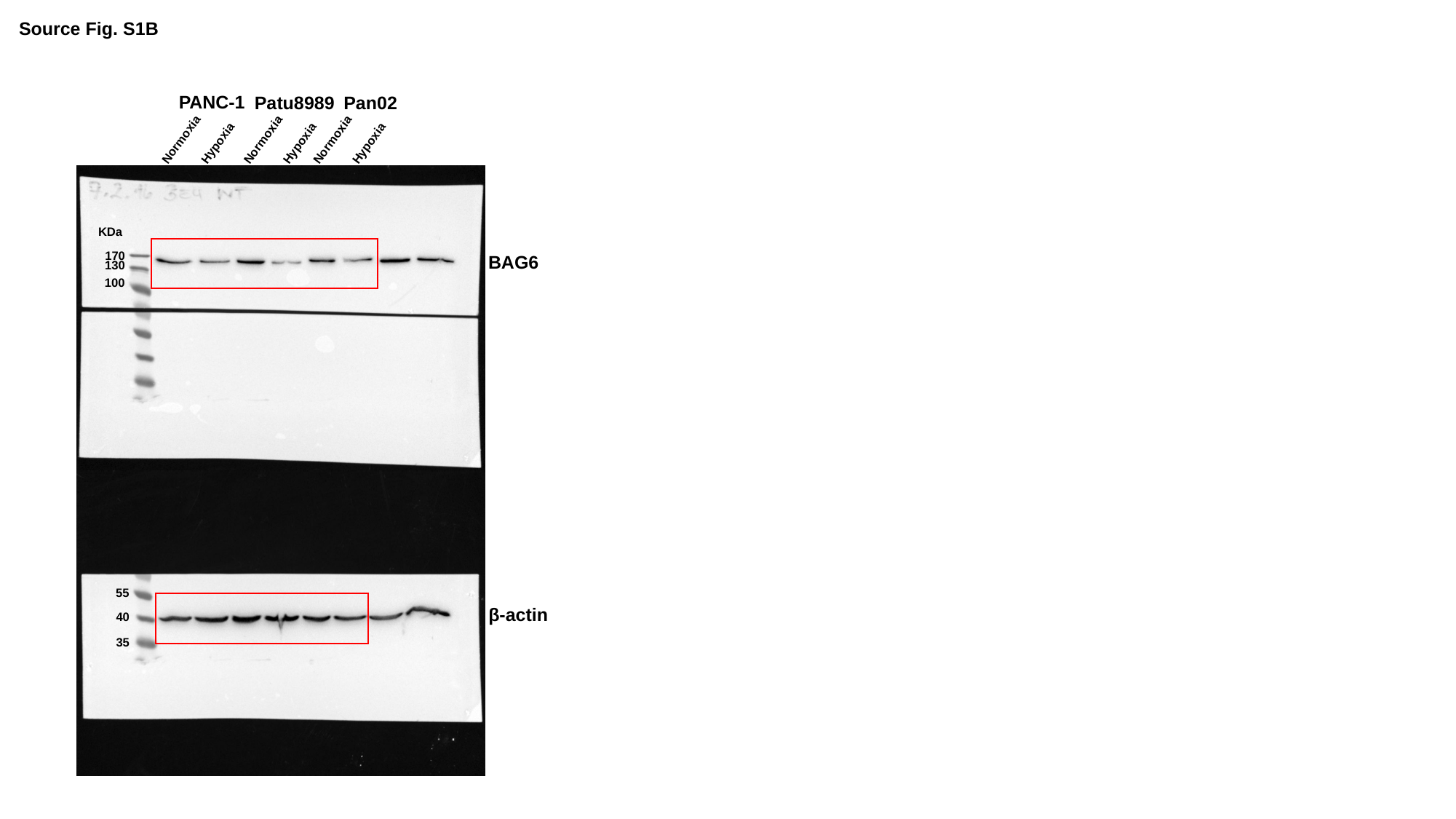

Source Fig. S1B
PANC-1
Patu8989
Pan02
Normoxia
Hypoxia
Normoxia
Hypoxia
Normoxia
Hypoxia
KDa
170
BAG6
130
100
55
β-actin
40
35

## Slide 2
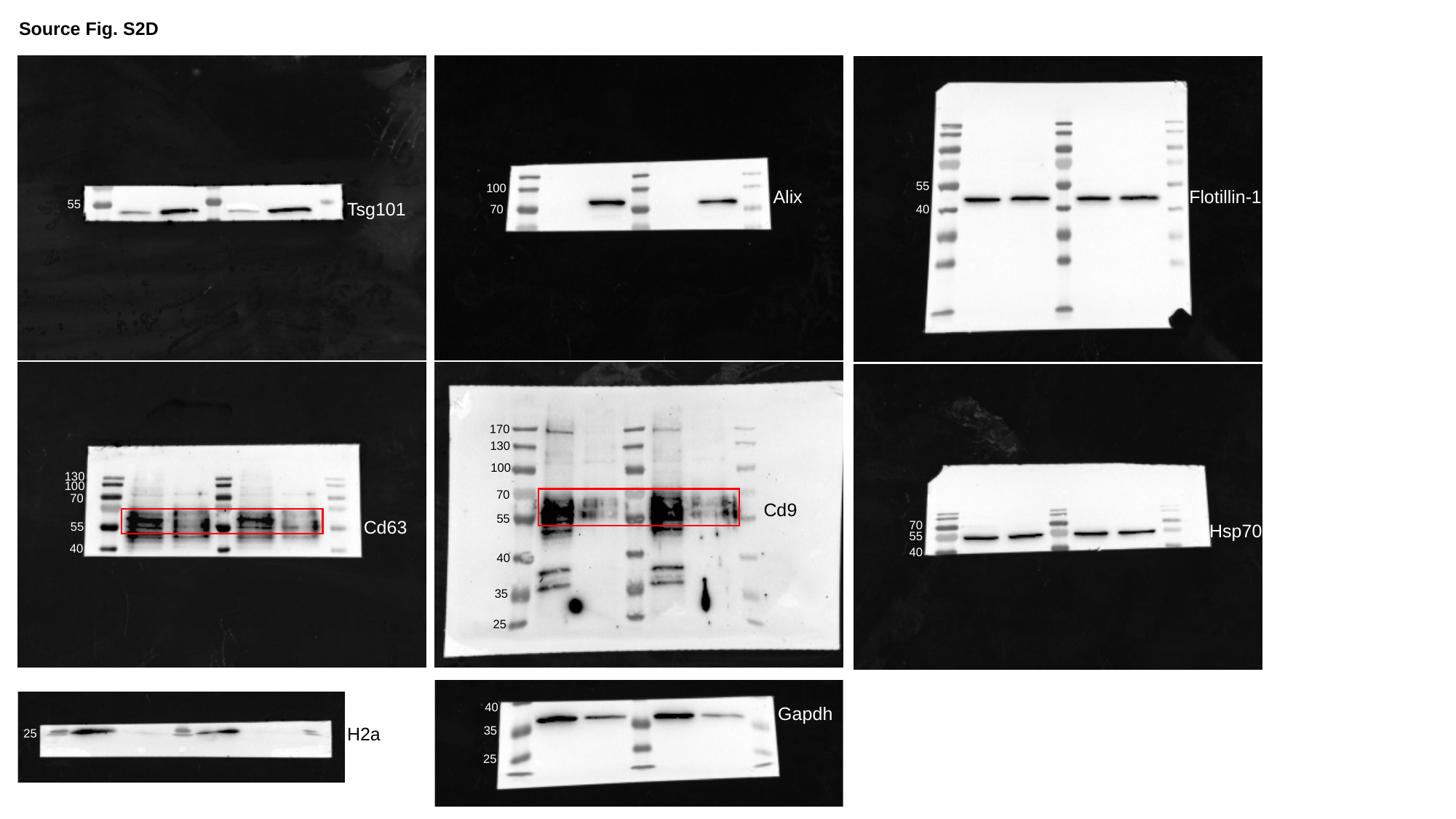

Source Fig. S2D
55
100
Flotillin-1
Alix
55
Tsg101
70
40
170
130
100
70
55
40
35
25
Cd9
130
100
70
Cd63
70
55
Hsp70
55
40
40
40
Gapdh
H2a
35
25
25

## Slide 3
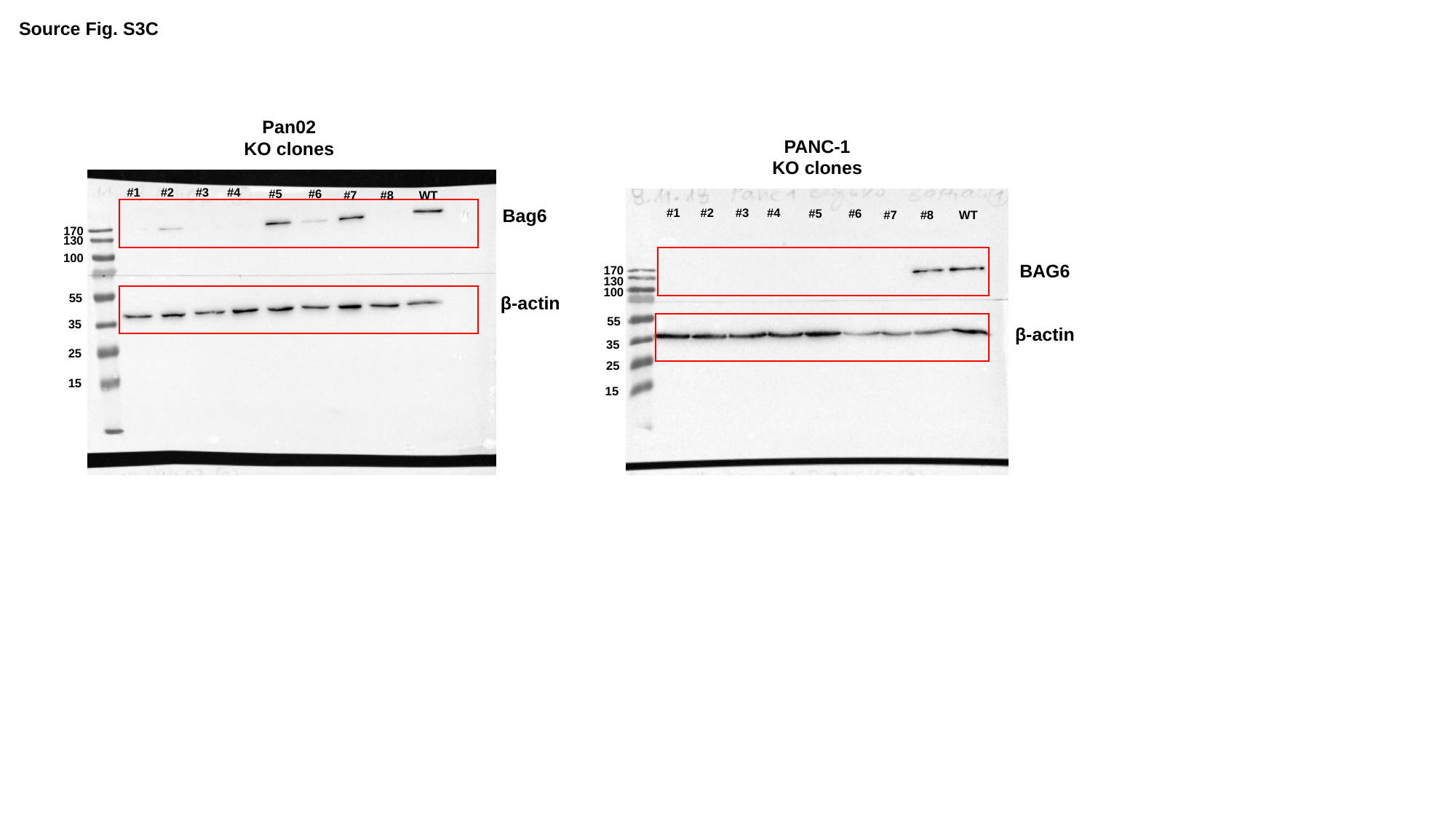

Source Fig. S3C
Pan02
KO clones
#1
#2
#3
#4
#5
#6
#7
#8
WT
Bag6
170
130
100
55
β-actin
35
25
15
PANC-1
KO clones
#1
#2
#3
#4
#5
#6
#7
#8
WT
BAG6
170
130
100
55
β-actin
35
25
15

## Slide 4
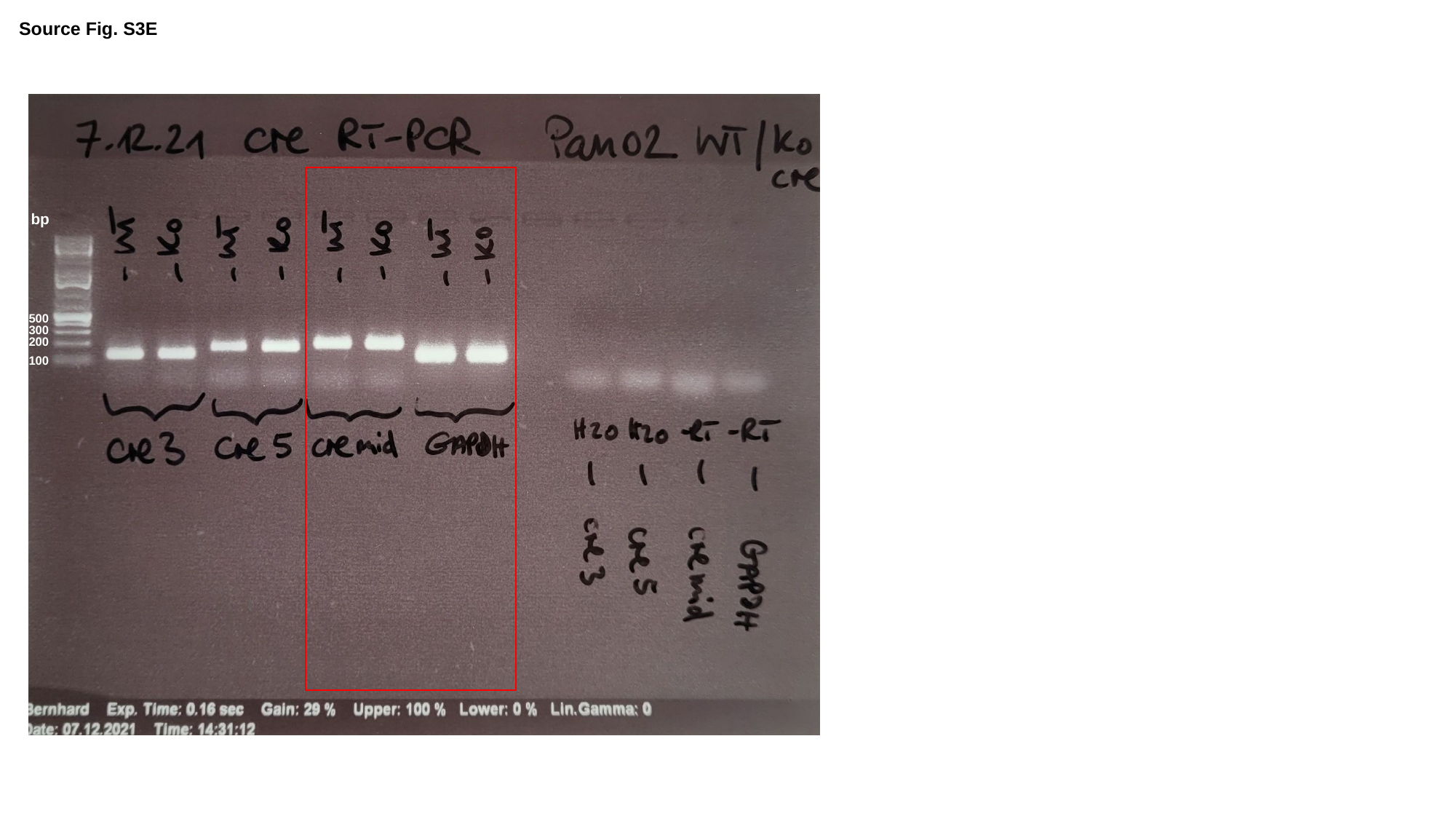

Source Fig. S3E
bp
500
300
200
100

## Slide 5
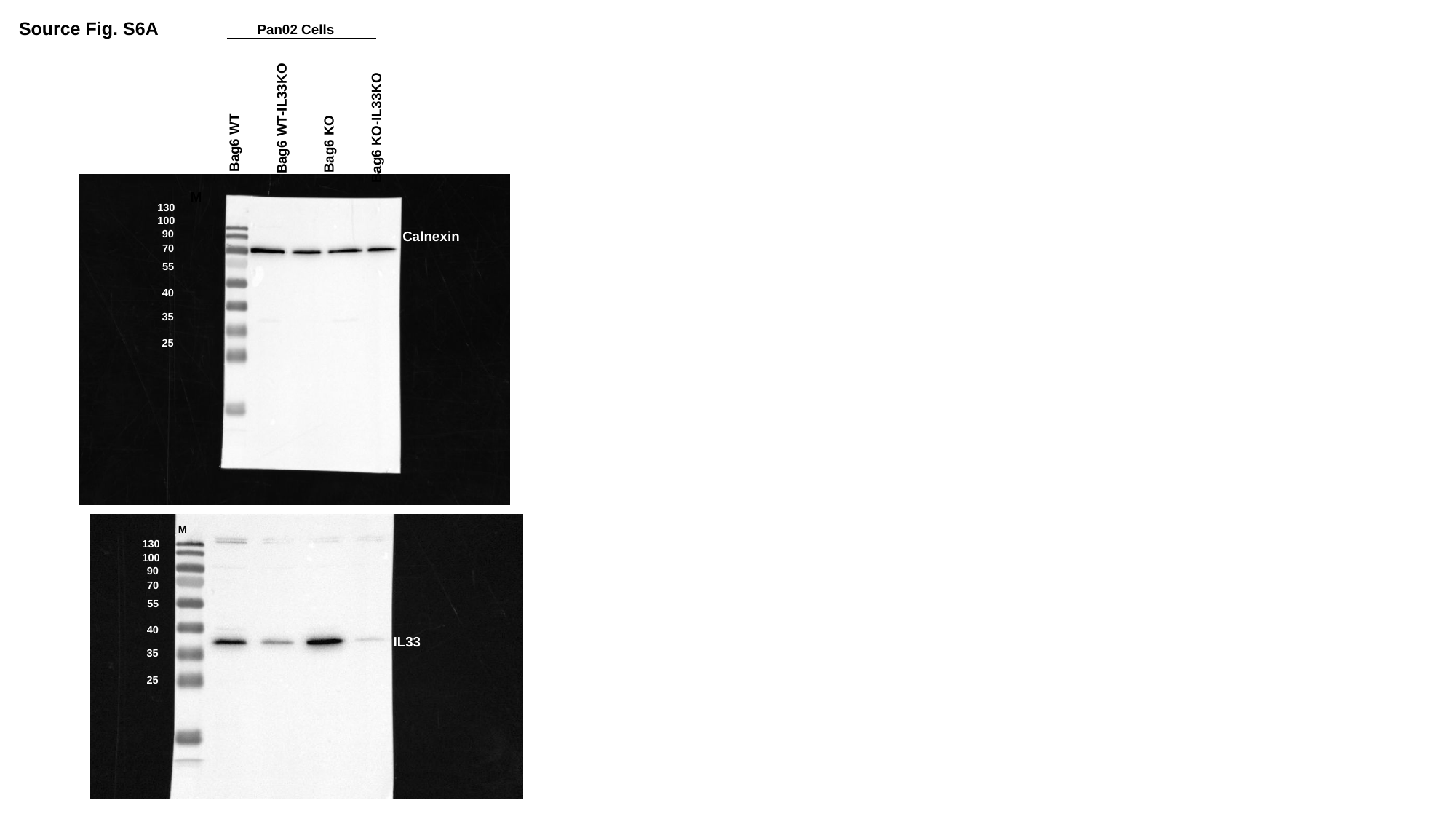

Source Fig. S6A
Pan02 Cells
Bag6 WT-IL33KO
Bag6 KO-IL33KO
Bag6 WT
Bag6 KO
M
130
100
90
Calnexin
70
55
40
35
25
M
130
100
90
70
55
40
IL33
35
25
