# Supplementary material for: BAG6 restricts pancreatic cancer progression by suppressing the release of IL33-presenting extracellular vesicles and the activation of mast cells
Source: Cell Mol Immunol. 2024 Jun 28;21(8):918–31. doi: 10.1038/s41423-024-01195-1 (PMC11291976; doi:10.1038/s41423-024-01195-1)
Supplement: Supplementary file 2 — Supplementary files [file 41423_2024_1195_MOESM2_ESM.docx]

**Supplementary summary:**

The supplementary files comprise:

1. Supplementary materials and methods for EVs isolation, characterization, EVs proteomics, EVs-IL33 ELISA, immunoblotting, immunohistochemistry, and immunofluorescence.
2. Supplementary figure legends for figures S1, S2, S3, S4, S5, S6 and S7.
3. Supplementary tables (1-6), containing lists of antibodies, primers, patient information, and Olink data for HMCs.

**Supplementary Material and Methods:**

**EV isolation and characterization**

EVs were collected from the supernatant (SN) of Pan02 and PANC-1 cells after 48 h of culturing (1% O_2_) in CD293 medium (Gibco). Afterward, sequential centrifugation steps of the SN were performed as previously described (1). Size, concentration, and expression of EV markers CD9, CD81, and CD63 were measured by nano-flow cytometry (see Supplementary Table S6). 1x10^9^ particles were used for staining and 3-5 x10^9^ particles/ml were applied for *in vitro* experiments.

**EV Proteomics**

Approximately 3x10^10^ EVs in 30 μl PBS (1x10^12^ EVs/ml) from Bag6 WT and KO cells were subjected to proteomic profiling in sextuplicate at the Core Facility Translational Proteomics at Philipps University Marburg. Using 1:1 dilution with 4% N-Lauroylsarcosine sodium salt (Sigma-Aldrich, Merck KGaA, Darmstadt, Germany, final concentration of 2%) in 50 mM TEAB buffer (Sigma-Aldrich), EVs were lyzed incubating for 10 min at 90 °C and constant shaking. Following protein content estimation using BCA (ThermoFisher Scientific, Germany), 50 μg protein equivalent to 4x10^9^ – 1.5x10^10^ particles per sample were volume adjusted, reduced (addition of 2 μl 400 mM dithiothreitol to a final concentration of 10 mM and incubation at 95 °C for 10 min), and alkylated (additions of 2 μl 550 mM iodoacetamide to a final concentration of 13 mM and incubation for 30 min at 25 °C). Subsequently, samples were diluted 7x with 50 mM TEAB buffer to allow trypsin digestion. 0.6 μg of Trypsin was added, digestion proceeded for 16 h at 37 °C and was stopped by addition of trifluoroacetic acid (TFA) to a final concentration of 1.5%. Precipitated remnant SLS was removed by centrifugation. Peptides were purified using solid phase extraction on C18 microspin columns according to the manufacturer’s protocol (Macherey-Nagel, Germany). Purified peptides were dried and resuspended in 100 μl of 0.1% TFA.

Purified peptides were analyzed by liquid chromatography–tandem mass spectrometry (LC/MS2) carried out on a Bruker Daltonics timsTOF Pro instrument connected to a Bruker Daltonics nanoElute instrument. An estimated 200 ng of peptides (equivalent to between 1.4x10^7^ and 4.9x10^7^ particles) were loaded onto a C18 precolumn (Thermo Trap Cartridge 5mm, µ-Precolum TM Cartridge / PepMap TM C18, Thermo Scientific) and eluted in backflush mode using a gradient from 98% solvent A (0.15% formic acid) and 2% solvent B (99.85% acetonitrile and 0.15% formic acid) to 17% solvent B over 60 min, continued from 17 to 25% of solvent B for another 30 min, then from 25 to 35% of solvent B for another 10 min over a reverse-phase high-performance liquid chromatography (HPLC) separation column (Aurora Series Emitter Column with CSI fitting, C18, 1.6 μm, 75 μm×25 cm, Ion Optics) with a flow rate of 400 nL/min. The outlet of the analytical column was directly coupled to the MS instrument using a captive spray fitting. Data were acquired using a default method provided by Bruker under a data-dependent acquisition (DDA) paradigm (DDA PASEF-satndard_1.1sec_cycletime.m). In short, spectra were acquired with fixed resolution of 45,000 and mass range from 100 to 1700 m/z for the precursor ion spectra and a1/k0 range from 0.6 to 1.6 Vs/cm^2^ with 100 ms ramp time for ion mobility.

Spectrum identification: Peptide spectrum matching and label-free quantitation were performed using MaxQuant (version 2.0.3.0) against the *Mus musculus* UniProt database (17110 entries, March 2022) with default settings. In brief, output was filtered to a 1% false discovery rate on peptide and protein levels, both. Tryptic cleavage was allowed following K*, R*, with a maximum of 2 missed cleavages. Cysteine carbamidomethylation was included as a fixed modification and methionine oxidation and asparagine and glutamine deamidation (maximum of two) as a variable modification. Relative quantitation used the label free quantitation algorithm included with MaxQuant using default settings.

Downstream differential expression analysis was performed using the limma-based (2) in house package autonomics (Bhagwat et al., 2023) (19) In brief, the MaxQuant output table “proteinGroups.txt” was loaded into R. One wild type sample was found to be a massive outlier with respect to identified protein groups and excluded from further analysis. Missing values were imputed by random numbers drawn from a normal distribution with a width of 0.3 and down shift of 3 standard deviations for each group separately. Imputation was only performed on systematic NAs (missing not at random), defined as protein groups in which none of the replicates for a given condition had any values. Additionally, at least 50% of replicates in the other condition had to contain a quantification value in order for the missing values to be imputed. Imputation is indicated in the final result tables. TheseThese mMass spectrometric raw data, full analysis code with package versions/settings and statistical output were uploaded to the ProteomeXchange Consortium via the MassIVE partner repository (https://massive.ucsd.edu/) with the identifier PXD047563 (MassIVE ID: MSV000093583; doi:10.25345/C58P5VM5M) and available using a password: alhamwe_Bag6

**PEA technology (Olink Explore 3072 analysis)**

The Proximity Extension Assay (PEA) technology uses a dual-recognition approach in which two matched antibody pairs bind different epitopes of the target protein. The antibodies are conjugated to oligonucleotide labels that can hybridize upon target recognition when both antibodies are in close proximity. The short dsDNA is pre-amplified and further extended with sample-specific barcodes. The abundance of resulting amplicons is proportional to the protein concentration in the sample and allows a semi-quantitative analysis. The protein levels are reported as Normalized Protein eXpression (NPX), an Olink-specific arbitrary unit on a log2 scale.

Proteins in the secretome of human mast cells stimulated with BAG6 KO PANC-1-EVs (n=2 biological replicate) and in the secretome of PBS-treated human mast cells, as negative control (n=1) were analyzed using Olink Explore 3072 workflow at the Core Facility Translational Proteomics, Philipps University Marburg, All samples were analyzed following the Olink standard protocol (v1.5, 2022-12.21) as previously described (3).

Next Generation Sequencing (NGS) of the libraries was performed at the Genomics Core Facility, Philipps University Marburg.

**EV-Il33/IL-33 ELISA**

IL33 levels were measured by ELISA (R&D Systems, Minneapolis MN, USA) according to the manufacturer's instructions. An equal number of EVs (BAG6 WT and BAG6 KO) and soluble fractions isolated from Pan02 and PANC-1 cell supernatant (1-2 x 10^11^ particles/ml) were applied. For IL33 ELISA in Fig.6 cells were treated with KIF or GW4869 as described in the figure legends. SN was collected and cells were lysed in lysis buffer (50 mM Tris-HCl pH 8, 150 mM NaCl, 0.5% Triton X-100, 0.5% protease inhibitors).

**Immunoblotting**

EVs and cells were lysed in lysis buffer (50 mM Tris-HCl pH 8, 150 mM NaCl, 0.5% Triton X-100, 0.5% protease inhibitors) for 15 min on ice and the protein concentration was determined by BCA Protein Assay Kit (Thermo Fisher Scientific). 20-25 µg protein were subjected to Western blotting. Briefly, protein samples were separated by SDS-PGE and transferred onto nitrocellulose membranes. The membranes were then incubated with primary antibodies (see list in Supplementary Table S4) overnight at 4 °C. Secondary antibodies used were mouse IgG HRP-linked (Cell Signaling, #7076),rabbit IgG HRP-linked (#7074), and Goat anti-Mouse IgG (Neta Scientific, # IRDye® 680RD). The proteins were visualized in a ChemiDoc system (BioRad) after incubation with Luminata Forte Western HRP Substrate (Merck). All antibodies are listed in Supplementary Table S4.

**Immunohistochemistry of mouse tumor tissue and human tissue microarray (TMAs)**

Mouse tumor tissue was processed by embedding it in paraffin blocks and sectioned into slices of 7 μm thickness for subsequent staining. For human samples, TMA blocks were cut into 2 μm sections and mounted on SuperFrost Plus slides for immunohistochemical staining.

For immunohistochemistry staining, a heat-induced epitope retrieval with EDTA was applied. Staining was performed using a Dako Autostainer Link 48 for human tissue. After blocking endogenous peroxidase activity, sections were incubated for 45 min with mouse monoclonal anti-human CD4 antibody (1:400; Dako M7310, clone 4B12), mouse monoclonal anti-CD8 antibody (1:100; Dako M7103, clone C8/144B), mouse monoclonal anti-CD56 antibody (1:100; Dako M7304, clone 123C3), mouse monoclonal anti-Alpha Smooth Muscle (αSMA) antibody (1:200; Progen 61001, clone 1A4/ASM-1). Rabbit polyclonal anti-CD117 antibody (1:200; Dako A4502) and mouse monoclonal BAG6 antibody (3E4; raised against N-terminus 1:100; self-made) (4) were used for mouse and human samples. Sections were washed and treated with Dako REAL EnVision HRP Rabbit/Mouse polymer, designed to react with DAB chromogen, according to the manufacturer’s protocol for mouse tumor staining and ABC-method for human tissue. All stained tissues were scanned, and a blinded analysis of digitized histology and staining results was performed using the advanced QuPath bioimaging analysis software (version 0.3.2). The staining intensity of BAG6 in TMAs was graded into high or low expression. Antibodies are listed in Supplementary Table S4.

**Immunofluorescence (mouse tissue and organoid)**

The paraffin section slides from tumors were distributed in staining racks and then washed with Xylene (Roth, Karlsruhe, Germany) (15 min), isopropanol (Roth) (5 min), and a series of ethanol concentrations: 96%, 80% and 70% (3 min each). Slides were washed with washing solution (PBS +0.1 % Tween20) and antigen retrieval proceeded (35 min) using 1X citrate pH 6, in a steam cooker at 60 °C. The slides were rinsed in washing solution, blocked in 10% FBS for 1 h at RT, and then incubated with goat anti-mouse anti-GFP antibody (1:250) (Rockland, 600-101-215S, USA) overnight at 4 °C. The next day, the slides were washed twice with washing solution and incubated for 2h at RT with secondary antibody (rabbit anti-goat Alexa Fluor™ 488, Thermo Fisher, A-21222). Afterward, the slides were washed twice with washing solution and mounted with VECTASHIELD® Antifade Mounting Medium with DAPI (Vector Laboratories, Newark, California, USA, H-1200-10).

Slides were analyzed by laser confocal microscopy (Leica SP8i) (Leica Microsystems, Wetzlar, Germany) at the Microscopy Core Facility, Philipps University Marburg.

Organoids were fixed for 40 min with 4% PFA on ice, overlaid with acetone for 10 min, and blocked for 2 h in 5% FBS/ PBS at 4 °C. The organoids were then incubated with anti-Ki67 antibodies (Biolegend 398502) in 5% FBS/ PBS for 24 h, washed, and incubated with anti-rat Alexa 647 (A21472, Invitrogen) for 18 h at 4 °C. Nuclei were stained with Hoechst 33342 and actin with Alexa 546 phalloidin (No A22283, Invitrogen). Following 4 h at room temperature, the organoids were mounted with Mowiol. Confocal images were acquired on a Leica STELLARIS equipped with a 40x oil planapochromat objective (Leica Microsystems). Antibodies are listed in Supplementary Table S4.

**Trasmission electron microscopy (TEM) of EVs isolated from Bag6 WT/KO Pan02 cells**

Formvar-coated copper grids (Science Services, München) were prepared with (1:20) of 5 µl diluated sampels. Varying amounts of protein were included in the sampls, as determined by the BCA protein assay: 0.17 µg (Serum 10k xg), 0.24 µg (L-540 10k xg), 0.35 µg (L-540 100k xg), or 0.78 µg (Serum 100k xg). Then the sampels were placed on the grids for 20 minutes followed by fixation for 5 minutes with 2% paraformaldehyde. The samples washed with PBS and fixed again for 5 minutes with 1% glutaraldehyde. Finally, the samples were washed with Milli-Q water and contrasted for 4 minutes with 1.5% uranyl acetate. Imaging was performed using a Gatan OneView 4K camera mounted on a Jem-2100Plus (Jeol) operating at 30kx and 100kx. ImageJ software (NIH) used to generate the photos.

**Supplementary figure legends**

**Figure S1: Low BAG6 expression correlated with shorter survival and hypoxic conditions in PDAC**

**(*A*)** Bar diagram to compare the BAG6 protein plasma level and overall survival (y axis, month) of PDAC patients. BAG6 was quantified using Olink Explore 3072 analysis. Patients (n=46, for patient characteristics see Supplementary Table S3) were classified into two groups: high BAG6 in plasma (Normalized Protein eXpression (NPX), an Olink-specific arbitrary unit on a log2 scale ≥ 0.72, in blue) and low BAG6 in plasma (NPX ≤ 0.72, in Red). ***(B, C)*** Immunoblot and quantified protein expression intensity analysis of BAG6 and β-actin using the cell lysates from PANC-1, Patu 8988t (PDAC human cell lines), and Pan02 (PDAC mouse cell line). The cells were cultured under hypoxic or normoxic conditions, from BAG6 WT genetic backgrounds. The BAG6 protein intensity in normoxic conditions was set to 1 (n ≥ 3 per condition). ***(D, E)*** Tumor growth curve and Immune cells counted per square millimeter of tumor area of s.c. tumors on day 9 (mean ± SEM, n= 3). Data in *(****A,C,D and E****)* are mean ± SEM. Statistical significance was determined using unpaired Mann-Whitney U tests;* *P* < .05; ** *P* < .01, ns: not significant.

**Figure S2: Pan02-EVs characterization**

***(A***) Median particle size of Cd9+ EVs vesicles derived from Bag6 WT/KO Pan02 (n=6) and **(*B*)** percentage of tetraspanins positive events (Cd9, Cd63, and Cd81) (n=6). ***(C)*** size distribution of EVs-isolated from Bag6 WT/KO Pan02 cells using nanoparticle tracking analysis (Zetaview, Particle Metrix, Germany) (NTA) ***(D)*** Immunoblot analysis of EVs and cell lysate from Bag6 WT/KO Pan02 cells to detect EV markers Tsg101, Alix, Flotillin-1, Cd63, Cd9, Hsp70, and Gapdh. The nuclear marker H2a was used as a negative control. ***(E)*** Transmission electron microscopy (TEM) depicts the morphology of EVs isolated from Bag6 WT/KO Pan02 cells. Data in [***A, B***] are shown as mean ± SEM and significance was determined using unpaired Mann-Whitney U tests.

**Figure S3: Pan02-EVs proteomic profile**

***(A)*** Volcano plot depicting a differential analysis comparing KO EV and WT EV proteins. The x-axis represents the difference between conditions in a log2 scale (label free quantitation intensities), whereas the y-axis shows -log10 p-value. Samples labeled as ‘yes’ are samples with systematic imputation and ‘false’ means that samples were not imputed (statistical results are stored in**bag6-001_sextuplicates_stat_imp.tsv**).

Several of the KO EV enriched factors contribute to EVs biogenesis, cargo sorting, vesicle transport and release *e.g.* Washc4 (WASH complex subunit 4) (5), Cd151 (platelet and Endothelial Cell Tetraspan Antigen 3) (6), Tmed10 (transmembrane emp24 domain-containing protein 10) (7), and Lamp2 (lysosomal-associated membrane protein 2) (8). Some highly enriched proteins are cancer related, for example (i) Daam1 (dishevelled-associated activator of morphogenesis 1, associated with the Wnt signaling pathway) (9), (ii) Lbp (lipoprotein binding protein), a secreted protein which correlates with a worse prognosis in patients with gastric cancer (10) or (iii) Gys1 (glycogen synthase 1), a metabolic enzyme with tumor promoting activity in clear cell renal carcinoma) (11). WT EV enriched proteins represent pro- and anti-tumorigenic factors, among them the tumor suppressor factor Cdkn2c, a member of the INK4 family of cyclin-dependent kinase inhibitors that controls cell cycle progression (genecards.org). The enzyme TET2 (5-methylcytosine hydroxylase TET2) is known to be reduced in the squamous-like PDAC transcriptional subtype (12). ***(B)*** Full STRING network (edges indicate both functional and physical protein associations) to depict all proteins significantly up-regulated in KO EVs (69, string-dg.org). Several subunits of the vacuolar ATPase (V-ATPase), a multisubunit enzyme involved in ATP-hydrolysis-driven acidification of endosomes, lysosomes, and the trans Golgi network regulating synaptic vesicle loading (13) are clustered. ***(C)*** Immunoblot to identify BAG6 KO clones in Pan02 and PANC-1 cell clones upon gRNA transfection and selection. ***(D)*** *In vitro* growth curve of cre^+^ Bag6 WT/KO Pan02 pre-treated with/without GW4869 (2.5 mg/well) every second day (n=6). ***(E)*** Validation of cre expression in Pan02 WT and KO cells using PCR.

**Figure S4: *Cre-LoxP* recombination and workflow single-cell analysis**

***(A***) Schematic depiction of the *Cre-loxP* system used to monitor extracellular vesicle uptake *in vivo* ***(B)*** Workflow depicting sample acquisition, processing, and analysis from the tumor tissue of orthotopically transplanted mice with either Cre^+^ Bag6 WT or Cre^+^ Bag6 KO Pan02 cancer cells. ***(C)*** CellChat Cell-Cell Communication Atlas Explorer (14) of the Cre^+^ Bag6 KO tumor was utilized to demonstrate the interconnection networks between MCs and tumor cells, as well as MCs and non-malignant cells, depicted for Cd4^+^, fibroblasts, and macrophages. The intensity of the lines represents the interaction weight/strength.

**Figure S5: BAG6 expression intensity correlated with MC infiltration in tumor tissue**

***(A)*** UMAP projection shows the expression of representative MC markers and cytokines produced by activated MCs found in the tumor tissue of the Bag6 KO group. ***(B)*** *In vitro* growth curve of Cre^+^ Bag6 WT/KO Pan02 with or without Imatinib treatment (2.5 mg/well) (n=3) every second day. ***(C)*** Immunohistochemical staining of BAG6 protein performed in TMAs. Representative images of samples with high MC infiltration corresponded to low BAG6 protein staining intensity and vice versa. ***(D)*** Percent positive Il1rl1/ST2 cells based on scRNAseq data in WT and KO animal groups. Cells were considered positive above a log expression threshold of (1.5). ***(E)*** Geometric mean of expression of surface ST2/Il1rl1 *in vitro*, measured by flow cytometry in mouse mast cells (MC/9) and Bag6 WT/KO Pan02 cells. UMAP: Uniform Manifold Approximation and Projection.

**Figure S6: IL33 signaling and characterization of mast cells**

***(A)*** Immunoblot to detect Il33 in Bag6 WT/KO Pan02 cells upon IL33-gRNA transfection. Calnexin was used as control. ***(B)*** Cytokine gene expression (*Il6*,*Lif* and *Tnf*α) of MC stimulated with the following conditions: Bag6 WT- or KO-EVs isolated from Pan02 cells; Bag6 WT- or KO-EVs isolated from Pan02 after knocking out of *Il33*; Bag6 KO-EVs pre incubated with anti-Il33 antibody; Bag6 KO-EVs after pre incubating the mouse mast cells cells for 1 hour with anti-ST2/Il1rl2 antibody. Data were normalized to *Rpl32* (mean± SEM, n= 6-11, at least 2 independent experiments). ***(C)*** Schematic representation of the *in vitro* experiment designed to obtain the secretome of human MCs pre-treated with PANC-1 BAG6 KO-EVs or PBS. Secretome was harvested after 24 h and the proteomic profile was analyzed by Olink as described in the supplementary methods. ***(D)*** Normalized protein expression values (NPX) from the secretomes Olink Explore 3072 analysis were averaged (n=2 biological replicates) and an effect size was calculated as compared to a PBS control secretome sample. The resulting values were plotted using a kernel density estimate. Labeled proteins represent those with the highest effect size, see Fig. 6A. ***(E)*** Schematic representation of the KPC mouse and human PDAC organoids experiments, details are described in supplementary methods (n=3). ***(F)*** Differential gene expression analysis of KPC organoids depicting the significantly upregulated genes in organoids cultured with the secretome from MCs pre-treated with Bag6 KO-EVs compared with WT-EVs including *Il33* and *Cxcl5*, both correlating with poor survival and tumor progression in PDAC patients (15, 16), *Epcam* which is over-expressed on the basolateral membrane in PC and promotes migration, proliferation, and differentiation (17); or mediators promoting epithelial-to-mesenchymal transition like *S100a16* (18)*.* ***(G)*** Gene ontology and WiKi pathway analysis of the significantly upregulated genes in organoids cultured with the secretome from MCs pre-treated with Bag6 KO-EVs or WT-EVs.

**Figure S7: mPSC/fibroblasts *in vitro* experimental design**

Schematic representation of the mPSC *in vitro* experiment. mPSC were cultured in 0.5% FBS DMEM starvation medium as described in the supplementary methods. The secretome from mouse MCs, pre-treated with Bag6 WT-EVs, Bag6 KO-EVs, and PBS, was collected at the indicated time points. Subsequently, the secretome was cultured with matrigel-embedded qPSC. After two days, mRNA expression of depicted iCAF/mCAF gene candidates was evaluated by RT-qPCR.

**References**

1. Ponath V, Hoffmann N, Bergmann L, Mader C, Alashkar Alhamwe B, Preusser C, et al. Secreted Ligands of the NK Cell Receptor NKp30: B7-H6 Is in Contrast to BAG6 Only Marginally Released via Extracellular Vesicles. Int J Mol Sci. 2021;22(4).

2. Ritchie ME, Phipson B, Wu D, Hu Y, Law CW, Shi W, et al. limma powers differential expression analyses for RNA-sequencing and microarray studies. Nucleic Acids Res. 2015;43(7):e47.

3. Steitz AM, Schroder C, Knuth I, Keber CU, Sommerfeld L, Finkernagel F, et al. TRAIL-dependent apoptosis of peritoneal mesothelial cells by NK cells promotes ovarian cancer invasion. iScience. 2023;26(12):108401.

4. Reiners KS, Topolar D, Henke A, Simhadri VR, Kessler J, Sauer M, et al. Soluble ligands for NK cell receptors promote evasion of chronic lymphocytic leukemia cells from NK cell anti-tumor activity. Blood. 2013;121(18):3658-65.

5. Courtland JL, Bradshaw TW, Waitt G, Soderblom EJ, Ho T, Rajab A, et al. Genetic disruption of WASHC4 drives endo-lysosomal dysfunction and cognitive-movement impairments in mice and humans. Elife. 2021;10.

6. Malla R, Marni R, Chakraborty A. Exploring the role of CD151 in the tumor immune microenvironment: Therapeutic and clinical perspectives. Biochim Biophys Acta Rev Cancer. 2023;1878(3):188898.

7. Coulter ME, Dorobantu CM, Lodewijk GA, Delalande F, Cianferani S, Ganesh VS, et al. The ESCRT-III Protein CHMP1A Mediates Secretion of Sonic Hedgehog on a Distinctive Subtype of Extracellular Vesicles. Cell Rep. 2018;24(4):973-86 e8.

8. Ferreira JV, da Rosa Soares A, Ramalho J, Maximo Carvalho C, Cardoso MH, Pintado P, et al. LAMP2A regulates the loading of proteins into exosomes. Sci Adv. 2022;8(12):eabm1140.

9. Gao C, Chen YG. Dishevelled: The hub of Wnt signaling. Cell Signal. 2010;22(5):717-27.

10. Xie L, Qiu S, Lu C, Gu C, Wang J, Lv J, et al. Gastric cancer-derived LBP promotes liver metastasis by driving intrahepatic fibrotic pre-metastatic niche formation. J Exp Clin Cancer Res. 2023;42(1):258.

11. Chen SL, Huang QS, Huang YH, Yang X, Yang MM, He YF, et al. GYS1 induces glycogen accumulation and promotes tumor progression via the NF-kappaB pathway in Clear Cell Renal Carcinoma. Theranostics. 2020;10(20):9186-99.

12. Eyres M, Lanfredini S, Xu H, Burns A, Blake A, Willenbrock F, et al. TET2 Drives 5hmc Marking of GATA6 and Epigenetically Defines Pancreatic Ductal Adenocarcinoma Transcriptional Subtypes. Gastroenterology. 2021;161(2):653-68 e16.

13. Morel N, Poea-Guyon S. The membrane domain of vacuolar H(+)ATPase: a crucial player in neurotransmitter exocytotic release. Cell Mol Life Sci. 2015;72(13):2561-73.

14. Jin S, Guerrero-Juarez CF, Zhang L, Chang I, Ramos R, Kuan CH, et al. Inference and analysis of cell-cell communication using CellChat. Nat Commun. 2021;12(1):1088.

15. Huang X, Zhang G, Liang T. Pancreatic tumor initiation: the potential role of IL-33. Signal Transduct Target Ther. 2021;6(1):204.

16. Li A, King J, Moro A, Sugi MD, Dawson DW, Kaplan J, et al. Overexpression of CXCL5 is associated with poor survival in patients with pancreatic cancer. Am J Pathol. 2011;178(3):1340-9.

17. Patriarca C, Macchi RM, Marschner AK, Mellstedt H. Epithelial cell adhesion molecule expression (CD326) in cancer: a short review. Cancer Treat Rev. 2012;38(1):68-75.

18. Li T, Ren T, Huang C, Li Y, Yang P, Che G, et al. S100A16 induces epithelial-mesenchymal transition in human PDAC cells and is a new therapeutic target for pancreatic cancer treatment that synergizes with gemcitabine. Biochem Pharmacol. 2021;189:114396.

19. Bhagwat A, Cotton R, Hayat S, Graumann J (2023). autonomics: Generifying and intuifying cross-platform omics analysis. [doi:10.18129/B9.bioc.autonomics](https://doi.org/10.18129/B9.bioc.autonomics), R package version 1.10.2, <https://bioconductor.org/packages/autonomics>.
