# Supplementary figures and images for: BAG6 restricts pancreatic cancer progression by suppressing the release of IL33-presenting extracellular vesicles and the activation of mast cells
Source: Cell Mol Immunol. 2024 Jun 28;21(8):918–31. doi: 10.1038/s41423-024-01195-1 (PMC11291976; doi:10.1038/s41423-024-01195-1)

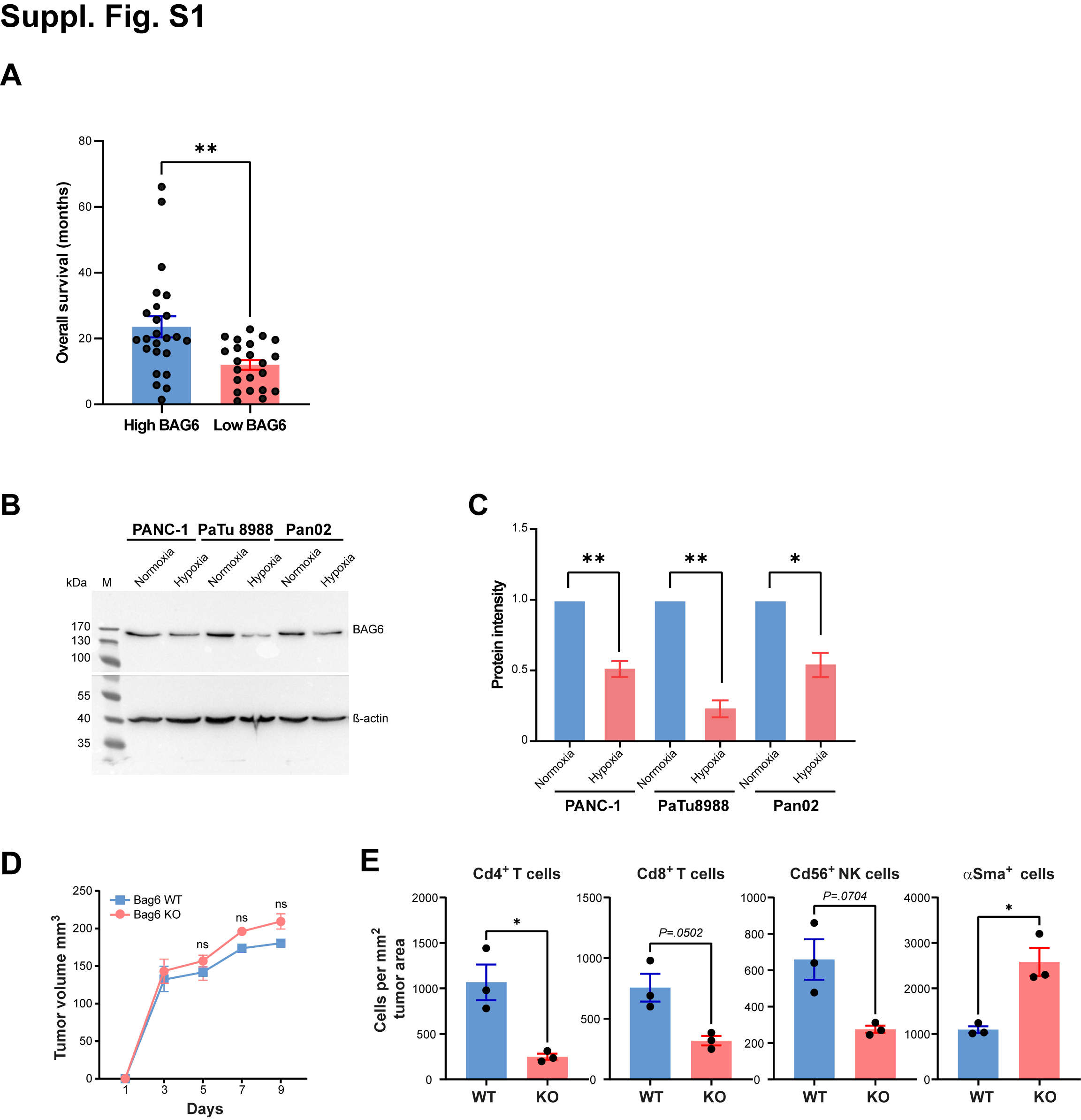

Supplement: Supplementary file 3 — Supplementary figure S1 [file 41423_2024_1195_MOESM3_ESM.jpg]

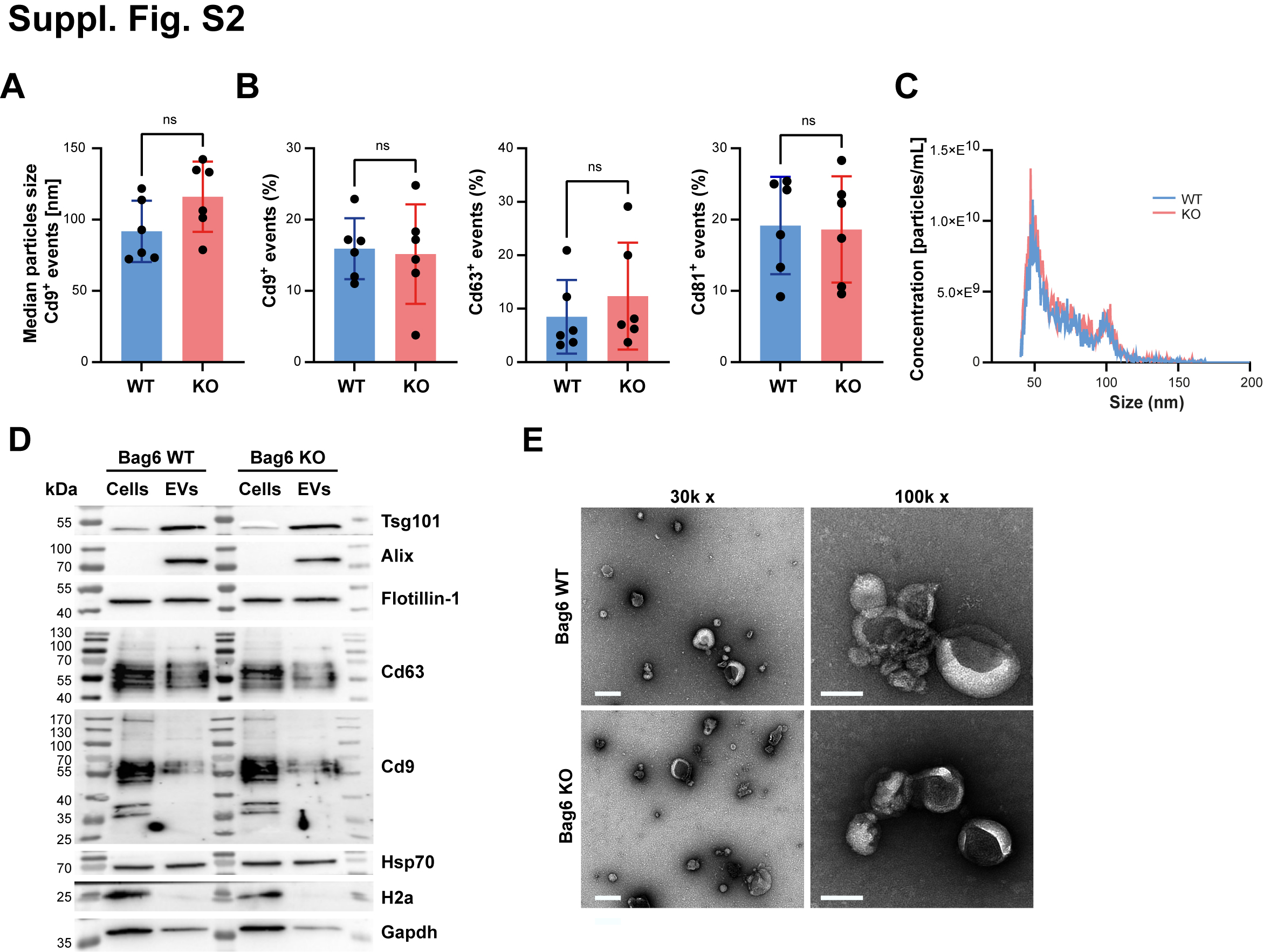

Supplement: Supplementary file 4 — Supplementary figure S2 [file 41423_2024_1195_MOESM4_ESM.jpg]

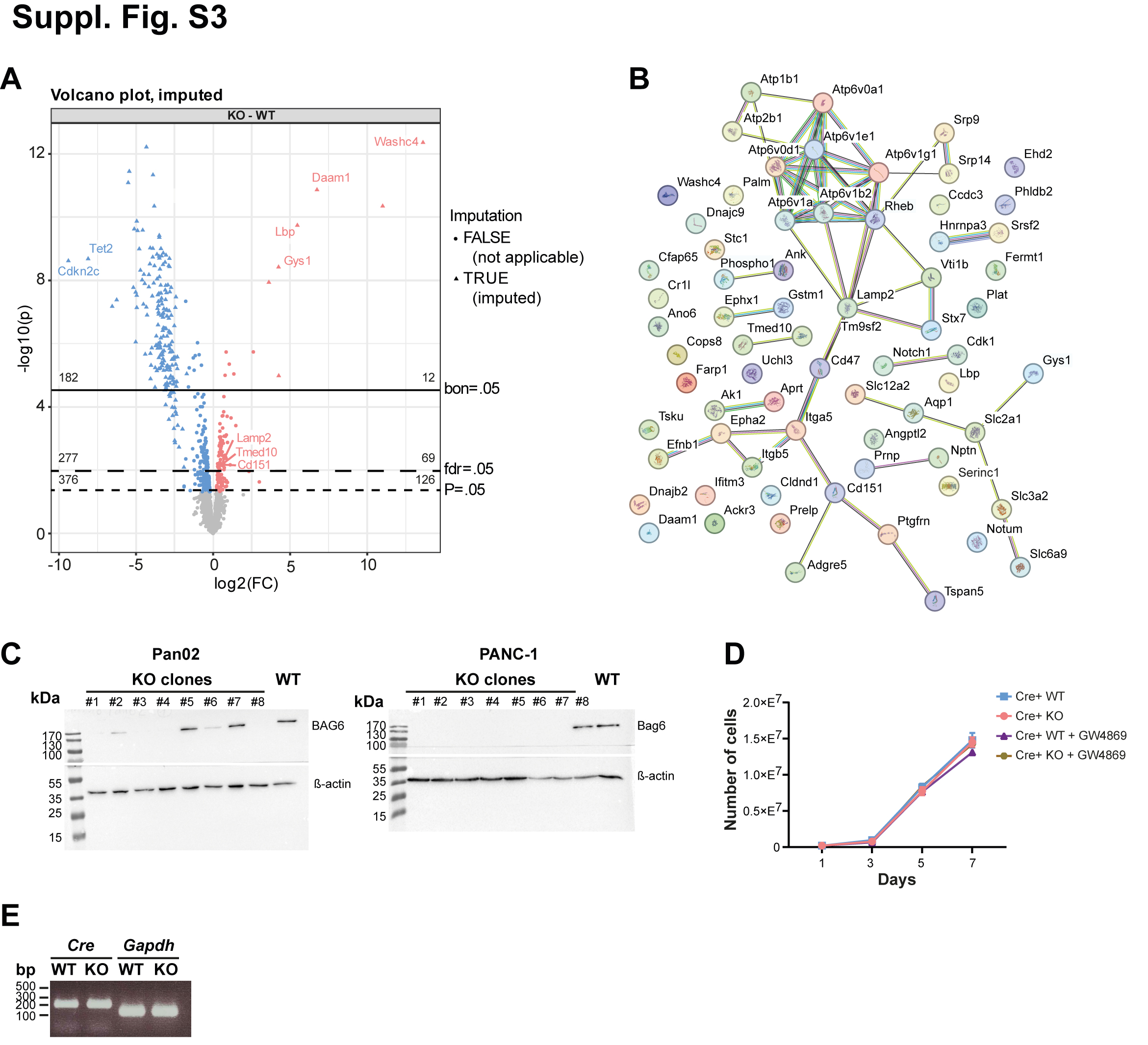

Supplement: Supplementary file 5 — Supplementary figure S3 [file 41423_2024_1195_MOESM5_ESM.jpg]

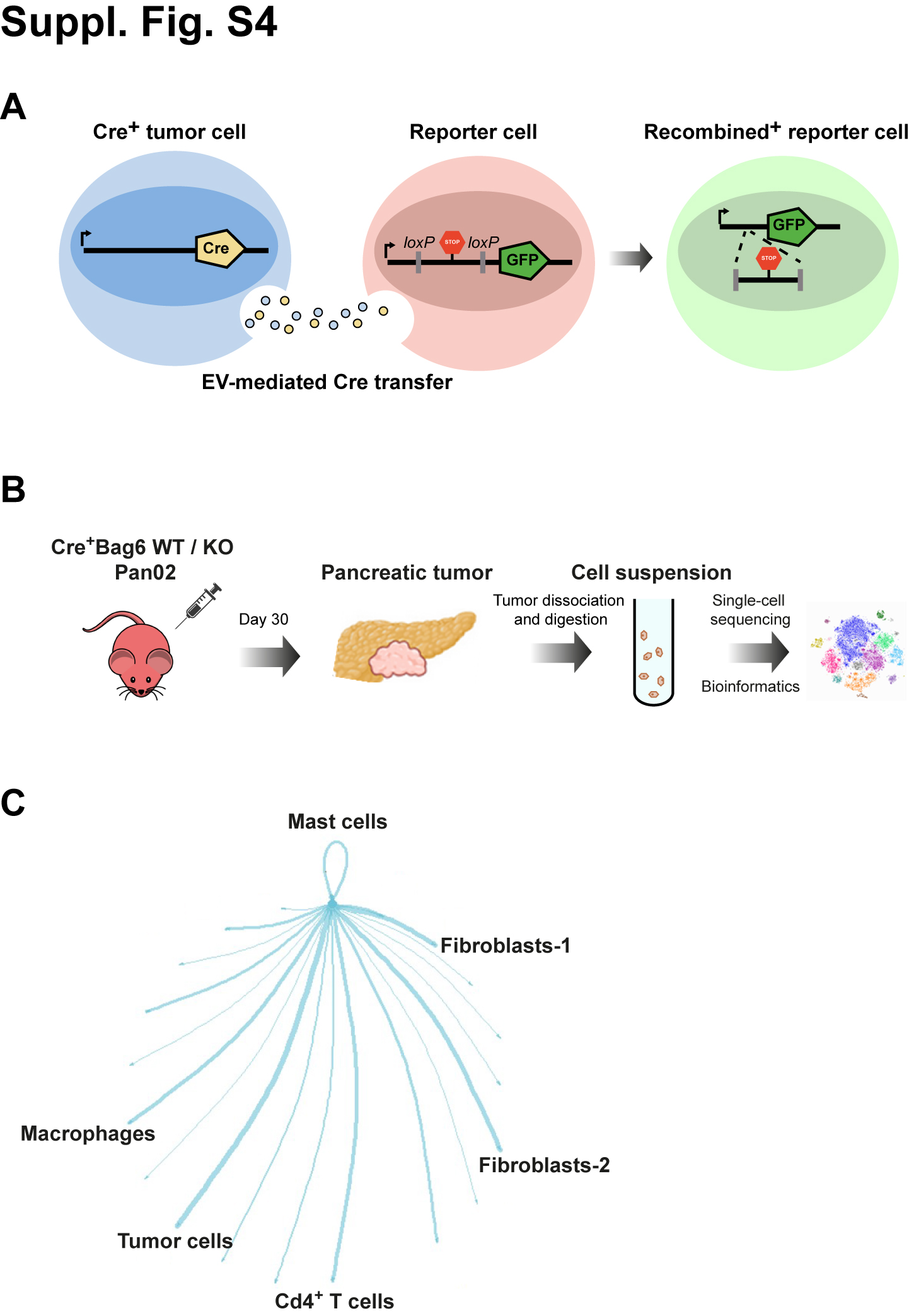

Supplement: Supplementary file 6 — Supplementary figure S4 [file 41423_2024_1195_MOESM6_ESM.jpg]

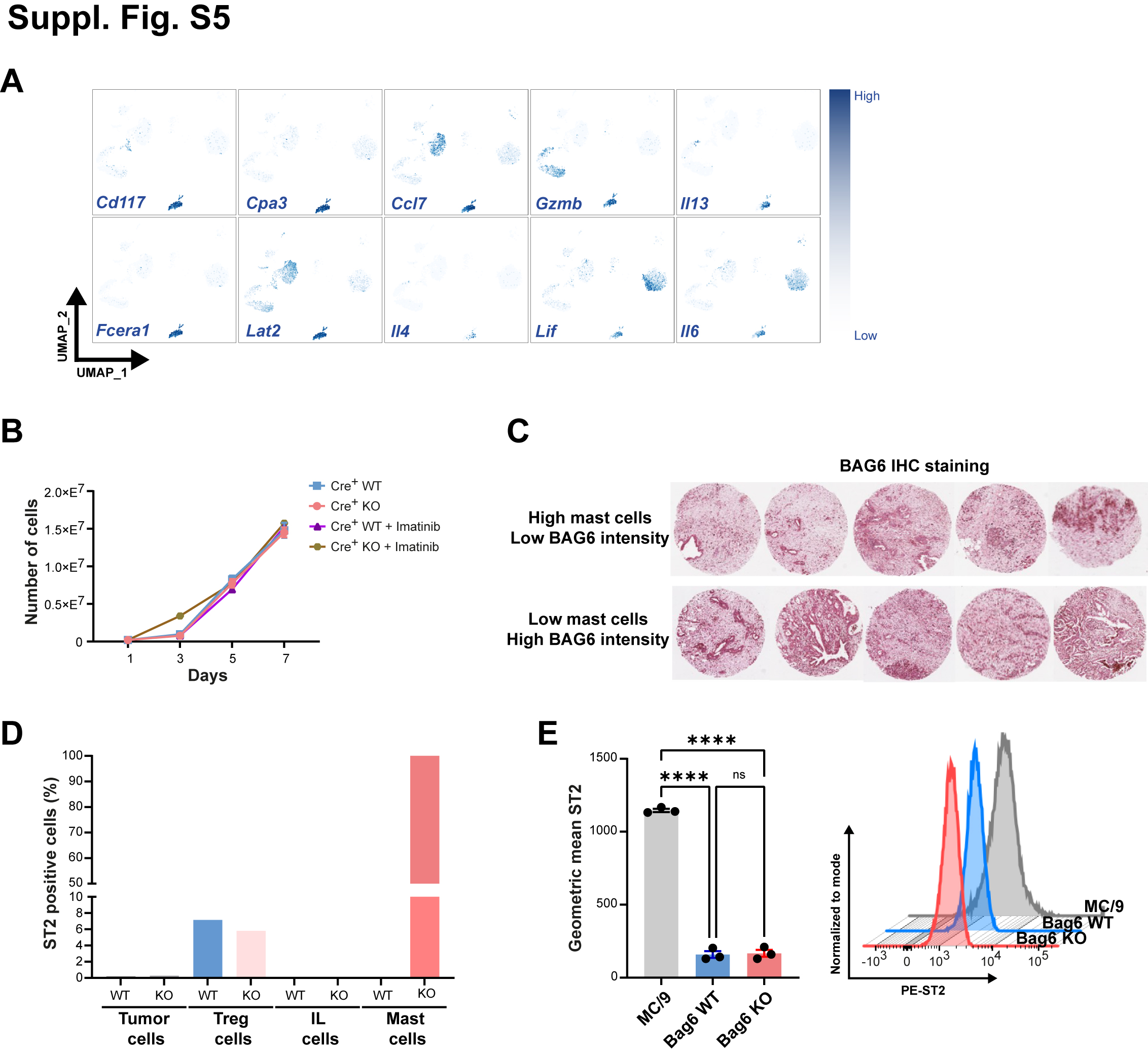

Supplement: Supplementary file 7 — Supplementary figure S5 [file 41423_2024_1195_MOESM7_ESM.jpg]

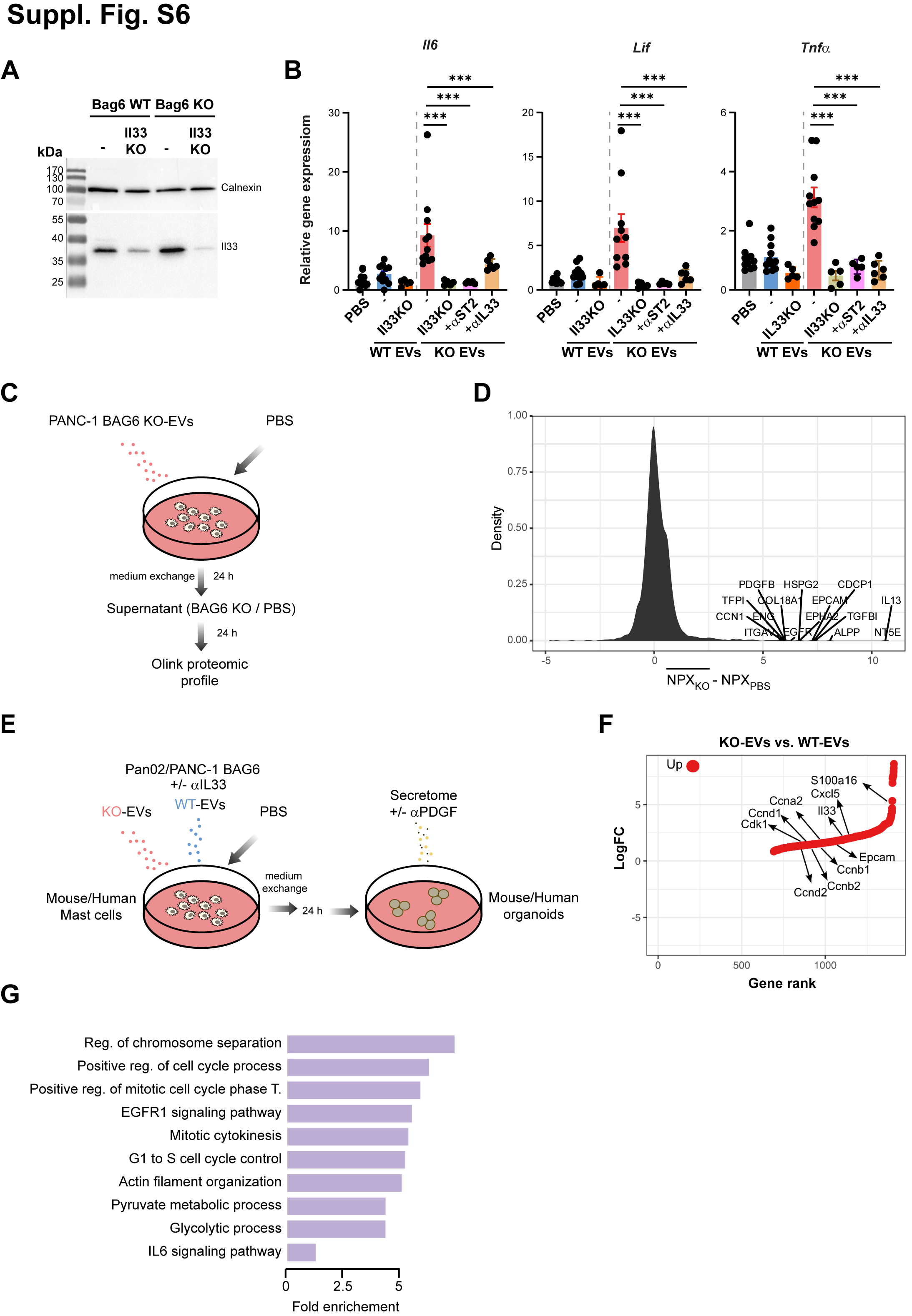

Supplement: Supplementary file 8 — Supplementary figure S6 [file 41423_2024_1195_MOESM8_ESM.jpg]

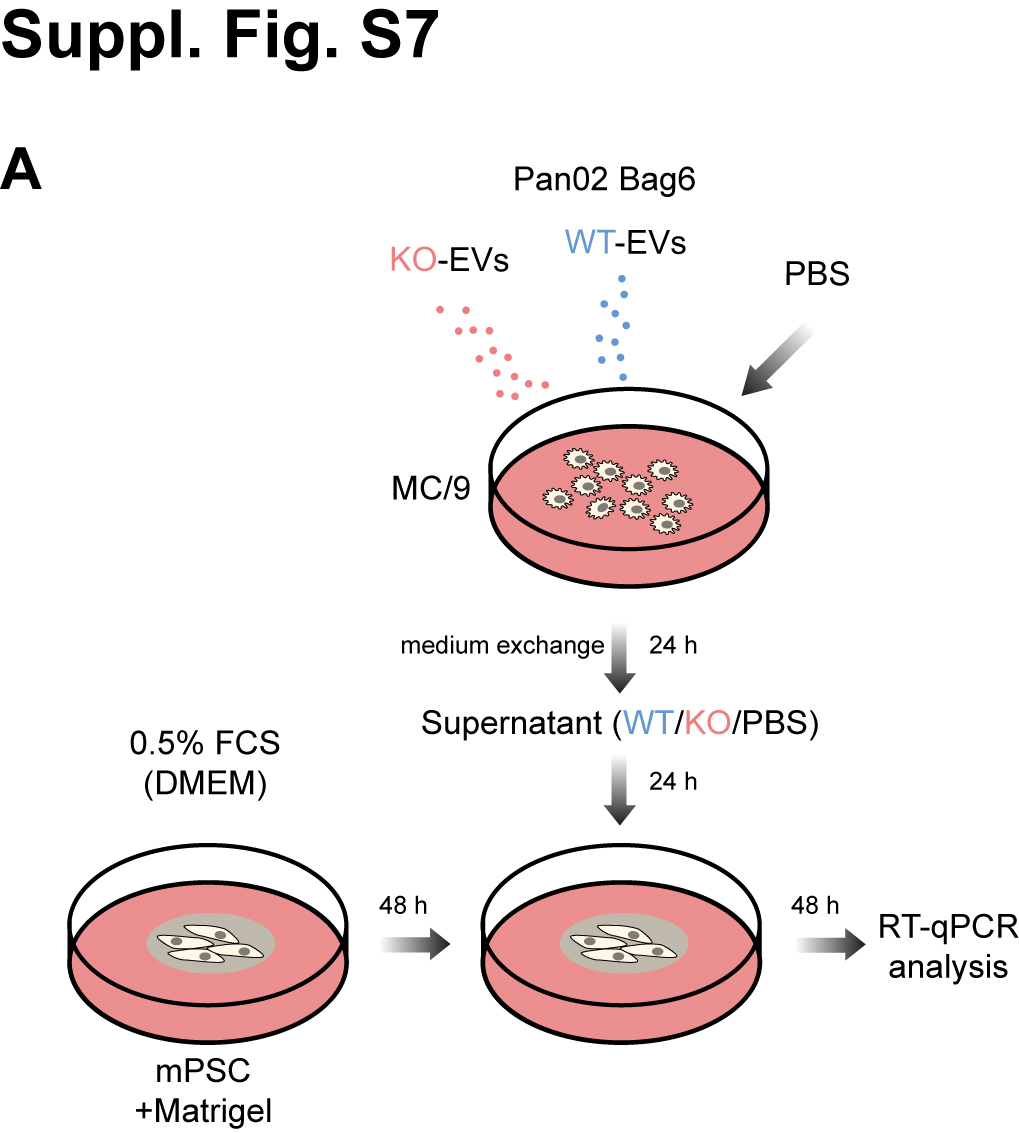

Supplement: Supplementary file 9 — Supplementary figure S7 [file 41423_2024_1195_MOESM9_ESM.jpg]
