# Supplementary material for: BAG6 restricts pancreatic cancer progression by suppressing the release of IL33-presenting extracellular vesicles and the activation of mast cells
Source: Cell Mol Immunol. 2024 Jun 28;21(8):918–31. doi: 10.1038/s41423-024-01195-1 (PMC11291976; doi:10.1038/s41423-024-01195-1)
Supplement: Supplementary file 10 — Table S1 [file 41423_2024_1195_MOESM10_ESM.pdf]

Protein	HMCs BAG6 KO EV_1 (NPX)	HMCs BAG6 KO EV_2 (NPX)	HMCs PBS (NPX)	SD pooled (SD=√(Σ(NPX_i - mean) / 3))	Mean NPX BAG6 KO - NPX PBS
NT5E	4.796	4.8973	-5.9273	3	10.77395
IL13	7.7111	7.8312	-2.8532	6.134265262	10.62435
ALPP	-2.0152	-1.7296	-9.9495	4.665501757	8.0771
TGFBI	1.3896	1.4707	-5.9637	4.269033877	7.39385
CDCP1	4.3988	4.3542	-2.9576	4.23440333	7.3341
EPCAM	1.2407	1.3728	-5.9566	4.194017208	7.26335
EPHA2	1.8772	2.025	-4.7267	3.856137812	6.6778
HSPG2	3.2488	3.4279	-3.3092	3.83900936	6.64755
EGFR	0.7937	0.8508	-5.4406	3.615970844	6.26285
COL18A1	1.4155	1.3847	-4.6256	3.478973602	6.0257
PDGFB	-2.0879	-2.1604	-8.1446	3.476097447	6.02045
ENG	0.0963	0.0838	-5.897	3.45663058	5.98705
TFPI	-0.366	-0.1477	-6.2307	3.450730598	5.97385
CCN1	-1.1446	-1.1807	-7.0807	3.416835437	5.91805
ITGAV	-1.1959	-1.0059	-6.9044	3.351998769	5.8035
THY1	-1.9817	-2.1262	-7.7503	3.289582725	5.69635
CEACAM19	-4.7261	-4.1672	-9.9781	3.205787267	5.53145
SYT1	3.8096	3.7674	-1.5787	3.098826201	5.3672
ANXA2	4.1241	4.1866	-1.1219	3.046981963	5.27725
F3	-1.659	-1.3467	-6.6976	3.003252461	5.19475
EDIL3	2.2404	2.2996	-2.4318	2.714746871	4.7018
SUSD2	-0.7171	-0.8574	-5.3716	2.647705169	4.58435
ANGPTL4	-2.0641	-1.8348	-6.4814	2.619033198	4.53195
CD63	-0.5976	-0.3567	-4.9591	2.590456872	4.48195
CKAP4	-2.6646	-2.321	-6.8683	2.532031198	4.3755
ADAMTS15	0.391	0.3696	-3.9013	2.47200607	4.2816
TNFAIP8	-1.6335	-4.0322	-7.0719	2.725488713	4.23905
EFB4A_DEFB4	-7.1513	-8.8444	-11.9898	2.455307551	3.99195
CST5	-7.8423	-6.7848	-11.2918	2.356919087	3.97825
NRP1	-0.5515	-0.4526	-4.4376	2.272728882	3.93555
ITGB5	-0.324	-0.2982	-4.2344	2.265155044	3.9233
CRIM1	-0.5165	-0.2821	-4.2378	2.219255864	3.8385
TNC	1.4872	1.6412	-2.1913	2.169605744	3.7555
EPHB4	-1.0523	-1.0235	-4.7137	2.122272973	3.6758
ICAM1	3.2236	3.2078	-0.3919	2.082863813	3.6076
VWA1	-3.0153	-2.793	-6.5034	2.080998441	3.59925
ITGA2	-0.646	-0.5388	-4.1839	2.074246139	3.5915
PXN	-1.6891	-1.9261	-5.3745	2.062757245	3.5669
CD109	-0.1334	-0.1096	-3.4077	1.897325773	3.2862
BSG	-1.692	-1.5192	-4.8827	1.894006273	3.2771
ITGA5	0.913	1.0445	-2.2809	1.883108008	3.25965
ANPEP	5.1537	5.2626	2.133	1.776273433	3.07515
LAMA4	-2.0032	-1.8522	-4.9922	1.770900054	3.0645
TINAGL1	-1.9885	-2.1416	-5.0579	1.729617571	2.99285
IST1	0.7549	1.1458	-1.995	1.711693969	2.94535
F7	-7.2242	-7.4007	-10.194	1.66600266	2.88155

CD59	2.4664	2.5286	-0.3817	1.662597794	2.8792
VIM	0.2337	0.5193	-2.3967	1.607463194	2.7732
SERPINE1	0.3341	0.5235	-2.3146	1.586731213	2.7434
SLC16A1	2.3289	2.0778	-0.5294	1.582741395	2.73275
PDGFA	0.351	0.2484	-2.4194	1.570711079	2.7191
SPRR3	-9.7635	-4.9854	-10.0833	2.855436221	2.70885
ITGB1	3.3129	3.4911	0.7508	1.533262086	2.6512
FGFBP1	-3.2283	-3.0807	-5.7784	1.516705919	2.6239
KRT19	0.5725	0.5323	-2.0557	1.505921384	2.6081
ITGB1BP2	-1.0909	-1.121	-3.6807	1.486608788	2.57475
ERBB2	-4.0458	-3.9308	-6.5537	1.482250081	2.5654
LGALS3	-1.6384	-1.5593	-4.1061	1.44810165	2.50725
ACP6	-8.7696	-7.8569	-10.7935	1.502931139	2.48025
MFGE8	7.1249	7.2731	4.7265	1.429420475	2.4725
SPINT2	-1.7935	-1.6948	-4.2165	1.428264774	2.47235
CA2	-4.599	-3.9511	-6.7442	1.461908858	2.46915
KRT18	-1.6203	-1.1923	-3.8016	1.399386781	2.3953
MXRA8	-3.0014	-2.7078	-5.2268	1.377435245	2.3722
MATN2	-3.2925	-3.2925	-5.6434	1.357292748	2.3509
CTRL	-6.3344	-6.7481	-8.8585	1.353761221	2.31725
GLIPR1	-4.2484	-8.6378	-8.7483	2.566714587	2.3052
KLK14	-8.4446	-12.7135	-12.8114	2.493392397	2.23235
ARHGEF5	-1.6513	-1.886	-4	1.293604253	2.23135
CD86	-6.0222	-6.2136	-8.3445	1.289085339	2.2266
OCLN	3.7849	3.7382	1.6196	1.236875832	2.14195
MCAM	-0.6804	-0.3163	-2.6327	1.245642609	2.13435
PODXL	-4.8028	-5.0784	-7.0364	1.217831975	2.0958
MITD1	3.633	3.4668	1.464	1.207158598	2.0859
SERPINA12	-6.1698	-4.5155	-7.4285	1.460970179	2.08585
CA9	-4.4513	-4.3103	-6.387	1.160423657	2.0062
PPL	-0.2231	-0.0536	-2.1283	1.152019776	1.98995
CXADR	-1.8843	-1.8274	-3.8251	1.137302916	1.96925
ENAH	0.8277	1.0845	-0.984	1.12745254	1.9401
LTBP2	-1.7261	-1.8965	-3.7414	1.1175961	1.9301
CDH1	-3.1038	-2.8562	-4.9075	1.119707636	1.9275
TNFRSF10B	-0.3066	-0.5787	-2.3499	1.109524128	1.90725
GCG	-10.7302	-7.4475	-10.9816	1.971851248	1.89275
PDIA5	-0.901	-0.6312	-2.6527	1.097550848	1.8866
AKAP12	1.5792	1.714	-0.2349	1.088373485	1.8815
FAS	0.2867	0.5812	-1.4159	1.078114513	1.84985
KIAA1549	-0.7082	-0.5318	-2.3915	1.02657196	1.7715
CYB5R2	-3.9826	-4.0543	-5.7888	1.022740565	1.77035
LTBP3	-1.9665	-2.3803	-3.939	1.040154827	1.7656
PER3	0.2287	-1.1907	-2.2145	1.226926311	1.7335
MILR1	-1.5864	-1.5842	-3.3176	1.000144476	1.7323
HEG1	-4.3721	-4.262	-6.0474	1.000533629	1.73035
LXN	-1.4603	-1.2563	-3.0773	0.997692839	1.719
LGALS3BP	5.6549	5.6873	3.9674	0.983765048	1.7037
PCSK7	-2.7885	-2.79	-4.4648	0.967379534	1.67555
FUT3_FUT5	-7.6727	-7.4114	-9.2171	0.975875807	1.67505

CA13	-1.1374	-1.2413	-2.8539	0.962431488	1.66455
ADGRG2	-5.4443	-6.2912	-7.4933	1.029618458	1.62555
CHMP6	0.6853	0.7412	-0.9031	0.933618575	1.61635
LYPD8	-4.9406	-2.243	-5.1921	1.634905136	1.6003
TNPO1	0.9577	-0.1097	-1.1415	1.04965031	1.5655
CEP112	0.9254	0.8263	-0.6785	0.898771296	1.55435
SCAMP3	2.3085	2.3815	0.8171	0.882888283	1.5279
MAG	-0.2751	-0.2893	-1.8015	0.877196998	1.5193
TNFAIP2	-1.5304	-1.4198	-2.987	0.87464581	1.5119
YES1	-0.3013	-0.2414	-1.7485	0.853358684	1.47715
BAIAP2	4.1232	3.9935	2.5882	0.851265252	1.47015
EZR	3.4161	3.275	1.8814	0.848266297	1.46415
VAMP8	-0.1937	-0.8034	-1.9444	0.888684944	1.44585
NOTCH2	-1.8939	-1.6622	-3.2172	0.838931143	1.43915
XCL8-OID201	3.7826	3.8466	2.3917	0.822134703	1.4229
PLSCR3	1.9661	2.0808	0.6082	0.819105128	1.41525
SLAMF7	-3.7136	-4.4453	-5.4868	0.891099076	1.40735
DIABLO	2.4519	2.6567	1.1668	0.807591797	1.3875
TAGLN3	-0.1729	-0.4443	-1.696	0.812428916	1.3874
ANXA4	2.6007	2.7303	1.282	0.801388248	1.3835
EPB41L5	1.0301	1.3309	-0.1998	0.810984704	1.3803
WASL	0.3709	1.1076	-0.6317	0.873030941	1.37095
CSPG4	0.0497	0.1702	-1.2472	0.785863922	1.35715
TNFRSF10A	0.12	0.0839	-1.2533	0.782662117	1.35525
XCL8-OID206	1.251	1.3052	-0.0681	0.777701243	1.3462
AK2	0.3796	0.2917	-1.006	0.775847822	1.34165
JUN	1.4534	1.6649	0.2408	0.76846078	1.31835
CD40	1.7408	1.7884	0.4666	0.749778483	1.298
DSG3	-6.5426	-8.4123	-8.7733	1.197366665	1.29585
SNAP23	1.7432	1.8378	0.5	0.746570407	1.2905
STOML2	-0.3974	-0.9396	-1.9526	0.789387619	1.2841
LONP1	3.2112	2.7323	1.6891	0.778288706	1.28265
PADI2	-0.3589	-0.2939	-1.5944	0.73280119	1.268
ATP6V1G2	-1.2013	-1.1831	-2.4525	0.727691446	1.2603
XCL8-OID209	1.2986	1.2398	0.0128	0.725978429	1.2564
VNN1	-5.3348	-5.569	-6.7013	0.730784416	1.2494
LAT2	-1.5923	-1.5602	-2.8076	0.711101406	1.23135
BTLA	2.4695	2.2906	1.152	0.714635271	1.22805
IGFBP1	-5.4469	-5.4213	-6.6606	0.708235782	1.2265
NBN	3.5221	3.6906	2.3863	0.709416706	1.22005
PSIP1	2.4461	2.372	1.1919	0.703697906	1.21715
CA14	-8.1434	-7.9657	-9.2643	0.704078128	1.20975
COL9A2	-0.44	0.2716	-1.2918	0.782747017	1.2076
KYAT1	1.0755	0.8229	-0.2475	0.702364094	1.1967
HDGF	3.2761	3.332	2.1081	0.691047516	1.19595
AGT	-5.3603	-5.3174	-6.5121	0.677715739	1.17325
XCL8-OID214	1.1501	1.0669	-0.0644	0.678450708	1.1729
NUMB	-0.774	-0.7243	-1.9166	0.6744855	1.16745
PPY	-7.1405	-7.0268	-8.2381	0.668942093	1.15445
CD2AP	1.8115	1.6366	0.5715	0.671146782	1.15255

OSCAR	-7.9917	-8.8821	-9.5836	0.79781577	1.1467
PDCD6	-0.6139	-0.5067	-1.6992	0.659725218	1.1389
THBD	-3.1527	-3.2381	-4.33	0.656451834	1.1346
TP53I3	-0.196	-0.1589	-1.3112	0.654833661	1.13375
LAG3	-4.4889	-4.5298	-5.6422	0.654370876	1.13285
TGOLN2	1.1082	1.2107	0.0282	0.655135164	1.13125
RALY	0.6125	0.6637	-0.4843	0.64852341	1.1224
ZCCHC8	4.5762	4.1992	3.2671	0.673879591	1.1206
CEP290	-0.1109	0.1844	-1.0756	0.658969926	1.11235
CD274	-7.0688	-5.3821	-7.3378	1.060037765	1.11235
LAMP1	1.4049	1.5479	0.3668	0.644605541	1.1096
CD302	-6.0338	-7.0156	-7.634	0.806947937	1.1093
CD164	2.8772	2.8786	1.7765	0.635893972	1.1014
PRKAR1A	0.3562	0.2878	-0.7763	0.635025409	1.0983
RBKS	-2.7508	-2.9395	-3.9363	0.637001855	1.09115
FOLR1	-7.9902	-6.8197	-8.4952	0.85949554	1.09025
EFHD1	-1.4071	-1.4818	-2.5299	0.627796881	1.08545
LILRB1	-6.6693	-7.0394	-7.9391	0.653047489	1.08475
CTSL	-0.23	-0.0201	-1.2089	0.63450094	1.08385
CEP20	1.0609	1.7477	0.3262	0.710884494	1.0781
PINLYP	-5.278	-6.9444	-7.1794	1.036615962	1.0682
PTS	-1.2225	-1.1699	-2.2594	0.614401961	1.0632
SEMA4C	-1.4218	-1.8538	-2.7007	0.650570112	1.0629
CD58	-3.1973	-2.8728	-4.0946	0.632882662	1.05955
RGMA	-7.9191	-6.902	-8.4676	0.794402083	1.05705
YJU2	0.7825	0.8442	-0.2431	0.610721369	1.05645
FHIT	-0.2194	-0.5046	-1.4121	0.622820009	1.0501
GUSB	-0.0484	0.0949	-1.0219	0.607656616	1.04515
NLGN1	-0.0956	-0.7813	-1.4821	0.693263704	1.04365
RGCC	-1.2785	-1.7385	-2.5504	0.644012321	1.0419
LATS1	0.4684	0.6741	-0.4698	0.609786703	1.04105
TNFSF12	-8.7814	-9.5174	-10.1777	0.698491921	1.0283
C19orf12	0.2219	0.4237	-0.704	0.60134867	1.0268
TCOF1	3.8283	3.7113	2.7462	0.593864101	1.0236
CSRP3	-0.6444	-1.4677	-2.0713	0.71626339	1.01525
OSMR	-2.1047	-1.8317	-2.9834	0.601810543	1.0152
SORT1	-5.2492	-3.8784	-5.579	0.901840806	1.0152
C9	-3.0006	-3.3319	-4.1742	0.605057289	1.00795
LY75	-5.1849	-6.614	-6.9056	0.920884164	1.00615
PPP1R2	2.855	2.8364	1.8402	0.580600184	1.0055
LPL	-8.2918	-7.3991	-8.8445	0.72933437	0.99905
EIF4G3	-0.5868	-0.0327	-1.3037	0.637235359	0.99395
AKR1B1	-3.005	-2.5368	-3.759	0.616644155	0.9881
GRPEL1	1.4331	1.4292	0.4489	0.567105654	0.98225
MTR	1.0954	1.1684	0.1526	0.566576044	0.9793
PNMA2	0.3265	0.0166	-0.8067	0.585662465	0.97825
STK4	2.1519	2.1224	1.159	0.564927756	0.97815
NIT2	2.1454	1.6976	0.949	0.604469431	0.9725
CCN5	-4.3745	-4.4877	-5.3963	0.560125498	0.9652
AHSP	-5.41	-5.3259	-6.3328	0.558641247	0.96485

NUDT16	1.462	1.5123	0.5244	0.556412656	0.96275
SLC1A4	1.5715	1.2531	0.4503	0.57777675	0.962
QDPR	-2.3896	-2.5377	-3.4254	0.560182491	0.96175
CPM	-4.4095	-4.2414	-5.2834	0.559422741	0.95795
FDX1	3.1177	1.6455	1.4249	0.920290628	0.9567
CD69	1.5809	1.8346	0.7512	0.566645295	0.95655
MDM1	0.2662	0.1009	-0.7723	0.558015021	0.95585
COL28A1	-1.4219	-2.0222	-2.677	0.627747181	0.95495
CD14	-7.277	-7.0577	-8.1192	0.56038314	0.95185
CLPS	-6.74	-6.9232	-7.7821	0.556363769	0.9505
SPINT3	-7.0162	-7.3941	-8.1501	0.577360895	0.94495
SCARB1	0.118	-0.6412	-1.2055	0.664137458	0.9439
KLK6	-10.3222	-8.2773	-10.2423	1.1582476	0.94255
NMNAT1	2.9116	2.7989	1.913	0.546918955	0.94225
EPS8L2	-2.3476	-2.182	-3.2066	0.550016594	0.9418
GOT1	2.2628	2.2182	1.3051	0.540513654	0.9354
TIMM8A	4.0631	3.9592	3.0789	0.540736063	0.93225
PTPRF	-4.0504	-4.4342	-5.1717	0.569871679	0.9294
PRKAR2A	-0.2869	-0.3487	-1.2459	0.536728994	0.9281
LBR	4.6341	4.6773	3.7323	0.53356263	0.9234
LRRC25	-4.9752	-4.8496	-5.834	0.535779208	0.9216
LRP11	-5.644	-5.8071	-6.6468	0.538099362	0.92125
APOE	-0.8053	-0.4444	-1.5441	0.560567323	0.91925
HNMT	-5.6542	-6.7519	-7.1185	0.761961869	0.91545
EIF4B	0.7509	0.5458	-0.2664	0.537995375	0.91475
LAP3	-0.4375	-1.1355	-1.6986	0.631751378	0.9121
SETMAR	-1.6767	-1.71	-2.6037	0.525854476	0.91035
NACC1	2.4146	2.5096	1.5538	0.526554093	0.9083
SIGLEC7	-3.7032	-4.0585	-4.784	0.550865528	0.90315
RBM19	1.8247	1.6562	0.8413	0.525916632	0.89915
PPP1R14A	-0.6869	-1.0529	-1.7625	0.546870417	0.8926
ERMAP	-3.0236	-3.5149	-4.1579	0.56883817	0.88865
NAGPA	-5.2087	-5.0595	-6.0215	0.517743257	0.8874
CHI3L1	-3.6001	-4.1608	-4.7652	0.582686574	0.88475
TPR	4.195	4.1277	3.2769	0.511744979	0.88445
HMOX1	-2.441	-2.4354	-3.3204	0.509346104	0.8822
ADGRE5	3.3336	3.4635	2.5239	0.509139185	0.87465
CIT	5.573	5.2899	4.5569	0.524387551	0.87455
SERPINB8	-0.954	-0.9779	-1.8405	0.505063069	0.87455
NDRG1	-0.6666	-0.9869	-1.7013	0.52971117	0.87455
MGLL	-3.4669	-3.4556	-4.3339	0.503856392	0.87265
CRIB-OID310	1.3928	1.3427	0.504	0.499315054	0.86375
SART1	2.973	2.7564	2.001	0.510282392	0.8637
MYDGF	2.5476	2.4573	1.6388	0.500668516	0.86365
EIF4G1	1.4409	1.6366	0.6752	0.508082169	0.86355
LACTB2	2.8745	2.8717	2.012	0.497158288	0.8611
LRP1	-3.3392	-2.5533	-3.8063	0.633223265	0.86005
STX4	2.1505	2.189	1.3118	0.495711573	0.85795
ACY3	-6.2188	-5.8875	-6.9082	0.520714339	0.85505
UFD1	-1.3838	-1.7134	-2.4036	0.520417179	0.855

AXL	-2.7776	-2.4883	-3.4873	0.514031384	0.85435
DFFA	1.2605	1.3861	0.4705	0.496353154	0.8528
PGD	2.7207	2.6268	1.822	0.493994254	0.85175
ITGB2	-1.737	-1.4886	-2.4633	0.506500138	0.8505
AKR1B10	-1.3206	-0.5268	-1.7736	0.631115108	0.8499
ANXA1	-0.0611	0.1874	-0.7839	0.504581639	0.84705
ENPP7	-7.6529	-7.6195	-8.4826	0.488954541	0.8464
TPD52L2	2.1151	2.2518	1.3391	0.49225403	0.84435
PLIN3	1.7489	1.6008	0.8325	0.491936219	0.84235
EIF4EBP1	4.1331	4.051	3.2508	0.487427557	0.84125
RCC1	2.3266	2.4186	1.5318	0.487610719	0.8408
MTDH	0.6131	0.3286	-0.3695	0.505599746	0.84035
TNFRSF17	-6.0935	-5.4309	-6.6023	0.587380365	0.8401
PSMG3	1.0965	1.348	0.3844	0.499810567	0.83785
PTGR1	-0.4632	-0.4657	-1.3001	0.482464372	0.83565
IL1A	1.6061	0.6495	0.2922	0.679347734	0.8356
SPAG1	-2.3997	-2.3763	-3.2231	0.482287148	0.8351
RNF168	2.3619	2.0705	1.3834	0.502407972	0.8328
GET3	2.2443	2.2843	1.4321	0.480886972	0.8322
CCL5	-2.1829	-2.0954	-2.9705	0.48196992	0.83135
THTPA	-0.7072	-0.7079	-1.5389	0.479980274	0.83135
FABP2	-6.6847	-5.5091	-6.9275	0.75860022	0.8306
BTN2A1	-1.1872	-1.0597	-1.9502	0.481562647	0.82675
OXCT1	-1.1726	-1.0247	-1.9243	0.482391173	0.82565
ELOA	3.2922	3.3541	2.4989	0.476886349	0.82425
PLXNB2	0.6054	0.7189	-0.1615	0.478908833	0.82365
SEC31A	-0.0538	-0.4909	-1.0948	0.52272246	0.82245
SHD	-0.4168	-0.5338	-1.297	0.478001967	0.8217
TP53BP1	1.4499	1.5283	0.6725	0.473091069	0.8166
CDKN1A	0.7131	1.1042	0.0929	0.509956688	0.81575
LYPLA2	-0.9378	-0.3006	-1.4342	0.568255482	0.815
ELOB	0.399	0.4102	-0.4091	0.469823289	0.8137
PIBF1	2.2113	2.2878	1.4361	0.471200626	0.81345
CDA	-4.8692	-6.0562	-6.274	0.756071963	0.8113
IFNL2	-0.0119	-0.3254	-0.9797	0.493799484	0.81105
VSIG2	-1.8356	-1.5924	-2.5243	0.483372103	0.8103
PSMG4	0.2825	0.2499	-0.541	0.466322103	0.8072
COL6A3	-5.6897	-5.6217	-6.462	0.466757499	0.8063
ZFYVE19	-0.7123	-0.4804	-1.3999	0.478200031	0.80355
PMS1	1.3257	1.0587	0.3887	0.482728012	0.8035
SERPINB1	3.1468	3.2573	2.3987	0.467093463	0.80335
GCHFR	-0.7817	-0.5133	-1.4504	0.482589311	0.8029
TRIM24	2.0934	2.0886	1.2894	0.462810199	0.8016
TRIM21	1.3111	1.3625	0.5366	0.462709956	0.8002
CEND1	1.2267	1.2506	0.4439	0.459004709	0.79475
MTUS1	2.043	1.9701	1.2121	0.460121944	0.79445
CDH3	-1.8503	-1.9876	-2.7117	0.462814228	0.79275
SORD	-4.4815	-4.1553	-5.1085	0.484445676	0.7901
PRSS22	-1.9665	-3.6159	-3.5808	0.94231248	0.7896
LEP	-5.3017	-4.6399	-5.7591	0.562702207	0.7883

FOXJ3	1.1366	0.9146	0.2374	0.468409451	0.7882
RNF4	0.3511	0.4268	-0.399	0.456495005	0.78795
AHNAK	-0.9044	-0.8057	-1.6421	0.457075475	0.78705
AIFM1	2.5382	2.3813	1.6774	0.458452008	0.78235
IGSF3	2.8678	2.9104	2.1069	0.452105412	0.7822
FAM3D	-6.2233	-6.2431	-7.0148	0.451365554	0.7816
JPT2	0.5131	0.5108	-0.2695	0.451171833	0.78145
MICB_MICA	-5.0819	-5.615	-6.1264	0.522287568	0.77795
BCL2L15	-3.725	-4.1718	-4.7216	0.499186311	0.7732
MESD	6.9245	6.9429	6.164	0.444481724	0.7697
IL1RN	0.3164	0.422	-0.3998	0.447110918	0.769
PRDX5	-2.1516	-1.7433	-2.7161	0.488485581	0.76865
CDSN	-4.8323	-3.7911	-5.0802	0.684023715	0.7685
SNRPB2	3.4044	3.3301	2.6002	0.444411997	0.76705
OGFR	0.6195	0.7237	-0.0951	0.445709965	0.7667
SDC1	-4.7106	-4.3852	-5.3133	0.470903752	0.7654
GPR15L	-5.7	-2.324	-4.7769	1.744811739	0.7649
ATRAID	-1.5357	-1.4174	-2.2411	0.445358608	0.76455
KLB	-3.4254	-3.0614	-4.0071	0.477007928	0.7637
PDIA2	0.0161	0.1994	-0.6539	0.449188116	0.76165
PPIF	3.2739	3.139	2.4453	0.444596383	0.76115
AMIGO2	-5.0974	-6.189	-6.4016	0.699729348	0.7584
MTHFD2	3.0079	3.0829	2.2879	0.438947605	0.7575
GSTM4	-1.2184	-1.3171	-2.0245	0.439688083	0.75675
HEXIM1	3.4528	3.4272	2.6857	0.435683375	0.7543
FNDC1	-3.6673	-3.8554	-4.5149	0.4451119	0.75355
FABP5	1.6943	1.6741	0.9358	0.432206968	0.7484
CAT	2.307	2.2354	1.5233	0.433281791	0.7479
S100A16	-0.557	-0.4749	-1.2637	0.433660916	0.74775
CHCHD10	0.5532	0.494	-0.2238	0.432525622	0.7474
IKBKG	1.9528	1.9215	1.1909	0.431131778	0.74625
DECR1	-1.3198	-0.8912	-1.8512	0.480916472	0.7457
ERP44	-2.1147	-2.0934	-2.8484	0.429882616	0.74435
SLC9A3R2	0.421	0.4448	-0.3109	0.429597979	0.7438
S100A13	4.2955	4.2717	3.5432	0.427635745	0.7404
TPRKB	0.6014	-0.0651	-0.4695	0.54076927	0.73765
PDP1	-2.3687	-1.398	-2.6202	0.645404728	0.73685
LRRFIP1	1.4165	1.4808	0.7118	0.426633641	0.73685
PRKRA	0.1503	-0.2385	-0.7808	0.467654064	0.7367
LETM1	1.4663	1.4774	0.738	0.423724844	0.73385
HSPB1	3.0006	3.2296	2.3818	0.438580909	0.7333
CD55	6.0629	6.3445	5.4711	0.445786451	0.7326
CXCL5	-5.3012	-5.1879	-5.9761	0.426142805	0.73155
PLAU	3.3007	3.4325	2.6368	0.426472535	0.7298
TEX101	-1.3154	-1.8927	-2.3327	0.510191889	0.72865
TBCB	0.7204	0.5094	-0.1137	0.433685258	0.7286
OBP2B	-7.6632	-10.3261	-9.7228	1.396242545	0.72815
NCS1	-0.7071	-0.7804	-1.4666	0.418943827	0.72285
ACY1	-0.1594	-0.1921	-0.8963	0.416330906	0.72055
SHH	0.1784	0.7489	-0.2516	0.501891506	0.71525

FEN1	0.3067	0.5057	-0.308	0.424178575	0.7142
ERP29	4.5874	4.3968	3.7783	0.422988065	0.7138
GSTP1	2.7557	2.6477	1.9881	0.415520942	0.7136
VEGFC	-3.704	-3.3111	-4.2195	0.455576781	0.71195
FDX2	1.5663	1.4118	0.7815	0.415744188	0.70755
PALM	-3.4661	-3.222	-4.0511	0.426070538	0.70705
ENTPD2	-0.3199	-0.2736	-1.0037	0.40881376	0.70695
AMOTL2	-0.2996	0.0108	-0.8506	0.436264278	0.7062
MDGA1	-8.9761	-8.4629	-9.4256	0.481701114	0.7061
ASPSCR1	0.5448	-0.0533	-0.458	0.504498685	0.70375
KRT17	1.3459	0.2955	0.1172	0.663932067	0.7035
TARM1	-1.6413	-0.8417	-1.9438	0.569428196	0.7023
TBCA	3.284	3.209	2.5452	0.406628594	0.7013
CCAR2	0.5669	0.5588	-0.1369	0.40402115	0.69975
FOXO3	1.0483	0.6882	0.1688	0.442147905	0.69945
ACADSB	0.4899	0.0503	-0.4291	0.459643616	0.6992
MNAT1	1.8578	1.7229	1.0914	0.409137145	0.69895
RAB33A	0.5283	0.3	-0.2844	0.419151035	0.69855
PEPD	1.3563	1.4694	0.7168	0.405823981	0.69605
TMCO5A	-1.1503	-0.4515	-1.4965	0.532322095	0.6956
THOP1	1.0623	1.1058	0.3886	0.402106905	0.69545
F11R	5.0651	5.1849	4.4297	0.405876057	0.6953
GMPR2	2.7692	2.7188	2.0489	0.40210659	0.6951
FKBP5	-0.4702	-0.4429	-1.1512	0.401288587	0.69465
IGBP1	0.2468	-0.0438	-0.5923	0.426104334	0.6938
TPK1	-2.1852	-2.1994	-2.8857	0.400397632	0.6934
PEBP1	5.3069	5.4722	4.6962	0.408749022	0.69335
CENPF	3.2472	3.3683	2.6145	0.404802203	0.69325
DBNL	1.7431	1.7921	1.0753	0.400449764	0.6923
NAA80	-0.9033	-0.5751	-1.4308	0.431700896	0.6916
NMI	0.8708	0.7386	0.1152	0.40353351	0.6895
L1CAM	-3.065	-2.9254	-3.6843	0.403928595	0.6891
PSCA	-6.0512	-5.5664	-6.4979	0.465879845	0.6891
CD200R1	-9.9198	-7.7639	-9.5295	1.148737152	0.68765
IGSF8	1.3166	1.5624	0.7552	0.413755048	0.6843
CD46	4.8432	5.1735	4.325	0.427703476	0.68335
IFNGR1	-1.6342	-1.239	-2.1199	0.441224119	0.6833
CLEC6A	-2.3683	-1.9137	-2.8236	0.454950045	0.6826
IL17A	-0.6718	-0.4885	-1.2622	0.404306554	0.68205
ECHS1	-0.6371	-0.7937	-1.3969	0.401179478	0.6815
NAGK	-1.4343	-1.2706	-2.0337	0.401746118	0.68125
BLMH	1.3718	1.2836	0.6465	0.395755762	0.6812
STAMBP	0.3686	0.2477	-0.3711	0.396796829	0.67925
CNPY4	-1.2431	-1.9225	-2.2618	0.518725769	0.679
AP3B1	0.6093	1.0592	0.1571	0.451050489	0.67715
STAT5B	-1.5369	-1.5861	-2.2374	0.391005665	0.6759
GATD3	-1.8219	-1.1277	-2.1507	0.522263037	0.6759
THAP12	1.253	1.3144	0.6081	0.391264118	0.6756
SLC44A4	-0.5473	-0.6726	-1.2846	0.394515606	0.67465
TOP2B	3.4127	3.4783	2.771	0.390801642	0.6745

TAB2	1.4079	1.2971	0.6795	0.392486297	0.673
SH3BGRL2	0.3563	0.0902	-0.4491	0.410350025	0.67235
NECTIN1	-0.7037	-1.1091	-1.5787	0.437892361	0.6723
CCL4	3.9717	4.1147	3.371	0.394626232	0.6722
S100A4	2.4251	2.4349	1.7594	0.387202096	0.6706
MANF	2.3251	2.4303	1.7079	0.390270129	0.6698
RAB10	2.9255	2.6833	2.1347	0.405172276	0.6697
CD70	-3.9853	-3.5743	-4.4494	0.437818421	0.6696
AZI2	1.3891	0.9038	0.4779	0.45592257	0.66855
ACTN4	2.1405	2.0984	1.4522	0.385811642	0.66725
LILRA4	-0.6799	-0.6625	-1.3377	0.384902291	0.6665
MDH1	7.5192	7.5701	6.8783	0.385558223	0.66635
TXNDC5	2.9797	2.9227	2.2855	0.385397301	0.6657
NIT1	-1.413	-1.7294	-2.2368	0.415573932	0.6656
P4HB	0.3919	0.5354	-0.2019	0.390896828	0.66555
PLA2G4A	-0.2829	-0.3382	-0.9754	0.384845896	0.66485
LMNB2	1.7805	1.7727	1.1122	0.383611344	0.6644
MCEMP1	1.1759	1.1993	0.5239	0.383365952	0.6637
HAVCR2	-3.8756	-3.3651	-4.2838	0.460298305	0.66345
NHLRC3	-1.2803	-1.1109	-1.8585	0.391985833	0.6629
IL1B	2.4954	2.4466	1.8089	0.383041551	0.6621
L3HYPDH	1.6547	1.26	0.7953	0.430174875	0.66205
SPRY2	0.5231	0.7116	-0.0422	0.392281952	0.65955
PHLDB1	0.2186	0.5832	-0.2586	0.422153258	0.6595
CALCOCO2	3.359	3.3603	2.7002	0.38073419	0.65945
AKR7L	-1.5327	-1.5112	-2.1803	0.380250536	0.65835
DNPEP	-1.799	-1.7374	-2.4262	0.381142983	0.658
PPP1R9B	0.1598	0.5522	-0.3019	0.427518315	0.6579
DCTD	2.5353	2.737	1.9788	0.392692072	0.65735
IL16	-3.0181	-3.2238	-3.7781	0.393099008	0.65715
LYAR	2.628	2.9105	2.1134	0.404142677	0.65585
SNU13	1.1669	1.1587	0.507	0.378648505	0.6558
PARP1	2.2825	2.3304	1.6527	0.378201829	0.65375
TBL1X	2.0193	2.284	1.498	0.399919946	0.65365
ADGRE2	0.0078	0.1074	-0.5951	0.380112882	0.6527
CALB2	5.5991	5.4745	4.8842	0.381894676	0.6526
HHEX	0.346	0.2803	-0.3389	0.37789177	0.65205
EPHA10	-1.5916	-1.9391	-2.4172	0.414518037	0.65185
DPY30	2.0749	2.2078	1.4901	0.381826038	0.65125
LILRB2	-5.9496	-6.0973	-6.6745	0.383069867	0.65105
RRM2B	0.4363	0.3043	-0.2803	0.38137836	0.6506
VSIR	-1.7389	-1.4144	-2.2266	0.408823593	0.64995
UROD	1.3096	1.5174	0.7645	0.388838711	0.649
CERT	2.5228	2.1495	1.6874	0.418485854	0.64875
YTHDF3	0.5445	0.2478	-0.251	0.402005925	0.64715
RP2	0.3603	0.4036	-0.2651	0.374201314	0.64705
ENTR1	0.0732	0.0242	-0.593	0.371294869	0.6417
DCTN2	1.3933	0.9087	0.5096	0.442538823	0.6414
PROK1	-3.8324	-3.5536	-4.3342	0.395573221	0.6412
DCTPP1	4.0553	4.0827	3.4282	0.370219624	0.6408

CRKL	1.7828	1.8617	1.1818	0.371862479	0.64045
SNAP29	1.8762	1.9288	1.2621	0.370669318	0.6404
DDC	0.1473	0.1532	-0.4886	0.368852016	0.63885
CRYBB1	3.7807	3.7464	3.1248	0.369181044	0.63875
MVK	-1.0559	-0.8502	-1.5913	0.382577848	0.63825
ZBTB17	0.759	0.6685	0.0758	0.371089787	0.63795
ENO2	2.4383	2.3606	1.7623	0.369904533	0.63715
OPTC	-1.9779	-1.7101	-2.4796	0.390629829	0.6356
PRDX1	2.1564	2.0137	1.4503	0.373354179	0.63475
PDIA4	5.297	5.4684	4.7483	0.376163453	0.6344
TGM2	-1.086	-1.2692	-1.8089	0.375815278	0.6313
CD22	-4.0596	-3.9202	-4.6204	0.370632127	0.6305
MPIG6B	-5.3698	-4.0857	-5.3575	0.737850407	0.62975
PTPRB	-5.0226	-5.6047	-5.9415	0.464874868	0.62785
SUMF2	0.0787	0.1745	-0.5005	0.36521119	0.6271
ADGRG1	-5.5023	-4.5091	-5.6326	0.614502045	0.6269
ACRV1	-1.4458	-1.5996	-2.1495	0.369963543	0.6268
CASP8	0.1277	0.2745	-0.4254	0.369082163	0.6265
STIP1	6.6764	6.711	6.069	0.361085382	0.6247
SRC	-0.8273	-0.6499	-1.3609	0.370072227	0.6223
IGF2R	-0.2917	-0.2975	-0.9167	0.35918131	0.6221
GLRX	0.4893	0.6095	-0.0724	0.36399234	0.6218
SGSH	-1.6603	-1.6868	-2.2945	0.358750419	0.62095
POLR2F	3.4644	3.3986	2.8107	0.359925858	0.6208
RIDA	-1.981	-1.8965	-2.5578	0.359897249	0.61905
FBP1	1.9413	1.6269	1.1658	0.390055727	0.6183
ASAH2	-6.6859	-6.5432	-7.2328	0.364008026	0.61825
PFDN6	-1.7335	-1.3864	-2.1778	0.396693597	0.61785
CD79B	-8.8715	-8.8876	-9.4972	0.356691244	0.61765
ZPR1	0.5956	0.6359	-0.0012	0.356765735	0.61695
LGALS1	3.9138	3.7604	3.2205	0.364163072	0.6166
PAFAH2	-0.2876	-0.5412	-1.0305	0.3776303	0.6161
SCLY	-4.5307	-4.387	-5.0746	0.36269148	0.61575
NUDT5	6.2273	6.3266	5.6631	0.357867382	0.61385
TWF2	5.3301	5.6744	4.8893	0.393537194	0.61295
C9orf40	-1.4794	-1.0316	-1.8681	0.418597814	0.6126
FADD	2.107	2.2928	1.5885	0.365011959	0.6114
CHMP1A	1.9885	1.7391	1.2526	0.374261811	0.6112
TRIM5	1.4982	1.7557	1.0161	0.375440812	0.61085
CRELD2	-0.8047	-0.7347	-1.3802	0.354205802	0.6105
MAPK9	0.7773	1.0021	0.2802	0.369410129	0.6095
IGFBP6	-5.0103	-4.7587	-5.4936	0.373487943	0.6091
MYCBP2	1.55	1.5408	0.9366	0.351520943	0.6088
SSC5D	0.249	0.5361	-0.2152	0.379112943	0.60775
RBM17	1.754	1.5251	1.0324	0.368749025	0.60715
PON1	-4.4105	-4.8822	-5.2523	0.421920636	0.60595
S100A11	1.9931	1.9476	1.3646	0.35046909	0.60575
PSMD9	0.0968	-0.0303	-0.5714	0.354832087	0.60465
HTRA2	-0.1373	0.0311	-0.657	0.358684694	0.6039
KIFBP	1.1023	1.5295	0.7126	0.408593429	0.6033

ITIH5	-0.3804	-0.7349	-1.1608	0.390743995	0.60315
GTPBP2	0.5001	0.5604	-0.0726	0.349359027	0.60285
ARID4B	2.0121	1.9139	1.3612	0.350901539	0.6018
NARS1	3.327	3.2216	2.673	0.351137941	0.6013
TXNRD1	5.2943	5.3599	4.7265	0.348304407	0.6006
CREB3	-0.9003	-0.2299	-1.1638	0.481497252	0.5987
CACNB3	0.7001	0.1736	-0.1611	0.434145095	0.59795
TNFRSF11A	-5.1545	-5.2003	-5.7753	0.345956471	0.5979
LRPAP1	1.5067	1.597	0.954	0.348109269	0.59785
AHCY	5.1895	5.0891	4.5419	0.348543101	0.5974
DTYMK	2.7094	2.8637	2.1911	0.352333682	0.59545
ACYP1	2.5577	2.7513	2.0591	0.357123639	0.5954
QPCT	-5.6021	-5.5984	-6.1956	0.343730461	0.59535
ARL13B	0.1056	0.3735	-0.3557	0.36884973	0.59525
GSTT2B	-4.7593	-4.4802	-5.2142	0.3704922	0.59445
FAM3B	-7.667	-6.7475	-7.8011	0.573517803	0.59385
VPS4B	0.4349	0.4006	-0.176	0.343230452	0.59375
CORO1A	8.9597	9.0509	8.4118	0.345678237	0.5935
DNAJA2	1.9364	2.1979	1.474	0.366566779	0.59315
FGL1	-4.264	-4.7464	-5.0977	0.418564439	0.5925
ACOT13	-0.0079	-0.6218	-0.9073	0.459583837	0.59245
ENPP6	-6.3917	-6.9386	-7.2569	0.437604376	0.59175
TNFRSF9	-3.2314	-3.6799	-4.0459	0.407945768	0.59025
PRTFDC1	-2.7599	-1.118	-2.529	0.888826138	0.59005
SIRT1	1.1048	1.0747	0.5022	0.339555842	0.58755
PDCD5	4.049	3.962	3.4183	0.341799449	0.5872
CYTH3	0.7259	1.3714	0.4615	0.468062606	0.58715
GGT1	-0.3787	-0.142	-0.8474	0.359002261	0.58705
PLPBP	2.256	2.4317	1.757	0.350021804	0.58685
SUOX	0.1938	0.0554	-0.4616	0.345444815	0.5862
RNF43	-1.9015	-1.9719	-2.5229	0.340268306	0.5862
SCARB2	-4.8774	-4.773	-5.4109	0.342159324	0.5857
GSR	5.1581	5.2671	4.6271	0.342403758	0.5855
DYNLT1	1.3976	1.4857	0.8569	0.340467213	0.58475
ING1	3.1102	2.706	2.3238	0.393251285	0.5843
CRYZL1	1.5303	1.4574	0.9127	0.337501175	0.58115
SYAP1	1.2747	1.3946	0.7543	0.340386119	0.58035
ADA	-0.9377	-0.9014	-1.4997	0.335441147	0.58015
BATF	-0.7288	-0.3543	-1.1196	0.38267893	0.57805
BIRC2	-1.0599	-0.6506	-1.4317	0.3907	0.57645
PIK3AP1	-1.6924	-1.1475	-1.9961	0.429975127	0.57615
IMMT	1.2414	1.2731	0.6816	0.332729415	0.57565
TCN2	-0.536	-0.4945	-1.0905	0.332768313	0.57525
GGACT	2.9537	2.9545	2.379	0.332034381	0.5751
METAP2	0.4335	0.4347	-0.1399	0.331399598	0.574
CCL14	-7.9379	-7.5705	-8.3253	0.377444159	0.5711
IL4R	-1.8871	-1.9322	-2.4799	0.330005338	0.57025
PAXX	1.8022	1.9873	1.3252	0.341605869	0.56955
ATP5F1D	-0.1378	-0.482	-0.8791	0.37096445	0.5692
CD93	-7.1238	-6.392	-7.3257	0.49127225	0.5678

CLMP	-6.5861	-6.9195	-7.3206	0.367769634	0.5678
FARSA	0.0087	0.1792	-0.4737	0.338639046	0.56765
TLR3	-3.5168	-3.463	-4.0573	0.328691137	0.5674
ESYT2	1.4476	1.4283	0.871	0.32747095	0.56695
AHNAK2	-0.3994	-0.718	-1.1244	0.363384993	0.5657
SRPX	-1.7506	-1.9084	-2.395	0.335889803	0.5655
LCP1	4.7551	4.7174	4.1719	0.32637243	0.56435
HNRNPUL1	0.7682	0.9434	0.2929	0.336589607	0.5629
STX6	1.4313	1.3423	0.8241	0.327908544	0.5627
CPA4	-4.2282	-4.3372	-4.8451	0.329243836	0.5624
NAMPT	-0.3859	-0.0772	-0.7939	0.359494687	0.56235
ITGB1BP1	0.3715	0.4245	-0.1641	0.325608743	0.5621
ACOX1	1.0253	0.5117	0.2066	0.413751266	0.5619
GBA	0.3532	-0.1972	-0.483	0.425020047	0.561
SH2B3	-2.6252	-2.218	-2.9823	0.382423574	0.5607
SMPDL3A	-5.0943	-5.2652	-5.7403	0.334724469	0.56055
ARL2BP	2.2649	1.7422	1.4435	0.415759333	0.56005
KLK15	-2.0249	-2.4287	-2.7862	0.38088458	0.5594
GGCT	2.2493	2.2552	1.6938	0.322434753	0.55845
ARSA	0.0603	0.1455	-0.455	0.324908546	0.5579
LHPP	3.0197	3.0004	2.4523	0.322161673	0.55775
PPCDC	0.7513	0.7869	0.2115	0.322422228	0.5576
HMMR	1.3257	1.7371	0.974	0.381939013	0.5574
EXOSC10	0.5139	0.3165	-0.1421	0.336555275	0.5573
ANXA11	-2.4208	-2.4317	-2.9833	0.321659142	0.55705
NUB1	-0.1715	0.0373	-0.6227	0.337337339	0.5556
TANK	0.8849	1.2574	0.5163	0.37055171	0.55485
LYN	2.893	2.7839	2.2843	0.324555763	0.55415
EPPK1	-0.89	-0.9697	-1.483	0.321837946	0.55315
LRP2	-1.9914	-0.8421	-1.969	0.657177787	0.55225
TOR1AIP1	-0.5223	-0.8012	-1.2136	0.347791762	0.55185
GLOD4	1.4441	1.5677	0.9544	0.324350618	0.5515
RNF41	1.1372	1.1586	0.5972	0.318126788	0.5507
ROR1	-4.1469	-3.9066	-4.5774	0.339864429	0.55065
SHMT1	2.004	2.2159	1.5598	0.334833904	0.55015
WARS	4.4125	4.4768	3.8956	0.318620344	0.54905
IL2RA	-5.6267	-5.8754	-6.2986	0.339705642	0.54755
ENO1	3.1418	3.1304	2.5887	0.316092935	0.5474
TSNAX	1.0725	0.8602	0.4193	0.333200225	0.54705
HARS1	5.6512	5.7277	5.1442	0.317115515	0.54525
FAM171B	-1.3925	-1.5584	-2.0207	0.325545527	0.54525
SIGLEC8	1.2478	1.0698	0.6136	0.327111622	0.5452
SV2A	-0.2515	-0.7183	-1.029	0.391352991	0.5441
NT5C	2.1831	2.0621	1.5792	0.31951229	0.5434
FUCA1	0.3882	0.3175	-0.1882	0.314369194	0.54105
CSTB	4.6019	4.5091	4.0151	0.315431472	0.5404
GFAP	-2.2964	-1.7376	-2.557	0.418645865	0.54
EEF1D	5.178	5.2392	4.6702	0.312347904	0.5384
STX7	-2.3119	-2.451	-2.9198	0.31850292	0.53835
PYY	-1.9052	-3.0332	-3.0064	0.64365411	0.5372

NID2	-2.2018	-2.4668	-2.8714	0.337216627	0.5371
FUS	0.8304	0.7773	0.267	0.311085535	0.53685
DKK3	-5.0011	-4.9546	-5.5137	0.310245553	0.53585
SERPINB6	-0.0886	-0.2379	-0.6984	0.317859188	0.53515
ABCA2	-1.6511	-2.7078	-2.7136	0.611767219	0.53415
DNM1	-3.6923	-3.8251	-4.2917	0.314809932	0.533
BANK1	-2.0392	-2.2162	-2.6606	0.320145363	0.5329
RFC4	1.0317	1.0746	0.5203	0.308387975	0.53285
XIAP	1.3185	1.3616	0.8075	0.308222166	0.53255
GAPDH	-1.7347	-1.6857	-2.2421	0.308068369	0.5319
IL5	-0.346	-1.1143	-1.2609	0.49139571	0.53075
VNN2	-4.69	-4.5519	-5.1516	0.314055797	0.53065
HSPA2	-0.6032	-0.6165	-1.1402	0.306269919	0.53035
CD300E	-6.7629	-6.9988	-7.4105	0.327752818	0.52965
ALCAM	-4.4986	-4.568	-5.0626	0.307555285	0.5293
RILP	-0.5276	-0.3649	-0.9753	0.31609459	0.52905
SCGB2A2	0.3668	0.2597	-0.2156	0.309992005	0.52885
RNF31	-0.2081	-0.5294	-0.8975	0.34496465	0.52875
MECR	0.1588	-0.2811	-0.5882	0.375462253	0.52705
CASP3	5.9403	5.911	5.4024	0.302453539	0.52325
HMOX2	1.1683	1.3237	0.7239	0.311287798	0.5221
APOBR	-0.5414	-0.6044	-1.0939	0.302444347	0.521
MORF4L1	1.2383	1.6466	0.9215	0.363510921	0.52095
DNPH1	1.4802	1.6179	1.0286	0.308268914	0.52045
SWAP70	-0.2386	-0.2048	-0.7405	0.300005706	0.5188
CRIB-OID3055	1.4688	1.2002	0.8164	0.327890774	0.5181
CA1	-6.7837	-6.4074	-7.1132	0.353158505	0.51765
MARS1	5.0532	5.2211	4.6197	0.310320968	0.51745
MYL6B	-1.9053	-2.2732	-2.6059	0.350447347	0.51665
UPK3BL1	-1.0249	-0.5068	-1.2823	0.394985785	0.51645
TPT1	0.7339	-0.3101	-0.3045	0.601143621	0.5164
HK2	6.1624	6.0281	5.5808	0.304513913	0.51445
EFCAB2	1.4743	1.481	0.9635	0.296863543	0.51415
PDLIM5	-0.7091	-0.55	-1.1425	0.306649806	0.51295
PQBP1	2.2804	2.1603	1.7078	0.301952264	0.51255
LTB	-1.9883	-1.2618	-2.137	0.468310613	0.51195
SAMD9L	0.8951	0.8525	0.3619	0.296312155	0.5119
ARAF	0.3394	0.6569	-0.0137	0.335457454	0.51185
HYOU1	0.4065	0.8239	0.1035	0.361710732	0.5117
AGBL2	-1.2139	-1.7083	-1.9721	0.384900212	0.511
SCT	-0.7849	-1.1843	-1.4954	0.35616331	0.5108
NADK	2.6498	2.6776	2.153	0.295180239	0.5107
PAEP	-5.4107	-5.6867	-6.0579	0.324764859	0.5092
NFKBIE	1.2815	1.3164	0.7919	0.293265073	0.50705
ASAH1	2.0415	2.1147	1.5725	0.294193836	0.5056
RRAS	0.1704	-0.2144	-0.5276	0.349611518	0.5056
DOK2	2.4314	2.2792	1.8498	0.301608952	0.5055
NUDT2	-0.2933	0.2517	-0.5257	0.399037492	0.5049
DTX2	-0.8617	-1.4631	-1.6671	0.41872169	0.5047
CRIB-OID3022	1.0979	1.0837	0.5878	0.290493964	0.503

CNP	-1.0435	-0.794	-1.42	0.315139784	0.50125
FCN1	-4.1684	-4.9212	-5.0455	0.474598557	0.5007
TIGIT	-0.7164	-1.1539	-1.4358	0.362493729	0.50065
NPTN	-2.7454	-3.0781	-3.4119	0.333250151	0.50015
DDX1	1.4303	1.0543	0.7431	0.34410882	0.4992
FUT8	-5.1902	-4.8873	-5.5377	0.325454764	0.49895
RAB39B	-0.8109	-1.0968	-1.4525	0.321432175	0.49865
PARK7	5.0044	5.0312	4.5203	0.287544159	0.4975
ADAM8	-7.5157	-6.1421	-7.3231	0.743710598	0.4942
ERBB4	-6.7486	-6.8032	-7.2697	0.286399668	0.4938
BCAT1	1.9301	1.8552	1.3989	0.287516127	0.49375
RYR1	-0.2208	-0.0363	-0.6217	0.299292003	0.49315
VTA1	2.3512	2.3501	1.8587	0.284027997	0.49195
DBN1	-0.5094	-0.917	-1.2043	0.349181199	0.4911
CLIC5	-0.6288	-0.74	-1.1752	0.288766757	0.4908
TBCC	-0.6375	-0.6682	-1.1423	0.283000689	0.48945
NOS1	-4.8827	-3.7059	-4.7828	0.652501834	0.4885
DDX58	0.3129	0.7242	0.0301	0.349026823	0.48845
IL36G	-2.295	-4.1984	-3.7344	0.992480052	0.4877
CCL3	-1.1077	-1.0971	-1.5888	0.28087318	0.4864
MSRA	0.7839	0.7513	0.2812	0.281295829	0.4864
FKBP4	-0.3614	-0.4489	-0.8907	0.283725801	0.48555
CNGB3	-0.4279	-0.5869	-0.9925	0.291137287	0.4851
PSTPIP2	-2.0089	-1.7646	-2.3716	0.305418505	0.48485
CRIB-OID3145	0.7699	1.0102	0.4058	0.304305838	0.48425
HMGCS1	1.9762	1.7027	1.3559	0.310870975	0.48355
IQGAP2	3.542	3.6134	3.0943	0.281365142	0.4834
DYNLT3	-0.1702	0.2737	-0.4316	0.356563519	0.48335
CNST	0.5044	0.2771	-0.0916	0.300782585	0.48235
RHOC	0.7759	0.7573	0.2854	0.277976564	0.4812
CD99L2	1.7309	1.809	1.2896	0.280065962	0.48035
NPM1	4.6806	4.6153	4.1682	0.278901494	0.47975
DHODH	0.82	0.4864	0.1738	0.323156866	0.4794
VPS37A	0.2856	0.3976	-0.1354	0.281032027	0.477
UBE2L6	2.8842	2.8636	2.3977	0.275127068	0.4762
RLN2	-7.0289	-6.4424	-7.2078	0.400379949	0.47215
CHRM1	0.0282	-0.311	-0.6132	0.320877817	0.4718
GRSF1	1.0692	1.2266	0.6763	0.283424082	0.4716
VASP	7.3858	7.4084	6.9257	0.2723974	0.4714
LYSMD3	-4.8793	-4.7004	-5.2604	0.286019352	0.47055
CEP164	1.7911	1.7997	1.3266	0.270695961	0.4688
CREG1	-2.1965	-2.0617	-2.5974	0.27864743	0.4683
REG1B	-6.2335	-7.0058	-7.086	0.47075039	0.46635
SLC9A3R1	1.7001	1.847	1.3073	0.279030327	0.46625
TFPI2	-5.2547	-5.012	-5.5979	0.294383067	0.46455
GPRC5C	2.3163	2.5196	1.9535	0.286770506	0.46445
MICALL2	0.2202	-0.3324	-0.5194	0.384565538	0.4633
SRPK2	1.2326	1.2466	0.7763	0.267577957	0.4633
C1QL2	-2.4407	-2.8452	-3.1061	0.335272581	0.46315
PFDN4	0.9927	0.6807	0.374	0.309353783	0.4627

HCLS1	-0.2509	-0.1508	-0.6633	0.271646222	0.46245
APEX1	1.6115	1.707	1.1969	0.271175042	0.46235
DNAJB1	0.0935	0.134	-0.3485	0.267647311	0.46225
GBP6	0.047	0.071	-0.403	0.267005618	0.462
TXLNA	4.5298	4.4748	4.0421	0.267115949	0.4602
FCRL5	-5.4966	-5.4849	-5.9507	0.265616685	0.45995
CTRC	-9.1823	-8.7646	-9.4331	0.33770455	0.45965
LELP1	-0.4255	-1.1959	-1.2692	0.467389691	0.4585
HIP1R	2.7164	2.7188	2.26	0.264198208	0.4576
CARHSP1	-1.6385	-1.1605	-1.8571	0.356258689	0.4576
RPE	1.7021	1.411	1.0991	0.301559784	0.45745
DBH	-7.8417	-7.6125	-8.1837	0.287450309	0.4566
TRIM25	2.5192	2.6954	2.151	0.277785601	0.4563
GPNMB	-5.1218	-4.776	-5.4047	0.31487398	0.4558
CDHR5	-4.7028	-4.825	-5.2197	0.270156294	0.4558
TSC22D1	0.8259	0.4834	0.1997	0.313559771	0.45495
RAD23B	-0.9758	-1.2916	-1.5882	0.306250159	0.4545
DUT	8.3413	8.335	7.8839	0.262280276	0.45425
DARS1	2.1791	2.0812	1.6783	0.265428415	0.45185
CR1	1.0366	0.8464	0.4903	0.277316588	0.4512
SCP2	0.1839	0.5734	-0.0713	0.32467301	0.44995
CCL19	-8.9745	-8.6137	-9.2436	0.316060506	0.4495
LRCH4	-0.772	-0.9885	-1.3279	0.280205109	0.44765
DSG4	-2.3435	-2.3778	-2.8082	0.258961625	0.44755
AMIGO1	0.3396	-0.271	-0.4125	0.399689258	0.4468
PSME2	1.8313	1.7297	1.3339	0.262801243	0.4466
MRPL24	0.1899	0.062	-0.3199	0.265236391	0.44585
NRP2	-3.0667	-2.6283	-3.2931	0.337986824	0.4456
TRAF3	1.089	1.1592	0.6809	0.258277803	0.4432
TBC1D5	-0.0424	-0.0677	-0.4979	0.255992311	0.44285
MT1A_CKMT	-5.1179	-4.9307	-5.465	0.271108447	0.4407
F2R	-2.7866	-2.8994	-3.2827	0.260050617	0.4397
XRCC4	2.2413	2.2091	1.7858	0.254198079	0.4394
LILRB5	-8.7039	-8.65	-9.1163	0.255086476	0.43935
FYB1	2.8002	2.7816	2.3523	0.253396547	0.4386
AGR3	-3.6396	-3.1644	-3.8405	0.347200005	0.4385
TYMP	0.6512	0.6108	0.1931	0.253627371	0.4379
SCIN	1.0633	-0.8659	-0.3392	0.997182011	0.4379
NTproBNP	-2.9192	-3.5205	-3.6577	0.392803551	0.43785
TAG1A_CTAG	-0.3362	-0.2831	-0.7469	0.253838709	0.43725
TMED1	-0.5006	-1.1032	-1.2391	0.393060178	0.4372
LRIG1	-6.7213	-6.3333	-6.9637	0.317990021	0.4364
CBS	2.2538	2.2558	1.8192	0.251495765	0.4356
RCOR1	3.2571	3.1518	2.7695	0.256578494	0.43495
SLITRK6	-3.1439	-3.446	-3.7295	0.292849227	0.43455
LGALS9	1.9706	2.0852	1.5945	0.256700493	0.4334
TIMP4	-6.8324	-7.8125	-7.7552	0.550066532	0.43275
OPLAH	0.338	0.3471	-0.0897	0.249601128	0.43225
HAGH	-4.2491	-3.2297	-4.1693	0.566920418	0.4299
SH3BP1	1.3569	1.4352	0.9675	0.250501677	0.42855

TMSB10	5.7981	5.8686	5.4059	0.249293127	0.42745
BID	-3.344	-3.2808	-3.7398	0.248774624	0.4274
BSND	0.2201	0.3742	-0.1296	0.258150905	0.42675
LSM8	-1.0267	-1.4768	-1.6783	0.333610257	0.42655
LRRC59	2.6965	2.6376	2.2409	0.247794088	0.42615
LMNB1	-0.9626	-1.2195	-1.5167	0.277294146	0.42565
ENTPD6	-1.4903	-1.3206	-1.831	0.259930356	0.42555
GPI	6.7987	6.9595	6.4536	0.258484513	0.4255
BLVRB	1.3521	1.3844	0.943	0.246048796	0.42525
DENR	1.933	1.8769	1.4797	0.247115337	0.42525
CHRDL1	-5.8559	-5.6259	-6.1648	0.270410928	0.4239
ERN1	-2.1868	-2.2843	-2.6592	0.249405299	0.42365
S100A14	-1.2725	-1.8533	-1.9864	0.379626452	0.4235
LYPD3	-6.121	-6.1246	-6.5463	0.244514464	0.4235
RTN4IP1	-1.3073	-1.3193	-1.7357	0.243946552	0.4224
ATXN3	0.3523	0.2219	-0.1339	0.251657254	0.421
KDR	-6.2053	-6.5413	-6.7934	0.295045765	0.4201
PILRB	-1.259	-1.389	-1.744	0.251047804	0.42
EGF	-3.6222	-3.5266	-3.994	0.246926899	0.4196
GFER	2.9821	3.0149	2.5806	0.241831408	0.4179
GFRA2	-3.6514	-3.6153	-4.05	0.241229234	0.41665
CRIP2	-0.9929	-1.2471	-1.5359	0.271683664	0.4159
VPS53	1.0029	0.8755	0.5233	0.248425629	0.4159
PRCP	0.158	0.1664	-0.2536	0.24009898	0.4158
LARP1	1.2789	1.3311	0.8895	0.241304704	0.4155
SNX15	1.94	2.4853	1.7988	0.362530868	0.41385
AMPD3	-1.9024	-1.8815	-2.3054	0.238934098	0.41345
PNPT1	1.0923	1.2646	0.765	0.253775734	0.41345
NUDC	1.8027	1.933	1.455	0.247102334	0.41285
LZTFL1	2.5806	2.8283	2.2926	0.268102524	0.41185
DNAJA1	0.5568	0.6095	0.1719	0.238892954	0.41125
CINP	-0.143	0.3606	-0.3003	0.345240269	0.4091
BIN2	3.0286	2.9852	2.5981	0.237016251	0.4088
SIRPA	0.4841	0.4934	0.0807	0.235633663	0.40805
PTPN1	0.2908	0.0906	-0.2157	0.2550954	0.4064
RNASEH2A	-0.1486	-0.2074	-0.5839	0.236183467	0.4059
IL25	0.9215	0.3906	0.2506	0.353921277	0.40545
MAX	1.2045	1.0091	0.7016	0.253523773	0.4052
CRHR1	0.1292	0.2021	-0.2391	0.236508189	0.40475
NFKB1	1.7299	1.5178	1.2192	0.256568009	0.40465
PRR5	-0.0251	-1.3819	-1.1073	0.717340951	0.4038
PDRG1	-0.3957	-0.4438	-0.8224	0.233710811	0.40265
ARG1	-3.8377	-4.4595	-4.5498	0.38770172	0.4012
CCS	-2.754	-2.8144	-3.1854	0.23359335	0.4012
ATG16L1	0.8082	1.0251	0.5157	0.255633272	0.40095
CCDC50	2.6458	2.6805	2.2623	0.232080295	0.40085
ERI1	1.3107	1.1314	0.8209	0.247811346	0.40015
EGLN1	-1.1982	-0.9894	-1.4921	0.252547665	0.3983
GLRX5	3.2221	3.4767	2.9514	0.262691118	0.398
GOLGA3	1.0908	1.3101	0.803	0.254319923	0.39745

CPPED1	3.457	3.4418	3.0543	0.228237661	0.3951
ATP5IF1	4.6077	4.7749	4.2967	0.242676767	0.3946
CIAPIN1	3.4744	3.5883	3.1382	0.234020533	0.39315
PHYKPL	-2.0083	-1.8749	-2.3341	0.236222296	0.3925
BNIP3L	0.3218	-0.2986	-0.3808	0.384122324	0.3924
DDT	3.4078	3.4415	3.0328	0.226861331	0.39185
FLT3	-1.1425	-1.0958	-1.509	0.226287936	0.38985
CLC	1.2328	1.0947	0.7743	0.235212677	0.38945
BCHE	-6.5075	-6.1956	-6.7378	0.272121462	0.38625
PREB	2.0062	2.4142	1.8246	0.301957701	0.3856
PMM2	1.0038	0.7346	0.4845	0.259708535	0.3847
TDP1	1.2304	1.5349	0.9991	0.268732078	0.38355
MAVS	-2.1175	-1.9516	-2.4178	0.236306771	0.38325
IRAK4	-0.3564	-0.3736	-0.748	0.221292326	0.383
TTF2	2.0184	1.8896	1.5711	0.230256734	0.3829
GOLM2	-3.3131	-3.5259	-3.8017	0.244975999	0.3822
SLC27A4	0.9228	0.4502	0.3048	0.323113994	0.3817
CEP85	3.4203	3.5427	3.1002	0.228492166	0.3813
MET	-3.4862	-4.2278	-4.2351	0.430285769	0.3781
GRAP2	-2.5591	-2.4104	-2.8612	0.229708779	0.37645
IRAK1	-0.0318	0.2694	-0.2549	0.263117699	0.3737
LAMP2	-3.5559	-3.7321	-4.0177	0.233049723	0.3737
SFTPA1	-0.1964	-0.3728	-0.656	0.231858923	0.3714
KHK	-1.6394	-1.7174	-2.0491	0.217548071	0.3707
SIRT2	-1.2771	-1.4123	-1.7153	0.22439076	0.3706
PTGES2	1.152	1.1428	0.7773	0.213726843	0.3701
MYH4	0.5124	-0.0927	-0.1599	0.370281231	0.36975
FGFBP2	-6.5418	-6.6398	-6.9605	0.218998546	0.3697
MPHOSPH8	4.1254	3.941	3.6639	0.232296513	0.3693
ASRGL1	0.2467	0.6102	0.0593	0.280101714	0.36915
CTRB1	-5.7317	-5.6973	-6.081	0.212296781	0.3665
IGF1R	-2.8959	-3.0605	-3.3445	0.226932854	0.3663
CLIP2	0.3871	0.5025	0.0791	0.218879236	0.3657
NAP1L4	-1.0947	-1.1897	-1.5076	0.216245162	0.3654
CAPG	0.6452	0.7406	0.3275	0.216289181	0.3654
RANBP1	0.0856	-0.1862	-0.4155	0.250850201	0.3652
NT5C3A	-1.2665	-0.9093	-1.4524	0.276015779	0.3645
OTUD7B	1.2853	1.1814	0.8695	0.216397189	0.36385
CTBS	-0.5926	-0.6869	-1.0032	0.215069965	0.36345
WASHC3	1.0376	1.0925	0.7018	0.211511284	0.36325
SKAP2	-0.4459	-0.2615	-0.7161	0.22864549	0.3624
CST6	-6.1159	-6.3807	-6.6107	0.247603877	0.3624
ADD1	2.0012	2.0422	1.6604	0.209601559	0.3613
GBP1	-0.1009	-0.4037	-0.6135	0.257702231	0.3612
GH1	-5.4709	-6.2483	-6.2206	0.441053311	0.361
GCC1	0.0475	-0.0187	-0.3463	0.210864348	0.3607
NAAA	-4.6954	-6.0753	-5.7458	0.720651837	0.36045
FAM171A2	-4.3603	-3.5977	-4.3393	0.434352069	0.3603
SUGT1	0.5687	0.43	0.1399	0.218809331	0.35945
LGALS8	3.2348	3.1359	2.8259	0.213338706	0.35945

SMNDC1	2.1389	2.1937	1.8069	0.209300932	0.3594
KLK11	-4.1289	-3.9853	-4.4146	0.21853449	0.3575
COMMD1	0.1092	0.1552	-0.2249	0.207450725	0.3571
NDST1	0.1183	0.3242	-0.1358	0.230420492	0.35705
GOPC	0.7008	0.6762	0.3322	0.206077299	0.3563
NDUFA5	0.2321	0.3755	-0.0519	0.217520237	0.3557
NTF3	-3.1965	-3.2061	-3.5566	0.205188702	0.3553
CEACAM18	-1.4322	-1.3899	-1.7657	0.205846699	0.35465
UBQLN3	0.1649	-0.4952	-0.5183	0.387949279	0.35315
MYO9B	-0.0625	0.1643	-0.3017	0.233027495	0.3526
ACADM	-0.3782	-0.3375	-0.71	0.204329791	0.35215
HMBS	-0.3718	-0.2728	-0.6739	0.208944418	0.3516
DPP7	-1.0118	-0.8705	-1.2927	0.214912129	0.35155
M6PR	0.6393	0.3841	0.1609	0.239378306	0.3508
S100A3	0.551	0.2806	0.0657	0.24317835	0.3501
METAP1D	1.2302	0.9084	0.7197	0.258125674	0.3496
VEGFB	-4.0524	-4.4512	-4.6013	0.283684878	0.3495
OTUD6B	0.9199	0.7947	0.5085	0.210885214	0.3488
TDRKH	0.2146	0.0907	-0.1956	0.210389694	0.34825
MST1	-5.1143	-5.2252	-5.5179	0.208512614	0.34815
NCLN	0.9628	0.8595	0.5633	0.207366656	0.34785
COX6B1	1.7732	1.7914	1.4348	0.200835488	0.3475
GADD45GIP1	1.929	1.6641	1.4501	0.239900403	0.34645
CDC27	2.0746	2.0568	1.7197	0.199961355	0.346
TPBGL	-0.6928	-1.0214	-1.2025	0.258382552	0.3454
HDGFL2	0.9889	1.0398	0.67	0.20043289	0.34435
GLP1R	0.571	0.444	0.1642	0.208127877	0.3433
STX8	1.0893	0.9182	0.6611	0.215534553	0.34265
PRKCQ	0.1587	0.3689	-0.0787	0.2239377	0.3425
CCDC80	-4.5229	-4.4681	-4.8374	0.199288643	0.3419
PGR	-0.9035	-0.8682	-1.2259	0.197119735	0.34005
ILKAP	0.9934	0.8466	0.58	0.209573122	0.34
IL20RA	0.04	-0.7449	-0.6916	0.438586263	0.33915
SDHB	-0.365	-0.5185	-0.7796	0.209614177	0.33785
GRHPR	0.1659	0.1018	-0.2038	0.197559383	0.33765
MELTF	0.7584	0.6084	0.3464	0.208521781	0.337
ESR1	0.6758	0.5489	0.2785	0.202923245	0.33385
MCFD2	0.5772	0.9795	0.4446	0.278551629	0.33375
NECTIN2	-0.0378	0.0417	-0.3318	0.196747935	0.33375
IL2RG	-0.0128	-0.0868	-0.3834	0.196125776	0.3336
SDCCAG8	2.1948	2.4515	1.9904	0.231043813	0.33275
RNF5	0.451	0.3976	0.0924	0.193473754	0.3319
TRAF2	1.21	1.1383	0.8428	0.19463512	0.33135
TIMM10	3.5517	3.4758	3.1824	0.195032843	0.33135
YARS1	0.2084	0.6804	0.1132	0.303744103	0.3312
IGHMBP2	0.7653	0.5424	0.3229	0.221202178	0.33095
ZNF75D	-0.7106	-1.1753	-1.2729	0.300458855	0.32995
DPT	-8.9247	-9.011	-9.2977	0.195266305	0.32985
LCN15	-1.4563	-1.2134	-1.6645	0.225772326	0.32965
IFIT1	-0.5761	-0.1879	-0.7114	0.271740556	0.3294

RABGAP1L	-0.8774	-0.1437	-0.8393	0.413042907	0.32875
CBX2	-0.0691	-0.1047	-0.4152	0.190378054	0.3283
TRPV3	0.5216	0.547	0.2062	0.189853874	0.3281
MRI1	-0.3495	-0.4152	-0.7101	0.192056788	0.32775
CWC15	2.4831	2.5948	2.2135	0.196023272	0.32545
COPE	0.4156	0.1839	-0.025	0.220398299	0.32475
REG4	-11.0623	-8.7966	-10.2536	1.148204481	0.32415
APPL2	0.4558	0.8843	0.3481	0.28364355	0.32195
FASLG	-8.0369	-7.7061	-8.1934	0.248791137	0.3219
CHEK2	2.3705	2.1593	1.9444	0.213052677	0.3205
LTBR	-1.6526	-1.4656	-1.8796	0.207321811	0.3205
ZNRD2	2.3781	2.3407	2.0402	0.185236524	0.3192
IL10RB	-4.5424	-4.9148	-5.0476	0.261898403	0.319
DDA1	1.7823	1.5227	1.3336	0.225271192	0.3189
PPME1	2.2782	2.2919	1.9671	0.18369628	0.31795
RAB27B	1.9409	1.8096	1.5588	0.194139443	0.31645
ARHGAP45	0.2784	0.7884	0.2171	0.313645601	0.3163
ANK2	0.3761	-0.0081	-0.1318	0.26485019	0.3158
FLRT2	-4.5707	-5.3206	-5.2606	0.416715734	0.31495
VAT1	-1.9066	-1.8072	-2.1709	0.187977188	0.314
BACH1	0.7796	0.7295	0.441	0.182753121	0.31355
MED18	0.2596	0.4396	0.0364	0.201985346	0.3132
TUBB3	4.8366	4.8354	4.5236	0.180365222	0.3124
NEDD9	-2.2291	-1.6947	-2.2739	0.322248103	0.312
ABL1	0.0514	0.0717	-0.2496	0.179929051	0.31115
DDX25	1.0562	0.5171	0.4756	0.323894896	0.31105
LY9	-7.1292	-7.4075	-7.5792	0.227094613	0.31085
SEZ6	-2.1642	-2.0832	-2.4339	0.183616257	0.3102
TNIP1	2.1073	2.0946	1.7909	0.179120044	0.31005
FXN	0.8997	0.9576	0.6188	0.181219324	0.30985
FGF6	-0.1653	-0.0301	-0.4069	0.190887332	0.3092
DAG1	-0.2679	-0.1065	-0.4955	0.195436571	0.3083
PACS2	0.8171	0.5744	0.388	0.215164689	0.30775
CTSH	-4.4915	-4.4911	-4.7978	0.176957971	0.3065
PDZK1	-1.2316	-0.8004	-1.3223	0.278848687	0.3063
OGA	0.3819	0.4997	0.1347	0.18628369	0.3061
IDUA	-4.0723	-3.9258	-4.3042	0.19079938	0.30515
FAM172A	-0.0674	-0.0416	-0.3593	0.176448548	0.3048
SBSN	-1.9704	-1.8204	-2.1993	0.190814229	0.3039
PXDNL	-2.005	-1.0637	-1.8382	0.50228116	0.30385
TSPAN8	-5.2206	-5.3524	-5.5902	0.187316239	0.3037
GIMAP7	-3.7191	-3.096	-3.7095	0.357007941	0.30195
FBN2	2.5661	2.2775	2.1204	0.226060044	0.3014
CLNS1A	1.4117	1.1762	0.9926	0.210084911	0.30135
COMP	-7.2104	-7.0518	-7.4321	0.191020479	0.301
SEMA4D	0.2681	0.0476	-0.1411	0.204805835	0.29895
WDR46	-1.0654	-0.775	-1.219	0.225485077	0.2988
CFHR5	-8.9271	-8.8016	-9.1629	0.18343463	0.29855
MAD1L1	2.6005	2.571	2.2872	0.172997871	0.29855
CDH22	-0.3324	-0.0179	-0.4736	0.233277439	0.29845

TK1	1.0287	1.3065	0.8692	0.221300843	0.2984
CMIP	-0.0682	-0.2129	-0.4385	0.18661705	0.29795
SAFB2	0.8515	0.6988	0.4774	0.188098405	0.29775
NFX1	-1.2591	-0.4746	-1.1642	0.428173333	0.29735
NSFL1C	2.2766	2.1986	1.9418	0.175176711	0.2958
SNX2	-0.2649	-0.1795	-0.5177	0.175869383	0.2955
ADAM15	-5.1485	-5.7236	-5.7314	0.334308555	0.29535
KIF20B	2.192	1.9181	1.7597	0.218706447	0.29535
PRKD2	0.7573	1.0767	0.6223	0.233352637	0.2947
ARHGEF12	1.0067	0.8086	0.613	0.196851323	0.29465
SMAD3	0.249	0.3077	-0.0161	0.172515767	0.29445
MORN4	0.0517	-0.7984	-0.6671	0.457635798	0.29375
CCL7	-1.5314	-1.3608	-1.7397	0.189762334	0.2936
DNAJB6	3.0058	3.1837	2.8014	0.191303014	0.29335
RWDD1	4.4318	4.4775	4.1617	0.170671292	0.29295
MB	-5.4887	-5.9634	-6.0186	0.291313445	0.29255
TAX1BP1	3.4858	3.4238	3.1623	0.17169668	0.2925
FRMD7	-0.7326	-0.6246	-0.9697	0.176528761	0.2911
TXN	5.591	5.7885	5.3993	0.194607203	0.29045
TNFSF11	-1.3339	-2.538	-2.2258	0.624874822	0.28985
CDC123	-0.0368	-0.7287	-0.672	0.384148309	0.28925
DCXR	0.2827	0.1882	-0.0534	0.173332061	0.28885
THBS2	-4.4029	-4.1635	-4.5714	0.204974397	0.2882
CADPS	-0.3834	-0.6319	-0.7956	0.207548701	0.28795
PLG	-5.206	-4.9878	-5.3842	0.198536076	0.2873
DKK1	1.0704	1.0395	0.7684	0.166159572	0.28655
IL13RA1	-3.8612	-3.4137	-3.9236	0.278133068	0.28615
PRDX3	0.6931	0.788	0.4548	0.171665926	0.28575
IFNG	-2.3921	-2.3014	-2.6313	0.170429428	0.28455
CCL2	3.6627	3.8636	3.4797	0.192019539	0.28345
NEK7	-0.5613	-1.283	-1.2049	0.396057963	0.28275
PPP2R5A	2.2205	2.5504	2.1028	0.232031988	0.28265
SLAMF1	-1.5476	-1.7722	-1.9425	0.198071225	0.2826
SOD2	-1.4101	-1.5899	-1.7825	0.186236659	0.2825
LAT	0.3494	0.6532	0.219	0.22279596	0.2823
MYL1	-0.3209	-0.5724	-0.7279	0.205378309	0.28125
TLR2	-0.4369	-0.4099	-0.7042	0.162681068	0.2808
STAT2	-0.8504	-0.2281	-0.82	0.350838771	0.28075
CDC26	1.1377	1.0839	0.8301	0.164279558	0.2807
TOMM20	5.0342	4.8925	4.6831	0.176634491	0.28025
STX3	0.5698	0.3161	0.1627	0.205598987	0.28025
MZT1	0.1663	0.3679	-0.013	0.190558766	0.2801
CAPS	-1.9816	-2.1544	-2.3479	0.183247456	0.2799
RASSF2	-2.3134	-2.0894	-2.4812	0.196570632	0.2798
CES3	-4.9804	-5.3332	-5.4364	0.239114366	0.2796
NFKB2	-1.0265	-1.3015	-1.442	0.211347068	0.278
RUVBL1	0.1927	0.3182	-0.0225	0.17230679	0.27795
CALCOCO1	0.8784	1.0055	0.6642	0.172492386	0.27775
USP25	1.9022	1.9115	1.6294	0.16025331	0.27745
KLRD1	-8.802	-8.7389	-9.0476	0.163093358	0.27715

CAMLG	-0.2688	-0.368	-0.5928	0.166007871	0.2744
BAG4	0.8068	0.6452	0.453	0.177120411	0.273
NECAP2	-0.6387	-1.393	-1.2884	0.40866036	0.27255
NINJ1	-0.8308	-0.3784	-0.877	0.2755002	0.2724
PRELP	-10.4179	-11.7007	-11.3317	0.660399788	0.2724
C1QTNF1	-3.6999	-4.0074	-4.1259	0.21987667	0.27225
INPPL1	-0.5528	-0.5843	-0.8403	0.157683491	0.27175
WWP2	-0.7733	-0.9832	-1.1498	0.188664526	0.27155
CHM	0.9296	0.5911	0.4902	0.230157707	0.27015
CLGN	-1.843	-1.5974	-1.9895	0.198126231	0.2693
VEGFD	-9.2961	-9.1141	-9.4739	0.179904086	0.2688
CD101	-2.3926	-2.5792	-2.7545	0.1809794	0.2686
GFRA1	-8.405	-8.746	-8.8439	0.230397707	0.2684
SPART	5.6528	5.6555	5.3863	0.154649162	0.26785
DXO	1.111	0.9956	0.7862	0.16465143	0.2671
PBK	2.4562	2.3996	2.1613	0.156501576	0.2666
SF3B4	2.1383	1.906	1.7561	0.192574721	0.26605
FGF9	-0.2013	-0.5948	-0.6625	0.249041824	0.26445
UNG	0.9479	0.8994	0.6593	0.154537061	0.26435
SEPTIN3	-2.2061	-2.1355	-2.4349	0.156511001	0.2641
PILRA	1.2196	1.4457	1.0687	0.189745891	0.26395
GMFG	2.713	2.683	2.4345	0.152869498	0.2635
GART	0.4758	0.2605	0.1051	0.186154837	0.26305
CLPP	1.6322	1.3813	1.2439	0.19689526	0.26285
SMS	0.4155	0.446	0.1687	0.152061271	0.26205
NPTXR	-4.5965	-4.637	-4.878	0.152186016	0.26125
PKD2	2.0143	1.9634	1.7283	0.152566281	0.26055
ERBIN	0.0244	0.1399	-0.1783	0.161079059	0.26045
ATXN10	1.6349	2.0001	1.5575	0.236381246	0.26
RILPL2	2.0342	2.0812	1.7978	0.151882367	0.2599
AXIN1	-0.1398	-0.2607	-0.4599	0.161638207	0.25965
WFDC1	-2.7157	-2.1052	-2.6694	0.33989596	0.25895
CD72	-1.4478	-1.4508	-1.7076	0.149137118	0.2583
LRFN2	-0.3547	-0.5167	-0.694	0.169707484	0.2583
SH2D1A	-0.6948	-1.0345	-1.1226	0.225894717	0.25795
SUGP1	0.7763	0.6091	0.4349	0.17071196	0.2578
LACRT	-0.4177	-0.5212	-0.7272	0.15755343	0.25775
SEZ6L2	-7.274	-5.8979	-6.843	0.703873357	0.25705
IFT20	1.0558	0.7969	0.6696	0.196801482	0.25675
COX5B	0.4277	0.1394	0.0269	0.206725978	0.25665
PPIE	0.1276	0.1762	-0.1046	0.150070783	0.2565
POLR2A	0.0862	0.1929	-0.1168	0.157325554	0.25635
DTNB	0.7936	0.8691	0.5768	0.151735395	0.25455
HDDC2	0.3389	0.5876	0.2091	0.192337369	0.25415
SLC34A3	-0.1255	0.3897	-0.122	0.296445661	0.2541
CLEC4D	-6.0233	-5.6054	-6.0679	0.255126054	0.25355
AKT1S1	-0.2197	-0.4736	-0.5995	0.193461478	0.25285
LTO1	0.5238	0.9323	0.4755	0.250955301	0.25255
EBAG9	1.3096	1.1919	0.9982	0.15723811	0.25255
USP8	0.4665	0.0163	-0.0108	0.268088835	0.2522

SLMAP	-0.7227	-0.8884	-1.0575	0.167402877	0.25195
SERPINA9	-5.4938	-5.1613	-5.5779	0.220297004	0.25035
TMPRSS5	-6.5712	-6.5803	-6.8252	0.14409188	0.24945
DDR1	-4.1294	-4.1422	-4.3852	0.144133318	0.2494
CCDC28A	0.6265	0.0829	0.1062	0.307342355	0.2485
ATP5PO	0.5431	-0.3329	-0.143	0.460827375	0.2481
CRADD	-1.969	-2.214	-2.339	0.188215302	0.2475
SEL1L	0.6637	0.5537	0.3636	0.151821178	0.2451
BCL2L11	-4.0152	-2.3241	-3.4122	0.857067658	0.24255
PDCD1	-5.2183	-4.5634	-5.1334	0.356137151	0.24255
CLSTN2	-8.2926	-8.5776	-8.6772	0.199608918	0.2421
HPCAL1	0.7338	0.8056	0.5301	0.142915581	0.2396
KIAA2013	-0.2346	0.0232	-0.3445	0.188742391	0.2388
LPCAT2	2.0567	2.0047	1.7931	0.139620629	0.2376
IVD	0.9087	-0.1628	0.1367	0.552840619	0.23625
STEAP4	-0.2398	-0.5897	-0.6509	0.221802713	0.23615
CEACAM16	-7.447	-6.8447	-7.3816	0.330480473	0.23575
HSP90B1	-0.1857	-0.3509	-0.5033	0.158842983	0.235
SNX9	5.1229	5.1634	4.9094	0.136466418	0.23375
APRT	-1.8988	-2.302	-2.3338	0.24248934	0.2334
CGA	-2.4853	-2.4303	-2.6911	0.13747441	0.2333
INPP5D	0.5594	0.2869	0.19	0.191529902	0.23315
MOCS2	3.3056	3.4772	3.1591	0.15921496	0.2323
APBB1IP	-4.1281	-4.3558	-4.4742	0.175902937	0.23225
PPM1F	0.4625	0.1585	0.0787	0.202520155	0.2318
DDI2	0.5354	0.2376	0.1551	0.200049652	0.2314
ADAMTS4	0.2565	-0.2487	-0.2261	0.285377107	0.23
ATOX1	2.8634	2.915	2.6596	0.135047004	0.2296
BCAM	1.1156	1.2191	0.938	0.142168456	0.22935
TLR4	1.0851	0.357	0.4917	0.387383802	0.22935
HNRNPK	1.8089	1.7079	1.5296	0.14142158	0.2288
CENPJ	-0.4671	0.4224	-0.2509	0.463911913	0.22855
ZHX2	1.4668	1.259	1.1345	0.167881099	0.2284
DNAJC9	-0.3262	-0.2122	-0.4973	0.143499837	0.2281
SOWAHA	-0.2199	-0.864	-0.77	0.347924997	0.22805
GAS2	3.2778	3.1317	2.9767	0.150571921	0.22805
IMPG1	0.0214	0.1804	-0.127	0.153730457	0.2279
RSPO3	-4.653	-4.7163	-4.9123	0.13519084	0.22765
PHOSPHO1	-1.5629	-1.6344	-1.8261	0.136097624	0.22745
AP1G2	-1.3137	-0.999	-1.3836	0.204873644	0.22725
PDLIM7	4.6113	4.6735	4.4154	0.134697971	0.227
GLYR1	0.2179	0.1516	-0.0422	0.135158019	0.22695
LGALS4	-7.9599	-6.9524	-7.683	0.520497554	0.22685
TNFAIP8L2	-0.5293	-0.6662	-0.8245	0.147729223	0.22675
GPKOW	1.1014	0.9977	0.8233	0.140539828	0.22625
PLA2G15	-3.6586	-3.6966	-3.9038	0.131971512	0.2262
CA3	-4.6564	-5.0797	-5.0942	0.248683862	0.22615
SSB	2.7046	2.4174	2.3352	0.193948894	0.2258
GSN	-4.0817	-4.1098	-4.3213	0.13097711	0.22555
BCL7B	0.2561	-0.4635	-0.3287	0.382532404	0.225

FKBPL	-0.2141	-0.262	-0.463	0.132064769	0.22495
NEO1	0.1508	0.5268	0.1149	0.228154341	0.2239
PTN	-1.6765	-2.7341	-2.4287	0.544302575	0.2234
GABARAPL1	-0.4599	-0.1702	-0.5382	0.193856038	0.22315
CLEC4G	-6.3487	-6.672	-6.733	0.206531023	0.22265
CACYBP	5.0008	5.112	4.834	0.139923598	0.2224
PFKFB2	-1.411	-1.2721	-1.5623	0.145144147	0.22075
TMED4	0.2494	-0.1585	-0.1748	0.240344801	0.22025
RAB6A	-2.0776	-2.0381	-2.2765	0.12777325	0.21865
BECN1	0.4475	0.2704	0.1405	0.154103547	0.21845
PRSS2	-5.377	-5.5243	-5.6682	0.145603308	0.21755
NGRN	0.3712	0.4097	0.1734	0.126783792	0.21705
NFATC3	0.7998	0.9695	0.6684	0.150955435	0.21625
APP	1.3624	1.4887	1.2093	0.139914057	0.21625
ACP1	0.9347	0.4258	0.4644	0.283328802	0.21585
NCR3LG1	-5.6104	-4.8565	-5.4492	0.396997888	0.21575
FMNL1	0.3826	-0.0541	-0.051	0.251238751	0.21525
MINK1	0.8247	0.6157	0.505	0.162349202	0.2152
IL18	-2.1077	-2.1636	-2.3503	0.127040978	0.21465
TJAP1	4.8391	4.8418	4.626	0.123820125	0.21445
REPS1	1.1252	1.0863	0.8918	0.125046005	0.21395
UNC5D	-0.7136	-0.7578	-0.9494	0.125343422	0.2137
CPA1	-7.1447	-7.6973	-7.6345	0.302548795	0.2135
TMEM106A	-1.2461	-1.0971	-1.3842	0.143584482	0.2126
C1QA	-5.8054	-5.7846	-6.0069	0.122781771	0.2119
PRKAG3	0.3821	0.1133	0.0368	0.181354983	0.2109
DNAJB14	1.6193	1.6983	1.4493	0.127241241	0.2095
PRSS27	-5.1673	-5.5151	-5.5505	0.21176254	0.2093
GCNT1	-3.3897	-3.1299	-3.4691	0.177415257	0.2093
IGSF9	-4.0993	-4.3928	-4.4549	0.189934208	0.20885
CTSC	-1.156	-1.2991	-1.4354	0.139713791	0.20785
SEPTIN8	3.3952	3.3636	3.1724	0.1205514	0.207
SAT1	-0.3655	-0.641	-0.7094	0.182046706	0.20615
PTPN6	2.0894	2.2584	1.9684	0.145660564	0.2055
DCTN1	0.4004	0.6779	0.3346	0.182204455	0.20455
NECTIN4	-4.3791	-3.7358	-4.2619	0.342625145	0.20445
TALDO1	4.5858	4.651	4.4144	0.122207856	0.204
DTX3	0.1302	0.2774	0	0.138786791	0.2038
HRAS	0.155	0.3035	0.0255	0.139108171	0.20375
PSAP	4.1275	4.1977	3.9592	0.122566431	0.2034
TEX33	0.2314	-0.1543	-0.1647	0.225746118	0.20325
REEP4	1.4366	1.3514	1.1922	0.124053107	0.2018
CCND2	-0.9717	-1.1833	-1.2791	0.157293229	0.2016
MAGED1	-1.7097	-1.7634	-1.9378	0.119253693	0.20125
DUSP13	-3.6588	-3.5317	-3.7961	0.132232787	0.20085
HSBP1	2.5	2.5543	2.3267	0.118871878	0.20045
CIRBP	0.1068	0.4464	0.0762	0.205472042	0.2004
SEPTIN7	2.86	2.8845	2.6725	0.115974494	0.19975
MMP7	-7.656	-7.4021	-7.7285	0.171395459	0.19945
FGF5	-2.7635	-3.1702	-3.1642	0.23309561	0.19735

SCRN1	-0.976	-1.3516	-1.3611	0.219646542	0.1973
FMR1	2.3039	2.3835	2.1465	0.120609508	0.1972
PDE1C	1.977	1.9456	1.7649	0.11447333	0.1964
TGFB1	-4.7031	-5.0654	-5.0798	0.213452391	0.19555
CLSTN1	-1.0557	-0.9722	-1.2094	0.120318868	0.19545
CDKN2D	-2.2407	-1.9374	-2.2843	0.188958311	0.19525
ERC2	-0.5547	-0.0366	-0.4907	0.282468411	0.19505
TACC3	0.4147	0.2029	0.1147	0.154185213	0.1941
BTN1A1	-1.8078	-1.6276	-1.9109	0.1433879	0.1932
TFRC	-0.0216	0.2375	-0.0848	0.17078463	0.19275
MYH9	7.6468	7.6434	7.4554	0.109536539	0.1897
CAMSAP1	2.0684	1.9017	1.7958	0.13742541	0.18925
STC2	0.8952	0.9051	0.7113	0.109144904	0.18885
CDC42BPB	-0.0693	0.2291	-0.1087	0.184708672	0.1886
PPT1	-1.3931	-1.4451	-1.6072	0.111668572	0.1881
DDHD2	-0.8029	-0.6548	-0.9169	0.131419189	0.18805
SEPTIN9	1.5331	1.4597	1.3084	0.114578459	0.188
CCL21	-4.7843	-5.086	-5.1231	0.185824622	0.18795
DHPS	2.1218	2.4338	2.0899	0.19001264	0.1879
IL6R	-5.4345	-5.1243	-5.4672	0.189241354	0.1878
IKZF2	0.0819	0.3853	0.0464	0.186263711	0.1872
GNE	2.2689	2.5392	2.2169	0.173033417	0.18715
TMPRSS11D	-3.5191	-2.8222	-3.3575	0.364767108	0.18685
CCL28	-2.4035	-3.0012	-2.8883	0.317548616	0.18595
KLK8	-6.4288	-5.7579	-6.2789	0.352140554	0.18555
CX3CL1	-6.0391	-6.14	-6.2745	0.118098984	0.18495
HSPA1A	3.5411	3.6673	3.4196	0.123857431	0.1846
BRK1	0.5683	0.4964	0.348	0.112341933	0.18435
CETN3	3.1779	3.2535	3.0317	0.112757143	0.184
PSRC1	-0.6887	0.1177	-0.4681	0.416754716	0.1826
GPR158	-0.7423	-0.6461	-0.876	0.115458607	0.1818
FUOM	-4.8808	-5.1404	-5.1923	0.166892191	0.1817
PEAR1	-3.1563	-3.2054	-3.362	0.107429713	0.18115
GJA8	-1.8705	-1.4258	-1.8292	0.245694709	0.18105
TIMP1	5.1024	5.2586	5.0001	0.130183191	0.1804
EFNA4	-0.4174	-0.5253	-0.6516	0.117220405	0.18025
MSR1	-6.3189	-6.5571	-6.6182	0.158141782	0.1802
PTRHD1	1.9472	2.0883	1.8386	0.12520201	0.17915
DOK1	-1.977	-2.8043	-2.5685	0.426204016	0.17785
RAB6B	-2.6756	-2.6237	-2.8273	0.105798125	0.17765
C2CD2L	0.6341	0.4944	0.3875	0.123663023	0.17675
MZB1	-7.3632	-7.3573	-7.5359	0.101454473	0.17565
ADH1B	-3.5887	-2.8031	-3.3712	0.405629392	0.1753
YY1	0.6904	0.3482	0.3441	0.198763402	0.1752
BMP4	-6.9086	-6.2877	-6.7732	0.326486401	0.17505
TNFSF13	-6.061	-6.1883	-6.299	0.119096446	0.17435
SPINT1	2.2076	2.286	2.0726	0.107943751	0.1742
SNAP25	1.5643	1.6914	1.4539	0.118847816	0.17395
TNFSF8	-2.915	-2.1676	-2.7141	0.386788017	0.1728
TGFA	-1.6824	-1.6969	-1.8623	0.099942834	0.17265

EIF4E	0.4943	0.4528	0.3009	0.101816354	0.17265
KCTD5	4.1088	3.9946	3.8794	0.114700363	0.1723
CCN3	-4.6957	-4.396	-4.718	0.179815359	0.17215
CD6	-6.1769	-7.3908	-6.9554	0.614978132	0.17155
NCR1	-3.2183	-2.9573	-3.2588	0.163637557	0.171
TARS1	0.7517	0.6847	0.5483	0.10365449	0.1699
SLIRP	0.4891	0.2862	0.2178	0.141097283	0.16985
FGR	-0.6496	-0.8771	-0.933	0.150109194	0.16965
FUT1	-0.9398	-1.7156	-1.4969	0.400011612	0.1692
PDGFRB	-4.2487	-4.2186	-4.4014	0.098012873	0.16775
C7orf50	0.5104	0.3786	0.2776	0.116739082	0.1669
ENDOU	-3.1931	-3.6033	-3.565	0.226583517	0.1668
THSD1	-1.7748	-0.5682	-1.338	0.610910501	0.1665
PPM1A	0.2575	0.3179	0.1231	0.099715061	0.1646
CMC1	1.5877	1.4087	1.3338	0.130458308	0.1644
ALPI	-3.6233	-3.4617	-3.7067	0.124562648	0.1642
DNAJA4	-0.1932	0.0137	-0.2532	0.140025724	0.16345
SCGN	-1.4214	-2.3998	-2.0732	0.498126048	0.1626
LPO	-6.5361	-6.855	-6.8579	0.184959842	0.16235
MEGF9	-4.7567	-4.4964	-4.7887	0.160322269	0.16215
CDC37	2.5321	2.4853	2.347	0.096245467	0.1617
GLO1	6.0244	6.0033	5.853	0.093464129	0.16085
HIP1	-0.1021	-0.0551	-0.2389	0.095486194	0.1603
RNASE1	-1.5666	-1.3001	-1.5935	0.162187864	0.16015
MYL4	0.7758	0.7133	0.5849	0.0973273	0.15965
KLK12	-4.1138	-2.6792	-3.556	0.723186956	0.1595
LRP2BP	-2.4925	-2.7985	-2.8049	0.178545382	0.1594
ARG2	0.7518	0.6034	0.5192	0.117767398	0.1584
AFAP1	0.4291	0.7117	0.4123	0.168218786	0.1581
VASH1	0.3441	0.2456	0.1371	0.10354025	0.15775
RGS10	0.2707	-0.1112	-0.0779	0.211533473	0.15765
GIGYF2	3.2162	3.3371	3.119	0.109264404	0.15765
PSME1	1.3944	1.3636	1.2214	0.092284415	0.1576
ARTN	-0.2188	-0.181	-0.3571	0.092706472	0.1572
PTTG1	-0.5908	-0.3516	-0.6282	0.150068296	0.157
DLG4	0.8921	0.8734	0.7258	0.091096231	0.15695
LAIR1	2.4015	2.5934	2.3406	0.131935755	0.15685
SFRP4	-5.8096	-5.6136	-5.8669	0.132828323	0.1553
OGT	0.0697	-1.021	-0.6303	0.552610921	0.15465
TMEM25	-3.6616	-3.9945	-3.9826	0.188858421	0.15455
CD207	-4.079	-3.2869	-3.8371	0.405926525	0.15415
ADAMTS1	-0.3746	-1.1166	-0.8996	0.381505352	0.154
C1QTNF9	-6.1009	-6.1229	-6.2659	0.089589806	0.154
PDAP1	4.2302	4.1645	4.0434	0.094759292	0.15395
IOD1-OID310	-2.2452	-2.3369	-2.4445	0.099755651	0.15345
DNER	-3.2782	-3.1441	-3.3645	0.111060539	0.15335
PLA2G7	-6.424	-6.5091	-6.6192	0.097866457	0.15265
BDNF	-2.2264	-1.2484	-1.8887	0.496740942	0.1513
EVI5	2.4032	2.5984	2.3496	0.130943652	0.1512
KITLG	-3.8799	-4.788	-4.4848	0.46232742	0.15085

SSC4D	-1.1353	-1.2211	-1.3289	0.09700811	0.1507
CNTNAP4	-0.105	-0.1454	-0.2759	0.089320789	0.1507
CXCL17	-5.0523	-5.2289	-5.2912	0.123923404	0.1506
TP53	2.8042	2.7787	2.6411	0.087735987	0.15035
PDIA3	0.9102	0.8425	0.7263	0.093009802	0.15005
TFF1	-2.8625	-3.0189	-3.0904	0.116555866	0.1497
PON2	-1.1581	-1.1672	-1.3122	0.086462535	0.14955
INHBB	-2.1136	-1.8655	-2.1384	0.15091005	0.14885
PSG1	-3.328	-3.3931	-3.509	0.091680441	0.14845
ZNF174	0.169	-0.0259	-0.0763	0.129549386	0.14785
SPRING1	-0.6566	-0.855	-0.9035	0.130814385	0.1477
SMAD2	-0.1596	-0.0958	-0.2735	0.090019387	0.1458
FGF3	0.2431	0.2485	0.1004	0.083990138	0.1454
TLR1	-0.7468	-1.1517	-1.0944	0.219109204	0.14515
FCRL3	-0.0186	-0.5398	-0.4237	0.273628294	0.1445
CEP170	1.3764	1.3752	1.2328	0.082563269	0.143
ARSB	-3.1451	-3.2637	-3.3473	0.101603609	0.1429
NRCAM	1.3324	1.3733	1.2101	0.084915978	0.14275
CTSD	2.8743	3.0744	2.8316	0.129624548	0.14275
ELAC1	3.2336	3.4785	3.2143	0.14728099	0.14175
GALNT3	-2.6421	-4.1512	-3.5371	0.758894659	0.14045
AARSD1	2.5095	2.5221	2.3762	0.080843944	0.1396
CD244	-2.0596	-1.9247	-2.1312	0.104854518	0.13905
DYNC1H1	0.3503	0.2578	0.1652	0.092550005	0.13885
NENF	0.1035	0.2714	0.049	0.115918376	0.13845
MAP1LC3B2	-0.0988	-0.1185	-0.2469	0.080424146	0.13825
ITGB7	-3.5201	-3.5065	-3.6514	0.080021518	0.1381
DAPP1	-2.4651	-2.9561	-2.8484	0.258069493	0.1378
PAGR1	2.2185	2.2877	2.1157	0.086545248	0.1374
PHACTR2	0.5562	0.2466	0.2643	0.173863481	0.1371
MORF4L2	1.3551	1.2328	1.1574	0.099772859	0.13655
LY6D	-5.1013	-4.8403	-5.1061	0.152092998	0.1353
PAG1	3.8829	4.213	3.9131	0.182491123	0.13485
CD164L2	0.724	0.3885	0.4223	0.184718516	0.13395
C8B	-2.4747	-2.7303	-2.7356	0.149124255	0.1331
STC1	-3.919	-4.4698	-4.3271	0.285858222	0.1327
ARHGEF1	-0.8386	-0.8382	-0.9703	0.076152763	0.1319
DSCAM	-1.1976	-0.9902	-1.2255	0.128555604	0.1316
B2M	6.6081	6.7819	6.5636	0.115355667	0.1314
GASK1A	-2.2754	-2.0395	-2.2877	0.139882891	0.13025
CTHRC1	3.6006	3.5746	3.4587	0.075547358	0.1289
REG1A	-7.2412	-7.2571	-7.378	0.074815172	0.12885
MAP4K5	-0.6319	-0.9454	-0.9175	0.173506974	0.12885
TTN	-0.7005	-0.73	-0.8437	0.075613248	0.12845
KLK1	-8.3241	-7.8783	-8.2294	0.234867672	0.1282
BMP10	-4.2005	-3.8113	-4.134	0.208180282	0.1281
DGKA	0.529	0.7733	0.5237	0.142601274	0.12745
HGS	0.3212	0.5822	0.3243	0.149801546	0.1274
LEO1	1.815	1.5434	1.5518	0.154440582	0.1274
AMY2B	-6.1134	-6.3486	-6.3584	0.138708375	0.1274

ACRBP	-0.4317	-0.8559	-0.7708	0.224416451	0.127
TSC1	0.7207	-1.0019	-0.2674	0.864405636	0.1268
TCL1A	-5.9236	-5.7261	-5.9513	0.122806474	0.12645
IL33	0.5297	0.0225	0.1501	0.263827519	0.126
PPP3R1	1.7735	1.6763	1.5991	0.087390922	0.1258
RAB44	-0.6146	-0.6297	-0.7472	0.072591345	0.12505
DKKL1	-3.4046	-3.2436	-3.4488	0.10799821	0.1247
RABEP1	4.0698	4.2744	4.0476	0.125028157	0.1245
CHRDL2	-2.6224	-2.6858	-2.7783	0.078401339	0.1242
SPOCK1	-3.4099	-3.4051	-3.5316	0.071689353	0.1241
NT5C1A	2.3399	2.3641	2.2286	0.072265229	0.1234
NFU1	1.7545	1.7148	1.612	0.073541576	0.12265
GYS1	-0.1066	-0.0035	-0.1773	0.087401888	0.12225
EDF1	0.3165	0.5064	0.2893	0.118275286	0.12215
ACP5	-2.1818	-2.3031	-2.3637	0.092622585	0.12125
UBE2B	1.0606	1.053	0.9358	0.069962657	0.121
CUZD1	0.3978	-0.1679	-0.0056	0.291287184	0.12055
CASP2	-1.4858	-1.769	-1.7471	0.157564558	0.1197
TXNDC15	-4.608	-3.8862	-4.3668	0.367457263	0.1197
IFNGR2	-3.0144	-3.0727	-3.1632	0.074978419	0.11965
TOM1L2	-0.04	-1.051	-0.6647	0.510163173	0.1192
ATXN2L	0.9321	0.8674	0.7808	0.075913701	0.11895
NOS3	1.0947	1.0068	0.9318	0.081535084	0.11895
NCF2	-6.0232	-6.0389	-6.1499	0.069065645	0.11885
AMOT	-0.3828	-0.4364	-0.5284	0.07363912	0.1188
UBXN1	3.7411	4.0869	3.7958	0.185880311	0.1182
MORC3	0.7572	0.8499	0.6855	0.082423237	0.11805
NMT1	0.4117	0.2423	0.2094	0.10855418	0.1176
GIT1	0.962	0.8701	0.7985	0.081959767	0.11755
APOD	-2.9502	-3.1577	-3.1711	0.123849788	0.11715
YWHAQ	3.7628	3.7827	3.6558	0.068250299	0.11695
GNAS	-1.7884	-1.7264	-1.8743	0.074271147	0.1169
FOXO1	-0.5111	-0.2924	-0.5183	0.128395444	0.11655
NFYA	0.2778	0.0471	0.0461	0.133484319	0.11635
IL20RB	0.2119	0.912	0.4458	0.356415408	0.11615
TEF	-1.1858	-2.0134	-1.7157	0.419193881	0.1161
RAP1A	-0.1609	-0.3665	-0.3797	0.122691374	0.116
TFF3	-3.31	-3.0081	-3.2742	0.164941636	0.11515
INPP1	-0.6959	-0.6544	-0.7899	0.069424419	0.11475
HSDL2	-2.3983	-2.4816	-2.5544	0.078108834	0.11445
DCC	-0.238	-0.3034	-0.3841	0.0731834	0.1134
CNTN3	-5.1137	-4.9664	-5.1534	0.09852443	0.11335
IDS	-0.1078	-0.0811	-0.2075	0.066620743	0.11305
IZUMO1	1.1589	1.1351	1.0341	0.066260194	0.1129
NCAN	-8.5064	-7.9809	-8.3563	0.270679829	0.11265
EDN1	-2.2205	-3.0846	-2.7647	0.436874974	0.11215
HNF1A	0.3122	-0.9961	-0.4537	0.657324053	0.11175
PCYT2	-0.9716	-0.9757	-1.0851	0.064378335	0.11145
IOD1-OID314	-1.954	-2.053	-2.1135	0.080520701	0.11
CCL27	-7.1889	-7.9362	-7.6711	0.378869401	0.10855

BAG3	-0.1545	-0.3232	-0.3468	0.104877659	0.10795
TBR1	0.0765	-0.2939	-0.2163	0.195341479	0.1076
TSPYL1	0.0353	0.096	-0.0418	0.06906246	0.10745
KAZN	-1.5207	-1.1084	-1.4218	0.215248717	0.10725
AHSA1	0.0358	-0.1643	-0.1713	0.117600609	0.10705
RPA2	0.5021	0.7176	0.5036	0.123988239	0.10625
OLA2_BOLA2	-0.7978	-0.6952	-0.8519	0.079591101	0.1054
PCBP2	0.2227	0.2377	0.1249	0.061255857	0.1053
CEP43	4.6946	4.6038	4.5442	0.075737441	0.105
MAEA	1.3972	1.3534	1.2704	0.064401967	0.1049
SNCA	-2.4229	-2.0629	-2.3467	0.189714206	0.1038
OMD	-6.7993	-6.8229	-6.9145	0.060853047	0.1034
RBFOX3	2.8153	2.8125	2.7105	0.059714432	0.1034
GGH	1.1398	1.2222	1.0777	0.072487263	0.1033
IGFBP2	3.1883	3.1642	3.0734	0.060590786	0.10285
HLA-A	-0.7378	-0.8276	-0.8853	0.074329873	0.1026
VTI1A	4.4685	4.8277	4.5459	0.189044369	0.1022
LAMA1	-0.4936	-0.8651	-0.7812	0.194835495	0.10185
RAB2B	-0.237	0.3432	-0.0484	0.295959614	0.1015
CFB	-3.1667	-3.0396	-3.2046	0.086425131	0.10145
NRXN3	0.1505	0.1722	0.06	0.059511876	0.10135
SERPINH1	4.3058	4.6055	4.3548	0.160764621	0.10085
IMPACT	-0.8498	-0.898	-0.9745	0.062882934	0.1006
TADA3	1.0165	0.927	0.8714	0.073207035	0.10035
PPP1CC	-0.2432	0.3776	-0.0318	0.315618694	0.099
ST13	0.6954	0.8815	0.6895	0.109187927	0.09895
FLI1	-0.7676	-0.2719	-0.6178	0.254232617	0.09805
KIF1C	0.9208	0.9197	0.8222	0.056611866	0.09805
GRP	-6.6126	-7.022	-6.9148	0.21229894	0.0975
SATB1	1.2893	1.2633	1.1791	0.057604514	0.0972
KIR2DL2	-5.3156	-3.9444	-4.7272	0.687892899	0.0972
BRAP	0.9291	1.1681	0.9518	0.131922945	0.0968
HS1BP3	-1.9329	-2.2307	-2.1782	0.15896183	0.0964
MFAP3L	-1.1154	-0.464	-0.8858	0.33039203	0.0961
FSTL1	-2.7813	-2.6911	-2.8319	0.071322086	0.0957
CST1	-3.1358	-2.7161	-3.0215	0.21697993	0.09555
ATP6V1D	0.5559	0.5089	0.4374	0.059670624	0.095
PI3	-5.4139	-4.9621	-5.2827	0.232422403	0.0947
EDEM2	0.2442	-0.2646	-0.1041	0.260112341	0.0939
EHBP1	4.167	4.0707	4.0258	0.072142382	0.09305
GDF2	-2.512	-2.178	-2.4365	0.175156359	0.0915
FGF19	-5.9203	-5.5559	-5.8296	0.189703954	0.0915
AP3S2	0.4606	0.3414	0.31	0.079451201	0.091
KIAA0319	-2.6412	-2.5083	-2.6656	0.084657211	0.09085
TG	3.8457	3.9549	3.8112	0.075016198	0.0891
IL17F	-0.6159	-0.9513	-0.8715	0.175210616	0.0879
SPINK6	-0.046	0.0783	-0.071	0.079964555	0.08715
HSD11B1	-2.7278	-2.7754	-2.8383	0.055426257	0.0867
CD99	2.3405	2.3881	2.278	0.055217781	0.0863
HSD17B3	0.7965	0.6276	0.6258	0.098038207	0.08625

INSL3	-3.5345	-3.518	-3.6123	0.050361328	0.08605
BLOC1S3	-0.0696	-0.0456	-0.1435	0.051025516	0.0859
PI16	-2.3743	-2.1044	-2.3245	0.143625706	0.08515
NPC2	3.4839	3.4198	3.368	0.058058677	0.08385
TCP11	-1.365	-1.4718	-1.5021	0.072019372	0.0837
ITPA	2.1619	1.7572	1.8762	0.207993421	0.08335
ADGRB3	-4.053	-4.0975	-4.1584	0.052912223	0.08315
OFD1	0.2412	-0.5899	-0.2574	0.418307188	0.08305
SERPINA5	-3.9946	-3.5384	-3.8494	0.233067401	0.0829
VASN	-3.5857	-3.7641	-3.7577	0.101202372	0.0828
PDE4D	-0.796	-1.0256	-0.9932	0.124267024	0.0824
MMUT	0.4143	0.9951	0.6225	0.294252341	0.0822
LPA	-6.6795	-6.5431	-6.6934	0.08305446	0.0821
UGDH	0.0595	-1.079	-0.5917	0.571212888	0.08195
PLXDC2	-3.2289	-2.5761	-2.9837	0.329749562	0.0812
SNX5	0.7162	0.3894	0.4717	0.169976361	0.0811
HLA-DRA	-2.2868	-2.371	-2.4097	0.062838072	0.0808
EDDM3B	-1.52	-1.672	-1.6767	0.089144994	0.0807
ITGBL1	1.3132	1.2948	1.2233	0.047491789	0.0807
TPSAB1	0.5244	0.6011	0.4822	0.060278437	0.08055
CALB1	1.0221	1.1435	1.0024	0.076414724	0.0804
IL18R1	-6.4913	-7.1392	-6.8953	0.327230199	0.08005
FGFR2	-1.3355	-1.3682	-1.4312	0.048642882	0.07935
RECK	-5.1016	-5.2817	-5.2707	0.100955287	0.07905
CCL13	-8.1956	-9.1056	-8.7293	0.457263123	0.0787
GAGE2A	0.5229	0.6275	0.4968	0.069167502	0.0784
MNDA	-3.1866	-3.5255	-3.4342	0.175354241	0.07815
MTHFSD	-0.4102	0.0146	-0.2745	0.216967102	0.0767
CC2D1A	1.2013	0.8637	0.9558	0.1745119	0.0767
TGFBR1	1.6026	1.4911	1.4708	0.070964287	0.07605
CHCHD6	0.2839	0.4064	0.2695	0.075227677	0.07565
LDLRAP1	-0.5431	0.1087	-0.2924	0.328779293	0.0752
TRIAP1	3.2175	3.3152	3.1918	0.065106758	0.07455
CLEC2L	0.4735	0.2901	0.3088	0.100921867	0.073
GTF2IRD1	-1.007	-0.7393	-0.9455	0.140216487	0.07235
DNAJC21	0.2997	0.2005	0.1779	0.064790226	0.0722
PRDX6	-1.5306	-1.6088	-1.6412	0.056858538	0.0715
VSIG4	-4.0475	-4.0776	-4.1337	0.043748638	0.07115
TBC1D17	0.8887	0.5207	0.6336	0.188523394	0.0711
SCPEP1	-2.4734	-3.2196	-2.9154	0.375214623	0.0689
SELENOP	3.8709	3.8415	3.7874	0.042354496	0.0688
ADGRV1	-0.859	-0.0639	-0.5302	0.399526624	0.06875
XCL1	-4.3654	-4.0242	-4.2634	0.175137127	0.0686
TIA1	1.0267	0.9141	0.902	0.068769252	0.0684
SKAP1	-2.4466	-2.5929	-2.5881	0.083115362	0.06835
CD5L	-3.5193	-3.4175	-3.5363	0.064246504	0.0679
SUSD1	-3.0956	-4.182	-3.7067	0.544612746	0.0679
BAP18	-1.5539	-1.8122	-1.7507	0.134926758	0.06765
CNTNAP2	-3.303	-2.9738	-3.2058	0.169137262	0.0674
ENPP2	-3.3222	-3.4317	-3.4442	0.067119918	0.06725

NFATC1	-1.2489	-1.0814	-1.2321	0.092239706	0.06695
BCR	-0.9286	-1.0786	-1.0699	0.084203503	0.0663
OMP	1.2207	1.4152	1.2519	0.104459386	0.06605
PSMD5	-0.6071	-1.029	-0.8839	0.214348571	0.06585
STAB2	-2.6872	-2.7416	-2.78	0.046629318	0.0656
LILRB4	-1.2206	-1.8193	-1.5854	0.301725576	0.06545
MUC13	-1.6619	-2.3086	-2.0503	0.325523768	0.06505
TGFB2	1.5201	0.9738	1.182	0.275711969	0.06495
SELPLG	-2.505	-2.6517	-2.643	0.082300851	0.06465
ADAM22	-7.1089	-7.1639	-7.2009	0.046292548	0.0645
PSMD1	2.1816	2.3903	2.2217	0.110747205	0.06425
FGF16	-2.5282	-2.3072	-2.4816	0.116496009	0.0639
IL3	4.7684	4.7648	4.7029	0.036821235	0.0637
MRPL58	0.1223	-0.0138	-0.0091	0.077256348	0.06335
PLCB2	-0.5492	-0.4075	-0.5417	0.079733703	0.06335
IMPA1	-0.1428	-0.27	-0.2696	0.073323757	0.0632
FCRL1	-7.0661	-6.8906	-7.0415	0.095022997	0.06315
PRC1	0.1825	-0.0091	0.024	0.102411279	0.0627
ENO3	-1.341	-1.7145	-1.5904	0.19022067	0.06265
ALMS1	-0.1396	-0.1606	-0.2122	0.037359336	0.0621
MMP9	-2.4365	-2.7707	-2.6656	0.170891028	0.062
NGFR	3.111	3.0906	3.0388	0.037220604	0.062
HJV	-2.8618	-3.2867	-3.1351	0.215335188	0.06085
HPGDS	-0.9938	-0.9717	-1.0434	0.036718433	0.06065
PTPRN2	-5.1237	-5.1962	-5.2201	0.050200299	0.06015
ANGPT1	-1.2249	-0.8989	-1.1215	0.166592477	0.0596
SIGLEC10	-2.6558	-2.7826	-2.7785	0.072053614	0.0593
REG3G	-0.7618	-1.7117	-1.2956	0.476163778	0.05885
MGMT	-0.5427	-0.935	-0.7976	0.199061155	0.05875
WNT9A	-1.4355	-1.4911	-1.5219	0.043789192	0.0586
USO1	0.388	1.0952	0.6831	0.355209389	0.0585
CHAD	-5.2312	-4.9311	-5.1391	0.153734847	0.05795
NEFL	-2.073	-1.6178	-1.9032	0.230033418	0.0578
AKT2	3.4716	3.3177	3.3371	0.083817083	0.05755
SERPINA3	-1.4028	-1.2321	-1.3738	0.091340371	0.05635
MYO6	1.3897	1.1663	1.2219	0.116301161	0.0561
CHAC2	0.1789	0.5987	0.3332	0.212340442	0.0556
IL7	0.3554	0.7604	0.503	0.204965656	0.0549
EDAR	-3.4274	-3.5045	-3.5205	0.049779547	0.05455
IL6ST	-1.1418	-1.14	-1.1952	0.031363036	0.0543
COMMD9	0.3952	0.3829	0.3348	0.031919325	0.05425
WASF1	-0.0035	0.0825	-0.0123	0.052377603	0.0518
NPDC1	-3.7285	-4.4258	-4.1287	0.349918024	0.05155
PADI4	-1.6079	-1.5229	-1.6169	0.051868423	0.0515
ATF4	0.4698	0.2971	0.3321	0.091297663	0.05135
DHRS4L2	0.9166	0.5626	0.6884	0.179451423	0.0512
BTN3A2	-3.1063	-2.8904	-3.0492	0.111870952	0.05085
SNCG	-4.0936	-5.2259	-4.7106	0.56691069	0.05085
RBP2	-3.0252	-2.9309	-3.0288	0.055512551	0.05075
TMED8	-0.5943	-0.4103	-0.5526	0.096474677	0.0503

TPMT	4.3763	4.6376	4.4568	0.133819891	0.05015
NPTX1	5.042	5.1182	5.0313	0.047385898	0.0488
MTSS1	5.3173	5.284	5.2521	0.032602505	0.04855
SERPINE2	0.23	0.2226	0.1781	0.028073178	0.0482
AMDHD2	1.1868	0.9745	1.0326	0.109715192	0.04805
COL9A1	6.979	6.9957	6.9393	0.028971078	0.04805
IL12A_IL12B	-7.2604	-7.5803	-7.4655	0.162060205	0.04515
VWA5A	-0.5592	-1.6093	-1.129	0.525685289	0.04475
SERPINI2	-2.0885	-2.0149	-2.0964	0.044947414	0.0447
STXBP3	0.5481	0.3486	0.4039	0.102998366	0.04445
ANKMY2	1.1748	1.1087	1.0975	0.041773077	0.04425
IL17RB	-4.1121	-3.2546	-3.7275	0.429507047	0.04415
SH3GLB2	-2.4329	-2.3395	-2.4302	0.053162236	0.044
CLSPN	0.8387	0.6243	0.6878	0.11012903	0.0437
CRYGD	-3.8844	-3.9343	-3.953	0.035462797	0.04365
TMPRSS15	-3.6753	-2.9699	-3.3662	0.353597148	0.0436
TREM2	-8.4787	-8.4109	-8.4883	0.042189572	0.0435
MYBPC2	-1.8234	-2.5508	-2.2305	0.364562125	0.0434
IFNW1	-0.3631	-0.3835	-0.4165	0.026946614	0.0432
MAPK13	-0.0256	-0.4353	-0.2735	0.206352344	0.04305
STX1B	-0.5128	-0.3479	-0.473	0.086048494	0.04265
METAP1	0.7833	0.6513	0.6749	0.07039356	0.0424
GSTA1	-0.9422	-0.8921	-0.9584	0.034564288	0.04125
ADGRD1	-6.8316	-6.261	-6.5862	0.28622851	0.0399
DENND2B	0.7324	0.0387	0.3465	0.347581966	0.03905
PRTG	-5.8057	-6.4613	-6.1725	0.328572427	0.039
YAP1	2.3506	2.4939	2.3836	0.075044409	0.03865
GUCA2A	-7.3781	-7.8994	-7.677	0.261583849	0.03825
EVI2B	-0.589	-0.3081	-0.4865	0.14214876	0.03795
IL2	-0.4819	1.4886	0.4663	0.985482181	0.03705
MIF	5.1253	5.1527	5.102	0.025377615	0.037
PZP	-1.7186	-1.4819	-1.6369	0.120226716	0.03665
DDAH1	1.2486	1.1733	1.1744	0.043160437	0.03655
CALCA	-4.2717	-4.6533	-4.4988	0.191947571	0.0363
C5	-1.0205	-1.0044	-1.0478	0.021939538	0.03535
SOX9	3.3346	3.1529	3.2085	0.093101611	0.03525
HADH	-1.5133	-1.8744	-1.7286	0.181661287	0.03475
PECR	-0.1087	-0.072	-0.1236	0.026556418	0.03325
PYDC1	-1.0775	-1.4604	-1.3017	0.192381453	0.03275
UBAC1	-1.2439	-1.2556	-1.2825	0.019792507	0.03275
GNPDA1	0.6295	0.4108	0.4875	0.110962892	0.03265
EIF5	0.8652	0.35	0.575	0.258286688	0.0326
ANGPTL2	-5.7484	-6.7307	-6.2721	0.4915094	0.03255
MSLNL	-0.7944	-0.2984	-0.5789	0.248708832	0.0325
AFP	-2.8234	-2.7933	-2.8401	0.023717574	0.03175
ITPR1	-0.8766	-0.5978	-0.7685	0.140566437	0.0313
KLK10	-5.5372	-5.7018	-5.6504	0.084211401	0.0309
DNMBP	1.4001	1.4007	1.3697	0.017727192	0.0307
DTD1	3.7155	3.7983	3.7264	0.044989369	0.0305
SHPK	0.1634	0.2897	0.1966	0.065474601	0.02995

FGD3	-0.3332	-0.8723	-0.6325	0.270096693	0.02975
PRAME	0.2613	0.4838	0.3429	0.112559333	0.02965
FAM20A	0.263	0.1602	0.182	0.054166533	0.0296
ADAMTS13	-7.2381	-7.4113	-7.3541	0.088247833	0.0294
CASP7	0.4977	0.5008	0.4703	0.016786006	0.02895
RAD51	1.4953	1.4458	1.4432	0.02935819	0.02735
PMCH	-3.3026	-3.7616	-3.5589	0.230021006	0.0268
CD248	0.3332	0.3882	0.3342	0.031469562	0.0265
CPQ	-0.341	-0.3346	-0.3637	0.015291937	0.0259
CGN	0.1939	-0.1261	0.008	0.160697241	0.0259
SOD3	-2.5288	-2.5605	-2.57	0.021573827	0.02535
EIF2AK3	-0.3139	-0.3813	-0.3729	0.036729461	0.0253
SCGB3A1	-1.2346	-1.1309	-1.2073	0.053752426	0.02455
PPIB	5.4762	5.4752	5.452	0.013692334	0.0237
GID8	0.4524	-0.2036	0.1011	0.328275743	0.0233
SMC3	-0.6742	-1.0738	-0.8968	0.200233164	0.0228
IL3RA	0.1578	0.1142	0.1134	0.025406561	0.0226
ESM1	-6.5136	-6.7031	-6.6301	0.095578502	0.02175
LMOD2	-0.5154	0.1246	-0.2163	0.320227424	0.0209
RNASET2	1.8066	1.6972	1.731	0.056015117	0.0209
TXNDC9	-0.827	-0.7598	-0.8137	0.035585437	0.0203
VSNL1	-0.5907	-0.9018	-0.7665	0.15598875	0.02025
CASC3	0.2403	0.264	0.232	0.016606123	0.02015
LHB	-2.0038	-2.0539	-2.048	0.027381441	0.01915
MAPKAPK2	-0.9569	-0.8283	-0.9112	0.065190567	0.0186
IFNLR1	-4.5511	-5.4156	-5.0014	0.432375604	0.01805
GNPDA2	2.7427	2.8237	2.7653	0.041797767	0.0179
ESPL1	0.1793	0.0298	0.0867	0.075457074	0.01785
DRG2	0.289	0.2711	0.2625	0.013519246	0.01755
EIF2S2	0.5881	0.0822	0.3178	0.253148264	0.01735
PVALB	-4.101	-3.5747	-3.8548	0.263331901	0.01695
CA11	-0.6929	-0.5379	-0.632	0.078090354	0.0166
FES	1.5322	1.7095	1.6049	0.089127007	0.01595
MAN2B2	-2.6888	-2.9138	-2.8169	0.112859957	0.0156
PNLIPRP2	-9.5863	-9.4947	-9.556	0.046666083	0.0155
COL4A4	0.1175	0.615	0.3509	0.248907821	0.01535
SLC51B	-2.4557	-2.6168	-2.5515	0.081029768	0.01525
TNN	3.2541	3.3056	3.265	0.027139823	0.01485
LTA	-3.7011	-3.3629	-3.5468	0.16931575	0.0148
CA8	0.6195	0.4418	0.5162	0.089240817	0.01445
CCL17	-3.4879	-3.222	-3.3692	0.133204317	0.01425
F11	-3.3515	-3.471	-3.4251	0.060282695	0.01385
SOST	-10.7951	-9.405	-10.1134	0.695092735	0.01335
EGFLAM	-2.6367	-2.5512	-2.6069	0.043398886	0.01295
NXPH1	-0.2392	-0.467	-0.3659	0.114139491	0.0128
KLK4	-2.3261	-2.2703	-2.311	0.028862144	0.0128
XPNPEP2	-6.9372	-7.2649	-7.1133	0.164002571	0.01225
RTKN2	-0.5182	-1.0677	-0.8047	0.274833738	0.01175
IL17RA	-0.4785	-0.712	-0.6067	0.116937006	0.01145
IFI30	-2.3244	-1.8628	-2.1045	0.23088578	0.0109

SLURP1	-1.3647	-1.4146	-1.4004	0.025710374	0.01075
PPP1R12B	-1.5552	-1.5887	-1.5827	0.017862904	0.01075
NPL	-0.6311	-0.8908	-0.7713	0.129987422	0.01035
LIFR	-6.2712	-6.3224	-6.3069	0.02625573	0.0101
IL22	-1.5423	-1.5994	-1.5808	0.029122214	0.00995
DCTN6	-1.3346	-0.6811	-1.0173	0.326795548	0.00945
AMN	-0.6442	-0.8785	-0.7706	0.117271665	0.00925
MPI	-2.4176	-2.2185	-2.3272	0.09969007	0.00915
SYNGAP1	-0.5295	-0.0459	-0.2966	0.241854591	0.0089
COCH	-1.3126	-1.243	-1.2865	0.035160631	0.0087
HAVCR1	-6.9954	-6.6259	-6.8186	0.184807007	0.00795
NUP50	0.4648	0.1433	0.2976	0.160793128	0.00645
RGL2	-1.0442	-0.7584	-0.9074	0.142943392	0.0061
MKI67	2.4321	2.4166	2.4183	0.008500784	0.00605
ARMCX2	-0.7566	-1.0594	-0.914	0.151439625	0.006
ALDH2	-0.4256	-0.6363	-0.5367	0.105402293	0.00575
MATN3	-3.5938	-3.5846	-3.5946	0.005556978	0.0054
CPOX	0.0788	0.3176	0.1929	0.119439204	0.0053
CD209	-5.4413	-5.8005	-5.6262	0.179626065	0.0053
DNAJB2	3.1356	3.2338	3.1795	0.0491917	0.0052
VWC2L	-2.6197	-2.1024	-2.3662	0.25866709	0.00515
CDH5	-5.565	-5.8714	-5.7226	0.15322106	0.0044
REXO2	1.0115	0.8723	0.9377	0.069642229	0.0042
NID1	1.8433	2.0505	1.943	0.103624466	0.0039
TRAF3IP2	0.4073	0.3229	0.3615	0.042251154	0.0036
MAP2K6	2.2015	1.8574	2.026	0.17206153	0.00345
ORM1	-0.9866	-0.8574	-0.9254	0.064629818	0.0034
CD40LG	-5.2542	-5.0509	-5.1548	0.1016583	0.00225
MUCL3	-0.048	-0.1446	-0.0985	0.048316698	0.0022
KDM3A	0.3605	0.2609	0.3088	0.04981208	0.0019
STK11	1.4758	1.4935	1.4832	0.008889507	0.00145
PHLDB2	-1.7937	-1.8501	-1.8223	0.028200946	0.0004
HSPB6	-2.1242	-2.0822	-2.1035	0.021000714	0.0003
SAT2	0.4352	0.5795	0.5071	0.072150144	0.00025
NEXN	6.2861	6.2113	6.2485	0.037400178	0.0002
B3GAT3	0.6692	0.4035	0.537	0.13285053	-0.00065
ROBO1	-5.0824	-4.8397	-4.96	0.121351514	-0.00105
PPP1R14D	-0.1683	-0.3635	-0.2646	0.097602886	-0.0013
BAG6	-0.7388	-0.9372	-0.8365	0.09920378	-0.0015
KRT8	-0.0095	0.4492	0.222	0.229353359	-0.00215
GFOD2	-2.0786	-1.9965	-2.0351	0.041074363	-0.00245
AOC1	-0.9714	-1.2995	-1.1328	0.164057134	-0.00265
FCAMR	-5.7548	-5.9976	-5.8734	0.121410763	-0.0028
XG	-5.6836	-5.7438	-5.7099	0.03017985	-0.0038
SDK2	-3.3076	-2.6372	-2.9684	0.335207955	-0.004
CHIT1	-7.0669	-7.5651	-7.3119	0.249111247	-0.0041
NUDT15	1.1423	1.0132	1.0819	0.064594453	-0.00415
CCNE1	1.0146	1.0688	1.046	0.027213477	-0.0043
TEK	-4.8474	-4.9166	-4.8773	0.034706243	-0.0047
SPON2	-5.6388	-6.0595	-5.8444	0.210367876	-0.00475

AGER	-7.0577	-7.4019	-7.225	0.172122311	-0.0048
CD80	-1.5373	-1.1615	-1.3445	0.187921296	-0.0049
IRAG2	1.8314	1.7778	1.81	0.026980734	-0.0054
PRKAB1	-0.3396	-0.5414	-0.4349	0.100951787	-0.0056
KRT6C	1.1987	0.7578	0.9852	0.220486515	-0.00695
LUZP2	-5.9703	-4.2511	-5.1035	0.859610051	-0.0072
USP28	2.6974	2.8017	2.7568	0.052317715	-0.00725
SLC4A1	-0.2727	-0.2597	-0.2583	0.007940613	-0.0079
CRISP3	-3.8306	-3.7283	-3.7715	0.051355526	-0.00795
LAMTOR5	-0.0232	-0.3698	-0.1884	0.173363087	-0.0081
CRTAM	-5.2428	-5.7209	-5.4736	0.239097449	-0.00825
IGSF21	-2.8059	-3.0279	-2.9086	0.11110339	-0.0083
ANGPTL1	-0.1216	-0.2484	-0.1765	0.063589648	-0.0085
NCAM2	-5.1526	-5.1729	-5.1542	0.011286718	-0.00855
IFIT3	-0.2493	-0.1343	-0.1828	0.057734305	-0.009
RPGR	-0.0457	-0.2662	-0.1463	0.110390685	-0.00965
SLC28A1	-0.7654	-0.5935	-0.6698	0.086130386	-0.00965
ITIH3	-1.8662	-2.0952	-1.971	0.114636876	-0.0097
SERPINB9	-2.2077	-2.4652	-2.3266	0.128875534	-0.00985
BGLAP	-2.4492	-2.4456	-2.4373	0.006102732	-0.0101
PKLR	-0.6494	-0.4886	-0.5588	0.080615383	-0.0102
APOC1	-1.5686	-1.5811	-1.5645	0.008646965	-0.01035
NME3	0.9372	1.0938	1.0265	0.078557134	-0.011
SPINK1	-1.5268	-1.7562	-1.6302	0.114885392	-0.0113
PTPRK	-1.4857	-1.494	-1.4785	0.007756503	-0.01135
PCBD1	5.1953	5.3964	5.3073	0.100767075	-0.01145
CA6	-3.2607	-3.3534	-3.2956	0.046819049	-0.01145
CPB1	-3.8599	-4.409	-4.1228	0.274632379	-0.01165
MCTS1	0.4547	0.3431	0.4117	0.05628724	-0.0128
PCNA	-0.7214	-0.758	-0.7268	0.01975753	-0.0129
SELE	-8.1921	-7.9813	-8.0738	0.105662813	-0.0129
JMJD1C	-2.0528	-1.638	-1.8319	0.207546404	-0.0135
ACE2	-2.9757	-2.8033	-2.8757	0.08656743	-0.0138
SERPINA11	-4.0882	-4.3781	-4.2191	0.1451768	-0.01405
MPO	-4.9053	-4.8373	-4.8567	0.035029321	-0.0146
IL12RB1	-0.3612	-0.4198	-0.3757	0.03052054	-0.0148
FNTA	-0.4085	-0.4124	-0.3956	0.008792611	-0.01485
AHSG	-6.92	-6.4379	-6.664	0.241204484	-0.01495
EP300	0.4037	0.4102	0.422	0.009277033	-0.01505
PLXNB3	-1.486	-1.7046	-1.58	0.109656372	-0.0153
PTGDS	-4.3411	-3.9408	-4.1253	0.200353845	-0.01565
COL2A1	6.2298	6.1818	6.2218	0.025716402	-0.016
PRG2	5.8379	5.8175	5.8439	0.013839075	-0.0162
REG3A	-7.8288	-7.7921	-7.7937	0.020742308	-0.01675
AK1	0.0167	-0.0116	0.0194	0.017171585	-0.01685
IPCEF1	-2.1972	-0.8109	-1.4863	0.693225752	-0.01775
NFASC	-0.0135	-0.1175	-0.0468	0.053108976	-0.0187
GRK5	0.5151	0.704	0.6284	0.095074935	-0.01885
SPRR1B	-1.251	-0.0895	-0.6511	0.580855234	-0.01915
RRM2	4.2629	4.3435	4.3226	0.041827543	-0.0194

PTH	-5.548	-3.6727	-4.5901	0.937722886	-0.02025
STAU1	-0.4016	-0.3356	-0.3483	0.035019471	-0.0203
MRPS16	0.3328	0.0431	0.2085	0.145335096	-0.02055
CNTN4	-3.0357	-3.313	-3.1538	0.13915671	-0.02055
ESAM	-2.6479	-2.7493	-2.6779	0.052089538	-0.0207
TSPAN1	-0.1236	-0.1279	-0.105	0.012171415	-0.02075
CRLF1	-3.1028	-3.049	-3.055	0.029482424	-0.0209
DBI	6.7109	6.8535	6.8033	0.07233321	-0.0211
CFD	2.4355	2.5802	2.5293	0.073402248	-0.02145
ATP6V1G1	2.8314	2.7728	2.8239	0.031888922	-0.0218
CRTAC1	-6.0693	-6.1905	-6.1073	0.061988816	-0.0226
GPHA2	0.1756	0.2348	0.2278	0.032348313	-0.0226
BRD2	-0.398	-0.6239	-0.4879	0.113731277	-0.02305
RARRES2	-7.5322	-7.3351	-7.4097	0.099515342	-0.02395
DAAM1	-0.1378	0.227	0.0694	0.182961125	-0.0248
PCDH12	-2.0953	-2.1206	-2.0829	0.019214318	-0.02505
SULT1A1	-3.5854	-3.7881	-3.6612	0.102417886	-0.02555
PIKFYVE	1.6475	1.486	1.5929	0.082149275	-0.02615
DPEP1	-4.0491	-4.3963	-4.1964	0.1742628	-0.0263
GPD1	-3.3203	-3.4305	-3.3482	0.057294182	-0.0272
CES1	-6.2492	-6.3007	-6.2477	0.030175873	-0.02725
IL10RA	-0.8516	-0.7471	-0.772	0.054583911	-0.02735
ATP1B3	-0.0075	-0.4126	-0.1826	0.203169068	-0.02745
KCNH2	-1.2175	-1.547	-1.3547	0.165516052	-0.02755
SIL1	-1.8297	-1.9574	-1.8655	0.065871769	-0.02805
GADD45B	-0.1168	0.2193	0.0797	0.168850832	-0.02845
NXPH3	-4.3627	-3.899	-4.1023	0.232435202	-0.02855
ITGAM	-0.9486	-1.1878	-1.0393	0.120758285	-0.0289
CDH6	1.9163	1.681	1.8286	0.118913932	-0.02995
ACHE	-1.3251	-1.0441	-1.1546	0.14156359	-0.03
DCN	-4.2387	-5.3724	-4.7754	0.56711721	-0.03015
CCL20	-5.6854	-5.1772	-5.4011	0.254697513	-0.0302
BST2	1.3842	1.5001	1.4727	0.060574775	-0.03055
C1QBP	1.4579	1.4928	1.5063	0.024976055	-0.03095
DCBLD2	-4.8836	-4.775	-4.798	0.057228082	-0.0313
GMPR	-0.8255	-0.5378	-0.6502	0.14499146	-0.03145
COL1A1	0.018	0.053	0.0672	0.025322191	-0.0317
CRNN	-6.6179	-6.5485	-6.551	0.039366272	-0.0322
EPGN	0.156	0.393	0.307	0.119976387	-0.0325
GDF15	4.7964	4.8046	4.833	0.019206596	-0.0325
CPXM2	-3.09	-3.1447	-3.0847	0.033216913	-0.03265
TTR	-5.3392	-5.1471	-5.2103	0.097904596	-0.03285
CA4	-3.467	-3.8311	-3.6159	0.183053298	-0.03315
DDX39A	0.8223	0.9371	0.9134	0.060607948	-0.0337
SMAD5	-4.6716	-4.184	-4.3937	0.244593629	-0.0341
MAMDC2	-3.4233	-3.4993	-3.4269	0.042877189	-0.0344
SEMA3F	-2.7177	-3.2487	-2.9488	0.266241814	-0.0344
WFIKKN2	-2.1396	-1.9918	-2.0312	0.076537311	-0.0345
IL18RAP	-5.3129	-5.4255	-5.3347	0.059719678	-0.0345
COL5A1	6.0715	6.2114	6.1761	0.07275445	-0.03465

TGFBR2	-4.1575	-3.8424	-3.9647	0.158859026	-0.03525
CAPN3	-0.7734	-0.833	-0.7678	0.036135301	-0.0354
MAMDC4	-2.4413	-2.41	-2.3899	0.025902574	-0.03575
PAK4	-0.6248	-0.4103	-0.4816	0.109239935	-0.03595
NTF4	0.1694	0.3162	0.2789	0.076301791	-0.0361
ATRN	1.2488	1.3172	1.3192	0.040080585	-0.0362
AAMDC	-0.1888	-0.4254	-0.2696	0.120264874	-0.0375
NELL2	2.5248	2.617	2.6086	0.050980127	-0.0377
TREH	-2.0384	-2.2253	-2.0939	0.095984217	-0.03795
RBPMS	3.4634	3.5595	3.5498	0.052905986	-0.03835
EDA2R	1.6834	1.8828	1.8217	0.10216038	-0.0386
TSPAN7	0.1238	-0.587	-0.1929	0.356101657	-0.0387
COMT	-1.5175	-1.8506	-1.645	0.168069043	-0.03905
SERPINF2	-1.2234	-1.1718	-1.1576	0.034626194	-0.04
BAX	5.8247	5.528	5.7172	0.15021306	-0.04085
FIS1	3.4847	3.6092	3.5881	0.066629598	-0.04115
RABEPK	0.4786	0.273	0.4171	0.105529159	-0.0413
CPB2	-1.8897	-1.4271	-1.6163	0.23257363	-0.0421
MYLPF	-0.6837	-1.1207	-0.8598	0.219867013	-0.0424
GP1BA	-6.5235	-6.7755	-6.6069	0.128378036	-0.0426
FSHB	-0.4308	-0.5551	-0.4497	0.066978678	-0.04325
AGXT	-1.3176	-1.4278	-1.3294	0.060505978	-0.0433
HGFAC	-6.2576	-7.2791	-6.7248	0.51136852	-0.04355
CLTA	1.909	1.6043	1.8002	0.154410891	-0.04355
CLINT1	-0.3706	-0.0678	-0.1745	0.153583821	-0.0447
MAP2K1	2.0453	2.0354	2.0853	0.026419753	-0.04495
ATP1B1	-0.165	-0.1929	-0.1336	0.02966721	-0.04535
CRTAP	0.54	0.4935	0.5621	0.035015758	-0.04535
PRDX2	4.2839	4.373	4.3751	0.052058717	-0.04665
ZNRF4	-0.3316	-0.4836	-0.3602	0.080776977	-0.0474
SCARF2	-2.1036	-2.5553	-2.2817	0.227526357	-0.04775
GAD2	0.3154	0.1661	0.2888	0.079638077	-0.04805
PDCL2	-0.0062	-0.0209	0.0346	0.028754652	-0.04815
ACE	-0.4234	-0.4354	-0.3811	0.0285242	-0.0483
PGLYRP2	-3.01	-2.6258	-2.7689	0.194171943	-0.049
SSNA1	1.4297	1.3658	1.4477	0.04304033	-0.04995
CDC25A	-0.0608	-0.0416	-0.0006	0.030750827	-0.0506
SMOC2	-2.6992	-2.4907	-2.5439	0.108336344	-0.05105
SAP18	-0.7997	0.2612	-0.2181	0.531271409	-0.05115
KLHL41	-1.129	-1.6146	-1.3201	0.244627887	-0.0517
IL31RA	-2.6558	-1.6049	-2.0786	0.526298765	-0.05175
CLUL1	-4.8218	-5.476	-5.097	0.328469603	-0.0519
VAV3	1.1921	1.2028	1.2495	0.030523597	-0.05205
MTIF3	-0.061	-0.4771	-0.2167	0.210233941	-0.05235
MAP2	4.2955	4.2622	4.3313	0.034557537	-0.05245
NAPRT	-0.1314	-0.4155	-0.221	0.145241879	-0.05245
INSL4	-2.1213	-2.0557	-2.0355	0.044857255	-0.053
C7	-2.5815	-2.301	-2.3882	0.143555436	-0.05305
UHRF2	0.8268	0.1037	0.5186	0.362859674	-0.05335
NOTCH3	-6.6596	-6.3923	-6.4725	0.137166407	-0.05345

VPS28	-0.2231	-0.2664	-0.1913	0.037696463	-0.05345
SUMF1	-0.6356	-0.7646	-0.6461	0.071639724	-0.054
LGMN	-2.5322	-2.4643	-2.4441	0.046152031	-0.05415
SERPINA7	-0.8076	-0.73	-0.7144	0.049918868	-0.0544
AREG	-0.9734	-0.9854	-0.9248	0.032089251	-0.0546
GC	-3.0497	-2.7096	-2.825	0.172952431	-0.05465
MRC1	-4.1259	-4.5147	-4.2652	0.196985693	-0.0551
PTPRH	-3.1743	-2.5372	-2.8005	0.320143129	-0.05525
VCAM1	-4.3079	-4.2857	-4.2407	0.034238575	-0.0561
SIAE	-1.2471	-1.1392	-1.1367	0.063030178	-0.05645
ADRA2A	-0.3721	-0.4493	-0.354	0.050612153	-0.0567
HBQ1	-1.4966	-1.2837	-1.3332	0.111412312	-0.05695
LTA4H	-1.0803	-1.0134	-0.9898	0.046944684	-0.05705
NBL1	-4.811	-5.0572	-4.8762	0.127558144	-0.0579
IL9	-1.9793	-0.9323	-1.3972	0.52459213	-0.0586
EIF1AX	0.3056	0.4309	0.427	0.071242848	-0.05875
TXNL1	1.2068	1.2975	1.3111	0.056700882	-0.05895
PTPRC	-2.167	-2.8873	-2.4679	0.361770936	-0.05925
POSTN	1.6978	1.655	1.7358	0.040423755	-0.0594
F9	-1.955	-1.7089	-1.7719	0.12784093	-0.06005
RELB	-2.3825	-2.0784	-2.1703	0.155965413	-0.06015
RNASE6	0.1304	0.1702	0.2107	0.040150509	-0.0604
SCN2A	-0.2691	-0.3644	-0.2561	0.059132591	-0.06065
OPHN1	-0.8771	-0.9375	-0.846	0.046525298	-0.0613
LBP	-5.0359	-3.5738	-4.2431	0.731918796	-0.06175
PRSS53	-0.3816	-0.3754	-0.3163	0.036044741	-0.0622
LSM1	0.5642	0.2682	0.4785	0.15230812	-0.0623
CBLN4	0.5001	0.5557	0.5907	0.045688657	-0.0628
F12	-1.1648	-1.1972	-1.1178	0.039923093	-0.0632
DLGAP5	2.139	2.1441	2.2051	0.036779115	-0.06355
PRUNE2	0.1807	0.0686	0.1884	0.067054381	-0.06375
FZD8	-0.4689	-0.3926	-0.3669	0.053050573	-0.06385
HSD17B14	1.9076	1.8372	1.9364	0.051033061	-0.064
CTNNA1	-0.0845	-0.2597	-0.1078	0.095141596	-0.0643
ABHD14B	1.2082	1.3555	1.3468	0.082646779	-0.06495
EREG	0.9794	0.6862	0.8978	0.151327107	-0.065
DGCR6	-0.0706	0.3523	0.2059	0.214759408	-0.06505
ALDH3A1	0.2786	0.0267	0.2182	0.131513003	-0.06555
CLEC3B	-0.7636	-0.6278	-0.6301	0.077748719	-0.0656
SLC39A14	-3.7273	-4.1741	-3.885	0.226597418	-0.0657
KLK7	-1.587	-1.4704	-1.4628	0.06961676	-0.0659
LDLR	0.7331	0.7717	0.8186	0.042817092	-0.0662
ISM2	0.411	0.5383	0.5409	0.074258625	-0.06625
SERPINC1	-2.4955	-2.1897	-2.2758	0.157689008	-0.0668
RANBP2	0.6406	0.3469	0.5607	0.151851978	-0.06695
CD3D	-0.0235	0.2405	0.1756	0.13756745	-0.0671
FHIP2A	-0.005	-0.4732	-0.172	0.237283824	-0.0671
BTD	0.5534	0.6176	0.6527	0.050355635	-0.0672
PDZD2	0.5862	0.0304	0.3755	0.280595242	-0.0672
GAMT	0.4295	0.0271	0.2957	0.204928508	-0.0674

SNAPIN	0.9539	0.939	1.014	0.039705205	-0.06755
KIR3DL2	-0.8972	-0.8397	-0.8004	0.048684323	-0.06805
C1S	-3.5807	-3.4228	-3.4336	0.088211356	-0.06815
OXT	1.8002	1.7379	1.8374	0.050274878	-0.06835
MMP15	0.1327	0.0692	0.1695	0.050738841	-0.06855
ABRAXAS2	0.2507	-0.3637	0.0127	0.30978711	-0.0692
PLA2G2A	-5.4876	-5.3123	-5.3302	0.096458333	-0.06975
DLK1	-3.6473	-3.7643	-3.636	0.071037056	-0.0698
GAST	-2.4139	-2.5065	-2.3904	0.06138162	-0.0698
CSF2	1.0354	0.9751	1.0763	0.050908971	-0.07105
SHBG	-5.968	-6.4784	-6.1519	0.258498749	-0.0713
GBP4	-0.2507	-0.1337	-0.1205	0.071665054	-0.0717
RPS10	0.6543	0.0592	0.4296	0.300507975	-0.07285
ROBO2	-1.5003	-1.7363	-1.5454	0.125281563	-0.0729
CAMKK1	-0.3626	-0.027	-0.1218	0.173012061	-0.073
SERPINF1	0.433	0.4778	0.5287	0.047882391	-0.0733
NDUFS6	1.2171	1.0843	1.2242	0.078801713	-0.0735
PDGFRA	-3.0651	-3.4592	-3.1882	0.201622345	-0.07395
JAM3	0.9782	1.0224	1.0745	0.048203976	-0.0742
GP2	-0.3321	-0.3636	-0.273	0.045995326	-0.07485
CPLX2	0.2505	0.1906	0.2954	0.052578608	-0.07485
ACSL1	0.1071	-0.2499	0.004	0.183731607	-0.0754
FABP3	-2.1659	-1.6345	-1.8239	0.269327038	-0.0763
TF	-3.0345	-2.7202	-2.8003	0.163324901	-0.07705
BABAM1	0.6495	0.3316	0.5676	0.165057576	-0.07705
F13B	-2.2338	-2.1776	-2.1282	0.052836477	-0.0775
HEPH	-4.6054	-4.4762	-4.463	0.078681468	-0.0778
CLEC7A	-4.2441	-4.2164	-4.1523	0.047087401	-0.07795
MMP10	-4.3264	-4.3073	-4.2389	0.046006558	-0.07795
KIAA1549L	0.5855	0.985	0.8632	0.204757084	-0.07795
ITIH4	-2.8603	-2.7102	-2.7068	0.087658257	-0.07845
C3	-6.0567	-5.8749	-5.8871	0.101623685	-0.0787
MMP3	-4.4708	-4.6368	-4.4747	0.094734383	-0.0791
CRELD1	-4.5884	-4.9069	-4.6682	0.165724661	-0.07945
BRD1	0.6975	0.3687	0.6126	0.17068717	-0.0795
PDE5A	1.3173	1.3406	1.4087	0.047494666	-0.07975
PIK3IP1	-2.7263	-2.9758	-2.7708	0.133076106	-0.08025
ID4	-0.9531	-1.495	-1.1437	0.274892603	-0.08035
ART3	1.1843	1.3165	1.3309	0.080804043	-0.0805
COL4A1	-3.5617	-3.5323	-3.4662	0.048911178	-0.0808
SULT2A1	-2.164	-2.1592	-2.0807	0.046769256	-0.0809
TIE1	-9.6026	-8.6348	-9.037	0.486193555	-0.0817
VWF	-6.4732	-6.3355	-6.3226	0.083474607	-0.08175
FXYD5	0.5845	-0.4219	0.1632	0.505416769	-0.0819
SORCS2	-2.3479	-1.5534	-1.8684	0.400078222	-0.08225
SMTN	0.2306	0.3091	0.3528	0.061920352	-0.08295
LILRA5	-7.0162	-6.7212	-6.7847	0.155268316	-0.084
WFDC12	-5.3699	-5.1057	-5.1526	0.140961307	-0.0852
MOG	-4.7169	-4.8412	-4.6938	0.079278896	-0.08525
DMD	3.3195	3.3494	3.4199	0.051550008	-0.08545

TAFA5	-3.9548	-3.9184	-3.8511	0.052611691	-0.0855
MFAP3	1.7956	1.4861	1.7265	0.162458826	-0.08565
IL7R	-3.7544	-3.7457	-3.6639	0.049928582	-0.08615
PPP1R12A	4.6014	4.6407	4.7075	0.053640687	-0.08645
PM20D1	-5.8807	-5.8504	-5.7787	0.052381581	-0.08685
NPHS2	-0.8006	-1.2052	-0.9149	0.208582414	-0.088
PCDH7	-4.6741	-4.6635	-4.5801	0.051484496	-0.0887
MMP13	6.3655	6.1797	6.3621	0.106303779	-0.0895
SEMA7A	5.316	5.3146	5.4052	0.051908509	-0.0899
CPTP	0.2472	0.4439	0.4359	0.11132728	-0.09035
INSL5	-4.224	-4.7187	-4.3805	0.252850278	-0.09085
RNASE4	-2.6725	-2.4147	-2.4527	0.139174279	-0.0909
C1QTNF5	0.4987	0.5105	0.596	0.053098619	-0.0914
ROBO4	-1.6022	-2.2219	-1.8202	0.314355155	-0.09185
GFRA3	-7.4052	-6.7285	-6.975	0.342480457	-0.09185
MASP1	-2.1042	-2.152	-2.0362	0.058192898	-0.0919
CD27	-5.3509	-5.1203	-5.1437	0.126922391	-0.0919
FLT3LG	-4.0388	-3.9611	-3.907	0.066251214	-0.09295
SKIV2L	0.3332	-0.2926	0.1141	0.317551922	-0.0938
ICAM4	-4.1387	-4.0343	-3.9923	0.075383818	-0.0942
GH2	-5.0783	-5.8087	-5.3493	0.369227464	-0.0942
SPRED2	0.879	0.4188	0.7432	0.236453322	-0.0943
SIGLEC1	-3.9575	-3.809	-3.7888	0.092123088	-0.09445
SCN3B	0.2413	0.0941	0.2627	0.091789397	-0.095
TXK	-1.1616	-0.6051	-0.788	0.283643444	-0.09535
LRRN1	-4.7178	-4.4522	-4.4895	0.143791249	-0.0955
APOB	-0.0783	-0.2023	-0.0447	0.083008755	-0.0956
OMG	-0.7424	-0.7564	-0.6534	0.055865911	-0.096
C2orf69	0.802	0.2997	0.6469	0.257199384	-0.09605
ASGR2	-2.0349	-1.8549	-1.8488	0.105727969	-0.0961
GGT5	-5.5003	-4.1627	-4.7349	0.67112142	-0.0966
ZBTB16	1.7825	2.1414	2.0587	0.187942784	-0.09675
CD1C	-2.1898	-2.2674	-2.1312	0.068320519	-0.0974
SFTPA2	-4.3385	-5.1644	-4.654	0.416765166	-0.09745
DOC2B	0.1935	0.4332	0.4109	0.132423651	-0.09755
RBPMS2	1.4932	1.5025	1.5958	0.056742312	-0.09795
PLAUR	3.595	3.696	3.7439	0.076011644	-0.0984
ATP2B4	0.4388	0.0921	0.3639	0.182430873	-0.09845
BCL2	-1.4318	-1.3714	-1.3028	0.064543422	-0.0988
PTPN9	0.9276	0.8688	0.9974	0.064378361	-0.0992
SIT1	-1.3684	-1.3422	-1.256	0.058808503	-0.0993
CD33	-3.066	-3.0454	-2.9563	0.058305603	-0.0994
LCN2	-4.9576	-4.538	-4.6483	0.217522696	-0.0995
GUCY2C	-2.2523	-1.9587	-2.0054	0.15776642	-0.1001
TNFRSF4	-6.6916	-6.8662	-6.6786	0.10475998	-0.1003
CD48	0.8859	1.0962	1.0916	0.120110879	-0.10055
NEDD4L	0.9643	0.8295	0.9976	0.089010805	-0.1007
TACSTD2	-3.5185	-3.3507	-3.333	0.10237218	-0.1016
ARNTL	0.5034	0.6714	0.6898	0.102719294	-0.1024
PGM2	1.0962	0.545	0.923	0.281869852	-0.1024

PMVK	-3.0906	-2.9816	-2.9335	0.080493292	-0.1026
ITGA6	-1.4812	-1.527	-1.4011	0.063723962	-0.103
CFI	-0.9652	-0.7726	-0.7656	0.113272474	-0.1033
PTPRR	-1.9883	-1.9045	-1.8421	0.073360571	-0.1043
RTN4R	-5.4781	-5.6827	-5.4755	0.118883528	-0.1049
FKBP1B	-0.6959	-0.5484	-0.5169	0.095559318	-0.10525
SPTLC1	0.1844	0.1424	0.2688	0.064374322	-0.1054
HLA-E	-0.0054	-0.3261	-0.0597	0.17164204	-0.10605
IL24	1.6249	1.2811	1.5592	0.182507781	-0.1062
ENPEP	-0.4131	-0.4201	-0.3103	0.061472053	-0.1063
ARHGEF10	0.2046	0.3546	0.3868	0.097239978	-0.1072
PSMA1	-0.7276	-1.8277	-1.1699	0.553556703	-0.10775
SLAMF6	-1.824	-1.6238	-1.616	0.11790171	-0.1079
ASS1	-0.2773	-0.5002	-0.2808	0.127693004	-0.10795
PBXIP1	-3.5963	-3.4071	-3.3937	0.113301192	-0.108
NCK2	3.5744	3.657	3.7238	0.074839116	-0.1081
UPK3A	0.0888	-0.2353	0.0351	0.173705047	-0.10835
CALY	0.2416	-0.1789	0.1407	0.21952419	-0.10935
SERPINA1	-0.8949	-0.575	-0.6254	0.172001172	-0.10955
STK24	0.3852	0.9234	0.765	0.276585683	-0.1107
EPHX2	0.2797	0.1155	0.3087	0.104186435	-0.1111
CXCL1	-2.2786	-2.0344	-2.0454	0.137923215	-0.1111
SAA4	-0.8807	-0.626	-0.6422	0.142604804	-0.11115
DAB2	-1.8729	-1.7059	-1.6779	0.105434024	-0.1115
SELP	-4.8509	-5.0978	-4.8625	0.139319932	-0.11185
NELL1	-3.4189	-3.203	-3.1987	0.125909584	-0.11225
PTPRS	-1.1518	-1.1998	-1.0626	0.069623368	-0.1132
DEFB116	-0.6805	-0.8581	-0.6561	0.110258121	-0.1132
RAC3	-0.908	-1.4206	-1.0511	0.264501613	-0.1132
AOC3	-4.6824	-4.5507	-4.5033	0.092797683	-0.11325
GORASP2	0.3566	0.481	0.5323	0.090348898	-0.1135
CDH17	-3.6368	-3.5873	-3.4983	0.0701825	-0.11375
EPHA1	-4.4055	-4.7443	-4.4603	0.181862805	-0.1146
CD36	-0.6648	-0.9196	-0.6775	0.143583158	-0.1147
MANSC4	-0.9242	-1.9228	-1.3085	0.503695169	-0.115
MRPL52	-0.3005	-0.5644	-0.3173	0.147751966	-0.11515
NGF	-0.0829	-0.0715	0.0383	0.066927125	-0.1155
MLLT1	-0.0534	0.2992	0.2384	0.188489894	-0.1155
SIRT5	0.247	-0.0629	0.2078	0.168746921	-0.11575
BCAN	-3.8259	-4.4207	-4.0068	0.304911206	-0.1165
DSG2	-4.1516	-4.5141	-4.216	0.193398216	-0.11685
SLIT2	-2.863	-1.0483	-1.8381	0.909884621	-0.11755
KLK3	-1.3883	-1.4925	-1.3228	0.0855823	-0.1176
VSTM2B	-1.6935	-1.3975	-1.4279	0.162830955	-0.1176
S100G	0.9676	0.999	1.1014	0.069969231	-0.1181
CNTN5	0.1683	0.2198	0.3122	0.072912299	-0.11815
BPIFB2	-4.3643	-4.4593	-4.2934	0.083241236	-0.1184
TARBP2	2.4279	2.2369	2.451	0.11751129	-0.1186
TIMP2	-2.9534	-2.8818	-2.799	0.077267673	-0.1186
FOSB	1.5692	1.4762	1.6414	0.082817953	-0.1187

SCGB3A2	-5.6478	-5.4732	-5.4418	0.110985825	-0.1187
SCN2B	0.5939	0.4425	0.6373	0.102268079	-0.1191
MYL3	-2.2813	-2.6032	-2.3231	0.175034692	-0.11915
WFIKKN1	-3.227	-3.4714	-3.2299	0.140274742	-0.1193
GP6	-5.3697	-5.6448	-5.3879	0.153844543	-0.11935
PDCD1LG2	-3.1709	-3.7462	-3.3389	0.295828605	-0.11965
CACNA1C	0.3353	0.1563	0.3662	0.113323887	-0.1204
ZNF830	0.1382	0.2847	0.3319	0.101003119	-0.12045
MME	-5.8598	-5.2071	-5.4129	0.333689112	-0.12055
KLKB1	-0.9251	-0.7186	-0.7003	0.124841353	-0.12155
AP2B1	-0.119	-0.0747	0.0248	0.07364462	-0.12165
SMOC1	-1.8546	-1.5735	-1.5923	0.157147457	-0.12175
FGF2	-0.0197	-0.0793	0.0726	0.076534371	-0.1221
ADAMTSL4	-2.2976	-2.5546	-2.304	0.146566435	-0.1221
NLGN2	-0.4908	-0.1766	-0.2113	0.172262387	-0.1224
SCN3A	0.1278	-0.4309	-0.0291	0.288156942	-0.12245
A_AMY1B_A	-2.8764	-3.0177	-2.8244	0.100028813	-0.12265
TNFRSF6B	-5.9088	-6.0941	-5.8787	0.116647089	-0.12275
THRAP3	-0.8035	-0.1865	-0.3707	0.316737136	-0.1243
PRR4	-0.5489	-0.3092	-0.3045	0.13976739	-0.12455
PNMA1	0.848	0.0167	0.5577	0.421903382	-0.12535
PFDN2	2.9089	2.4028	2.7825	0.263402816	-0.12665
S100P	-2.4731	-2.9199	-2.5698	0.235071315	-0.1267
CD276	-0.2639	-0.163	-0.0866	0.088931678	-0.12685
TNFRSF13B	-8.2963	-8.3477	-8.1945	0.077969481	-0.1275
ATP6V1F	3.2311	3.3126	3.3999	0.084416606	-0.12805
FZD10	-0.5137	-0.3218	-0.2897	0.121128045	-0.12805
NRGN	4.892	4.8683	5.0083	0.07493039	-0.12815
MUC2	0.0505	0.0023	0.1555	0.078335262	-0.1291
CSDE1	1.5121	1.4291	1.6001	0.085512182	-0.1295
MRPL46	0.1864	-0.0312	0.2072	0.132046053	-0.1296
NCAM1	0.3939	0.4718	0.5636	0.084944825	-0.13075
ARHGAP30	-0.4796	-0.2753	-0.2463	0.1271537	-0.13115
SUSD5	-1.5287	-1.2885	-1.2774	0.141992335	-0.1312
POF1B	-1.456	-0.565	-0.8793	0.451893863	-0.1312
SERPING1	-2.8931	-2.7012	-2.6659	0.122264427	-0.13125
IGLC2	-0.5954	-0.5271	-0.4299	0.083169486	-0.13135
IL13RA2	0.012	-0.0425	0.1163	0.080690954	-0.13155
WFDC2	-6.2566	-6.1271	-6.0603	0.09980496	-0.13155
BMPER	-4.9782	-5.1037	-4.9092	0.098608232	-0.13175
IDI2	-0.4301	-0.6295	-0.398	0.125421303	-0.1318
CCL22	-6.5642	-6.5198	-6.4095	0.079655027	-0.1325
VGF	-2.1608	-2.4033	-2.1493	0.143442497	-0.13275
ECHDC3	-2.3972	-2.7347	-2.4321	0.18560308	-0.13385
AFM	-1.9892	-1.7056	-1.7133	0.161559617	-0.1341
ICAM2	-6.1041	-6.0467	-5.9412	0.082625077	-0.1342
AMY2A	-6.0911	-6.5813	-6.202	0.257054903	-0.1342
CASP10	0.5542	0.5158	0.6704	0.080496542	-0.1354
FABP6	-1.312	-1.9223	-1.4815	0.315039939	-0.13565
NPTX2	1.4248	1.6702	1.6833	0.145610794	-0.1358

KIF22	0.4367	-0.4501	0.1292	0.450288607	-0.1359
ASPN	-1.7603	-1.9653	-1.7262	0.129329437	-0.1366
PLIN1	-0.923	-1.2288	-0.9391	0.172094422	-0.1368
SCRG1	1.6639	1.7063	1.8221	0.081888786	-0.137
HMGCL	-0.1167	-0.2153	-0.0289	0.093252131	-0.1371
SLITRK2	-6.8284	-6.837	-6.6946	0.079847939	-0.1381
LYVE1	-1.3196	-1.3446	-1.1936	0.08093413	-0.1385
CSPG5	-0.1627	0.1029	0.1091	0.155164987	-0.139
OSBPL2	0.4027	0.1843	0.4327	0.13558584	-0.1392
RLN1	-1.7681	-2.0312	-1.76	0.154292288	-0.13965
ST3GAL1	-4.0843	-3.7384	-3.7708	0.191040493	-0.14055
IL36A	0.277	0.0281	0.2932	0.148599944	-0.14065
TNFRSF1B	-3.3065	-3.5844	-3.3039	0.161201437	-0.14155
CD2	-0.6514	-0.7703	-0.5681	0.101620979	-0.14275
UBE2Z	-0.0257	-0.2517	0.0042	0.139913557	-0.1429
CD74	-4.1448	-4.0721	-3.9652	0.090341076	-0.14325
CFHR4	0.0302	0.0669	0.1926	0.085167619	-0.14405
GSTA3	-2.1546	-2.1595	-2.0129	0.083261095	-0.14415
ERBB3	-6.1875	-5.9774	-5.9381	0.134093786	-0.14435
ANXA5	3.2276	2.9954	3.2563	0.143067199	-0.1448
PRKG1	4.8601	4.9297	5.0402	0.090820721	-0.1453
HDAC8	0.6175	0.7496	0.8289	0.106793305	-0.14535
MMP12	-7.6481	-7.4731	-7.415	0.121337147	-0.1456
CDK5RAP3	-0.0767	-0.1048	0.0555	0.085598423	-0.14625
PRND	-3.7884	-3.7787	-3.6362	0.0852107	-0.14735
ENPP5	-1.5382	-1.6757	-1.4592	0.109559345	-0.14775
NTRK2	-2.6725	-3.0082	-2.6924	0.18833487	-0.14795
TNFRSF10C	-7.5211	-6.9907	-7.1075	0.278696777	-0.1484
PON3	-8.7336	-7.8407	-8.1382	0.454656981	-0.14895
PALM2	1.7125	1.9387	1.9748	0.142168292	-0.1492
IL1R1	-4.9224	-4.533	-4.5785	0.212904446	-0.1492
FABP1	-8.824	-8.1416	-8.3333	0.351948183	-0.1495
GRIK2	-1.2056	-1.0785	-0.9924	0.107255039	-0.14965
NPPB	-0.2589	-0.8828	-0.4209	0.323740333	-0.14995
DPP4	-6.5047	-6.782	-6.4931	0.163550736	-0.15025
A1BG	-2.1364	-1.849	-1.8424	0.167868163	-0.1503
IL34	-2.312	-2.9102	-2.4587	0.311773524	-0.1524
CDHR2	-2.8526	-2.9152	-2.7314	0.093443958	-0.1525
AMFR	1.6366	1.1905	1.5663	0.239851669	-0.15275
CST3	2.6912	2.8183	2.9077	0.108795695	-0.15295
FGF21	-1.8309	-1.7133	-1.6184	0.106451883	-0.1537
PCDH9	-2.3881	-2.8201	-2.4494	0.233737809	-0.1547
IGF2BP3	0.2449	0.3043	0.4299	0.094453445	-0.1553
ITGAX	-1.9655	0.0095	-0.8222	0.991588354	-0.1558
IL12B	-8.6309	-8.6444	-8.4812	0.090578309	-0.15645
SERPINA4	-6.0179	-5.4999	-5.6017	0.274441761	-0.1572
LEPR	-2.6891	-3.1289	-2.7517	0.237915475	-0.1573
GNGT1	0.7075	0.4118	0.7174	0.173650924	-0.15775
CES2	-3.2495	-3.2526	-3.0931	0.091205647	-0.15795
CD300A	-1.0124	-0.9263	-0.8103	0.101417964	-0.15905

COQ7	-0.4655	-0.9367	-0.5417	0.252936382	-0.1594
POMC	-2.1101	-1.9944	-1.8923	0.108970745	-0.15995
GSAP	-1.666	-1.3433	-1.3445	0.18596549	-0.16015
C1GALT1C1	0.0609	-0.4256	-0.0216	0.260353766	-0.16075
AZU1	-6.22	-5.9392	-5.9183	0.168477664	-0.1613
PGLYRP1	-8.3275	-8.1951	-8.0998	0.114352627	-0.1615
APOA1	-2.9475	-2.5906	-2.6074	0.201381835	-0.16165
GAD1	0.2582	0.4236	0.5027	0.124762588	-0.1618
MDK	-0.5372	-0.5769	-0.3949	0.095698642	-0.16215
CTSF	-3.6765	-3.3549	-3.3535	0.186081308	-0.1622
DCLRE1C	0.6363	-0.6163	0.1724	0.633279514	-0.1624
INHBC	-5.977	-6.7161	-6.1836	0.381337257	-0.16295
L6-OID2091	-1.4892	-1.3677	-1.265	0.112231294	-0.16345
SERPIND1	-4.5333	-4.4897	-4.3471	0.097387679	-0.1644
CRYM	-0.247	0.3455	0.2139	0.311127953	-0.16465
UMOD	-7.1494	-6.4227	-6.6213	0.375593854	-0.16475
THBS4	-5.7934	-5.8785	-5.671	0.104307254	-0.16495
EPN1	-0.1411	-0.2893	-0.0496	0.120962515	-0.1656
AMBN	0.5335	0.6541	0.7596	0.113134006	-0.1658
FCRLB	-2.4244	-2.4835	-2.2878	0.100375013	-0.16615
ADAMTSL2	-2.1478	-1.9175	-1.8661	0.15001941	-0.16655
SPINK2	-7.1074	-6.5554	-6.6648	0.292280436	-0.1666
LPP	0.5966	-0.5171	0.207	0.565160252	-0.16725
PRSS8	-7.7496	-8.059	-7.7364	0.182562026	-0.1679
IL21R	-0.4726	-0.2857	-0.2106	0.134917024	-0.16855
CLEC5A	-6.1378	-4.9803	-5.3901	0.586912484	-0.16895
F2	-0.8589	-0.8256	-0.6732	0.09901106	-0.16905
CYTL1	0.8763	0.932	1.0737	0.101774375	-0.16955
LEG1	-6.6886	-6.6274	-6.4884	0.102588563	-0.1696
BPIFB1	-8.8684	-9.2742	-8.9013	0.225392421	-0.17
L6-OID2010	-0.9349	-1.0062	-0.7994	0.105047751	-0.17115
NAA10	0.944	1.3906	1.3391	0.244338502	-0.1718
PVR	-1.2937	-1.6079	-1.2787	0.185884946	-0.1721
CREBZF	0.5574	0.1674	0.535	0.218986879	-0.1726
FGF12	-0.6145	-0.7346	-0.5018	0.1164196	-0.17275
TDO2	1.1312	0.8161	1.1465	0.186496765	-0.17285
VCPKMT	-0.6719	-0.8138	-0.5698	0.122539803	-0.17305
CD163	-5.4387	-5.7596	-5.4236	0.189780935	-0.17555
DCUN1D1	0.4846	0.417	0.6268	0.10708769	-0.176
NDUFB7	-0.1891	-0.2627	-0.0498	0.108126361	-0.1761
ECE1	-1.8188	-1.7736	-1.6199	0.104265638	-0.1763
CST7	4.0465	4.1281	4.2636	0.109659488	-0.1763
ENOX2	1.399	0.9602	1.3566	0.242031733	-0.177
MAN1A2	-4.3573	-4.455	-4.2289	0.11339684	-0.17725
EXTL1	-2.2361	-2.7925	-2.337	0.296434934	-0.1773
RGMB	-1.4466	-1.829	-1.4597	0.217095931	-0.1781
ITM2A	-2.2714	-2.462	-2.1882	0.140366805	-0.1785
HCG22	-0.5231	-0.91	-0.5373	0.219392548	-0.17925
ANGPTL7	-5.4908	-4.9466	-5.0388	0.291249744	-0.1799
NOTCH1	-0.167	-0.1655	0.0137	0.103896888	-0.17995

PCDH1	-3.6852	-3.5201	-3.4218	0.133104257	-0.18085
HBZ	-1.4404	-1.5375	-1.3077	0.115358673	-0.18125
FGF23	-3.7324	-4.0009	-3.6853	0.170251784	-0.18135
CXCL14	-0.0891	-0.2125	0.0311	0.121803503	-0.1819
GHR	-2.7522	-2.5927	-2.4896	0.132305593	-0.18285
CXCL13	-5.1826	-5.3184	-5.0676	0.125543671	-0.1829
TFAP2A	0.3983	0.8257	0.7949	0.238366301	-0.1829
GIPC2	1.5242	1.4599	1.6757	0.110797398	-0.18365
INSR	-0.3955	-0.2947	-0.1613	0.117477544	-0.1838
PLA2G10	-5.2502	-5.4943	-5.1883	0.161788143	-0.18395
HRG	-4.5608	-4.5425	-4.3673	0.106827103	-0.18435
IL1RL2	-4.7963	-6.0082	-5.2174	0.615276558	-0.18485
FAP	-3.3118	-3.2786	-3.1096	0.108434373	-0.1856
ABO	-1.9187	-1.861	-1.7031	0.111613216	-0.18675
IFNAR1	-4.3445	-4.9655	-4.4682	0.328696917	-0.1868
RIPK4	-1.0189	-1.2931	-0.9691	0.17447143	-0.1869
RAB37	-0.574	0.4784	0.1392	0.537161776	-0.187
RNASE10	-1.4888	-1.5396	-1.3253	0.111980192	-0.1889
LRIG3	-0.5208	-0.6744	-0.4081	0.133672448	-0.1895
NPY	-4.6429	-4.7106	-4.487	0.114662592	-0.18975
MANSC1	-4.0844	-4.721	-4.2129	0.336634673	-0.1898
HRC	-3.3236	-3.6936	-3.3179	0.215283913	-0.1907
SIRPB1	-3.4971	-3.9446	-3.5292	0.249614309	-0.19165
IL1RAP	-4.7939	-4.7111	-4.5603	0.118438057	-0.1922
FSTL3	-5.3343	-5.0352	-4.9923	0.186308534	-0.19245
RAB11FIP3	-0.4739	-1.2689	-0.6784	0.412822702	-0.193
IL20	0.4052	0.0065	0.3991	0.228448995	-0.19325
SARG	-2.1933	-1.8941	-1.8498	0.186849039	-0.1939
TPPP2	0.4759	0.5882	0.7261	0.125318089	-0.19405
CTLA4	0.7004	0.661	0.8752	0.114009532	-0.1945
SNX18	0.5851	0.6236	0.7989	0.11396109	-0.19455
VIPR1	1.0303	0.7902	1.105	0.164482593	-0.19475
SORBS1	0.7224	0.6882	0.9003	0.113874536	-0.195
HDAC9	-0.5999	-0.7747	-0.4922	0.142571958	-0.1951
SOD1	5.2203	5.3939	5.5025	0.14234217	-0.1954
ERVV-1	-0.0425	-0.0485	0.15	0.112911839	-0.1955
CFC1	-1.2287	-1.1028	-0.9701	0.1293149	-0.19565
MEP1A	-3.4999	-3.376	-3.2416	0.129185564	-0.19635
CEACAM6	-1.9651	-1.9912	-1.7803	0.114971779	-0.19785
ADA2	-7.0175	-6.8453	-6.7335	0.143066465	-0.1979
LILRA6	-4.3913	-4.3699	-4.1826	0.114815025	-0.198
ARNT	-0.1646	-0.0019	0.1149	0.140376743	-0.19815
SSH3	-0.6098	-0.5902	-0.4016	0.114964748	-0.1984
SMPD1	-3.2605	-3.3303	-3.0963	0.12013165	-0.1991
SLC12A2	0.5911	0.3497	0.6696	0.166719435	-0.1992
RBM25	-0.0921	-0.0037	0.1513	0.123209253	-0.1992
TST	0.0315	0.2532	0.3416	0.159753696	-0.19925
TSHB	-6.979	-6.9868	-6.7836	0.115131982	-0.1993
ULBP2	-1.1396	-1.9026	-1.3205	0.398692492	-0.2006
NFE2	1.2663	1.0872	1.3776	0.146513173	-0.20085

SDC4	-2.056	-2.0989	-1.8764	0.118041532	-0.20105
ELN	-1.8873	-2.3479	-1.916	0.258041876	-0.2016
RICTOR	-0.2307	-0.3934	-0.1104	0.142028389	-0.20165
IL17C	-4.7082	-4.0863	-4.195	0.332151958	-0.20225
HAO1	-4.9967	-4.6391	-4.6147	0.21385241	-0.2032
BHMT2	0.9254	0.3397	0.8363	0.31559332	-0.20375
PAMR1	-5.866	-6.1028	-5.7801	0.167126868	-0.2043
FRZB	-4.1188	-4.2666	-3.9884	0.139190661	-0.2043
NUDT10	0.838	0.5513	0.8996	0.185878249	-0.20495
OLFM4	-0.7765	-0.8837	-0.6251	0.129928031	-0.205
OGN	-5.4286	-5.2336	-5.1257	0.153522973	-0.2054
COLEC12	-5.4364	-5.5449	-5.2841	0.131011564	-0.20655
SCARF1	-5.1536	-5.0099	-4.8748	0.139422105	-0.20695
EFA1_DEFA1	-1.4953	-1.73	-1.4054	0.16759637	-0.20725
ENOPH1	0.2499	0.2057	0.4351	0.121708011	-0.2073
ACAA1	0.0814	0.0904	0.2942	0.120346223	-0.2083
MED21	0.6273	0.1617	0.6028	0.262028249	-0.2083
PRL	-6.8785	-8.2014	-7.331	0.672361116	-0.20895
PRTN3	-5.8285	-6.0131	-5.711	0.152286911	-0.2098
MEGF10	-3.9074	-3.9529	-3.7203	0.123274369	-0.20985
CNDP1	-2.0468	-2.3709	-1.9987	0.202438163	-0.21015
DCDC2C	-0.7685	-0.2131	-0.2802	0.303152453	-0.2106
PRAP1	-4.5092	-4.7725	-4.4302	0.179228523	-0.21065
IL19	-3.9744	-3.9019	-3.7266	0.12740433	-0.21155
AIF1L	1.1993	1.0517	1.338	0.143173054	-0.2125
TCTN3	-3.7368	-3.6979	-3.5044	0.124475714	-0.21295
EIF5A	-0.2029	0.1226	0.1729	0.204004077	-0.21305
JAM2	-3.9154	-3.2671	-3.3781	0.346723997	-0.21315
ENSA	0.55	0.5239	0.7504	0.123924453	-0.21345
TAP1	-2.2563	-1.52	-1.6739	0.38837539	-0.21425
PKD1	-4.1802	-3.6661	-3.7089	0.285264304	-0.21425
MMP8	-4.1959	-4.3688	-4.068	0.150959962	-0.21435
REN	-1.9104	-1.984	-1.7326	0.129248959	-0.2146
TNFRSF1A	0.2568	0.2842	0.4852	0.124711881	-0.2147
SMARCA2	-6.1632	-5.6278	-5.6806	0.295054729	-0.2149
PCDHB15	-2.5555	-3.5569	-2.8409	0.515899073	-0.2153
MLN	-2.5696	-2.1365	-2.1372	0.249848574	-0.21585
GAL	-6.7851	-6.6128	-6.4829	0.151594932	-0.21605
SERPINA6	-2.9656	-2.8268	-2.6801	0.142768215	-0.2161
CTSO	-6.343	-6.5807	-6.2455	0.17241741	-0.21635
SMPD3	-0.5216	-0.8134	-0.4508	0.192197225	-0.2167
C1QTNF6	0.2335	-0.1579	0.2556	0.232617218	-0.2178
ANXA10	-3.7427	-2.1509	-2.7285	0.805817457	-0.2183
TREML1	0.3151	0.0405	0.3974	0.186885018	-0.2196
CTSE	-5.4118	-5.4899	-5.2311	0.132746337	-0.21975
ZP3	-3.1501	-3.257	-2.9835	0.137831673	-0.22005
ANGPTL3	-5.5399	-6.1128	-5.6062	0.313383062	-0.22015
NFIC	-0.2966	-0.0774	0.035	0.168642106	-0.222
CCL16	-1.5749	-1.5714	-1.3502	0.128732138	-0.22295
ADH4	-2.0844	-2.132	-1.8849	0.131100737	-0.2233

SLC13A1	0.0605	0.3756	0.4416	0.203666893	-0.22355
COPB2	1.148	0.8899	1.2426	0.182556868	-0.22365
ISM1	-5.7752	-6.0457	-5.6861	0.187270402	-0.22435
TCN1	-0.2908	-0.2901	-0.0654	0.129933149	-0.22505
GZMA	-6.6319	-6.9792	-6.5801	0.217018179	-0.22545
VAMP5	0.2826	-0.0242	0.3551	0.201350019	-0.2259
RGS8	-0.4732	-1.1969	-0.609	0.384666466	-0.22605
PNLIP	-4.5291	-4.1533	-4.1147	0.228926102	-0.2265
ZP4	0.144	-0.5578	0.0196	0.374475046	-0.2265
GCLM	-1.2305	-1.0452	-0.9111	0.160382491	-0.22675
GBP2	-0.8824	-0.4121	-0.4199	0.269304406	-0.22735
IL18BP	-1.7528	-1.8423	-1.5697	0.138952402	-0.22785
BCAT2	0.2204	0.4034	0.5398	0.160265571	-0.2279
GALNT2	-4.107	-3.8125	-3.7315	0.197607144	-0.22825
ITGA11	-4.0321	-4.0829	-3.8289	0.134404167	-0.2286
BST1	-4.3616	-4.5879	-4.2461	0.173867373	-0.22865
ACTN2	-1.2801	-1.1397	-0.9806	0.149847267	-0.2293
CDK1	3.5821	3.5827	3.8123	0.132733166	-0.2299
TNFSF9	1.1035	1.2782	1.4214	0.159209893	-0.23055
KRT5	-1.2281	-1.8451	-1.306	0.336002535	-0.2306
LIPF	-4.2628	-4.9667	-4.3825	0.37662823	-0.23225
IFNL1	-1.2995	-1.8896	-1.3614	0.324305633	-0.23315
CLEC10A	-4.19	-4.2528	-3.9875	0.138644738	-0.2339
PLEKHO1	0.1651	-0.1034	0.265	0.190521574	-0.23415
NRN1	0.3107	0.1837	0.4816	0.149488138	-0.2344
PROC	-7.1994	-7.1387	-6.9344	0.138833221	-0.23465
TNFRSF14	-2.5527	-2.4479	-2.265	0.145606055	-0.2353
EFEMP1	-5.6048	-5.2131	-5.1731	0.238535036	-0.23585
BHLHE40	0.4159	0.7019	0.7948	0.19748064	-0.2359
HMCN2	-7.5298	-7.3757	-7.2164	0.15670719	-0.23635
CLEC1B	-5.3825	-6.9158	-5.9124	0.778739843	-0.23675
SSBP1	0.832	0.4959	0.9009	0.216693109	-0.23695
APOL1	-4.7719	-4.8771	-4.5874	0.146647753	-0.2371
FOLR3	-9.831	-9.7799	-9.5677	0.13962267	-0.23775
CEP152	0.8442	1.4506	1.3853	0.332859895	-0.2379
UNC79	-0.3675	0.3305	0.2196	0.375097592	-0.2381
TNR	-4.7085	-4.1527	-4.1923	0.310092524	-0.2383
RARRES1	-0.9834	-1.0013	-0.754	0.137902175	-0.23835
TYRO3	-6.5314	-6.0982	-6.0764	0.256632838	-0.2384
CEP350	-0.5447	-0.3392	-0.2031	0.171970937	-0.23885
RBP1	-1.4598	-0.5039	-0.7427	0.497494164	-0.23915
OSTN	-0.0338	-0.2218	0.1132	0.167917639	-0.241
SCGB1A1	-4.5866	-4.5021	-4.303	0.145607932	-0.24135
COL3A1	0.7264	0.6046	0.9069	0.152096888	-0.2414
IL22RA1	-0.6572	-1.5071	-0.8407	0.447230593	-0.24145
IGLON5	2.9863	2.8892	3.1794	0.147722747	-0.24165
YOD1	-1.5379	-2.019	-1.5362	0.278255261	-0.24225
ARFIP1	0.7234	0.6408	0.9256	0.146525675	-0.2435
BNIP2	1.6645	1.2712	1.7117	0.241851532	-0.24385
LRRC38	-0.3873	-0.5935	-0.2462	0.174663934	-0.2442

NME1	0.9147	0.7478	1.0763	0.164257126	-0.24505
LYPD1	0.2685	-0.0022	0.3786	0.195963066	-0.24545
RET	-6.2421	-6.2077	-5.9793	0.142836597	-0.2456
DIPK1C	-0.6891	-0.8525	-0.5252	0.163650064	-0.2456
PTH1R	-3.0942	-3.1183	-2.8606	0.142337076	-0.24565
CCER2	-3.2763	-2.4486	-2.6167	0.437496107	-0.24575
SLK	-0.1762	-0.6139	-0.1489	0.260944304	-0.24615
STX5	-0.708	-0.335	-0.2753	0.234493205	-0.2462
MBL2	-3.6982	-3.696	-3.4497	0.142840692	-0.2474
SEZ6L	-4.7529	-5.085	-4.6707	0.219351977	-0.24825
ARHGAP5	-0.5272	-0.4596	-0.2448	0.147455394	-0.2486
CLASP1	0.5758	0.7823	0.9294	0.177629586	-0.25035
GUK1	0.2479	0.2967	0.5247	0.147751864	-0.2524
ATF2	-0.524	-0.5964	-0.3052	0.151609498	-0.255
IL12RB2	-0.1088	-0.4178	-0.0075	0.213732223	-0.2558
MFAP5	-7.3046	-7.0768	-6.9347	0.186597276	-0.256
SERPINI1	-3.652	-3.7147	-3.4271	0.151231092	-0.25625
CASQ2	0.1751	-0.3072	0.1927	0.283673245	-0.25875
CSNK2A1	0.3739	0.0904	0.4914	0.206146994	-0.25925
TIGAR	1.1318	1.3288	1.4896	0.179204948	-0.2593
ICAM3	0.7539	0.8165	1.0449	0.153170014	-0.2597
EPO	-3.0354	-3.207	-2.8614	0.172801389	-0.2598
EDNRB	0.2668	-0.1074	0.3396	0.239838307	-0.2599
RTBDN	-2.952	-3.0419	-2.737	0.156668982	-0.25995
CD83	-6.2073	-5.2999	-5.4936	0.477888087	-0.26
FCGR2B	-4.1551	-3.6896	-3.6618	0.277130529	-0.26055
L6-OID2056	-1.0322	-0.8576	-0.6843	0.173950405	-0.2606
CELA2A	-4.6204	-4.6989	-4.3984	0.15585597	-0.26125
CCT5	-0.8438	-0.5819	-0.4515	0.199789498	-0.26135
VWC2	-5.255	-6.0253	-5.3786	0.413694722	-0.26155
SCG3	-1.724	-1.3933	-1.2961	0.224317015	-0.26255
CASP1	-3.1216	-2.9169	-2.7561	0.183188873	-0.26315
BCL2L1	0.8065	0.8764	1.1059	0.156629403	-0.26445
KRT14	-0.0539	0.0437	0.2595	0.160371943	-0.2646
NPPC	-4.6349	-4.8344	-4.4699	0.182521916	-0.26475
PROCR	0.431	-0.0585	0.4526	0.289050174	-0.26635
IL4	1.8838	1.7208	2.069	0.17421791	-0.2667
MYOM3	-3.3551	-3.2697	-3.0456	0.159845874	-0.2668
CETN2	-0.6213	-0.9272	-0.5074	0.217094473	-0.26685
STAM	-0.0773	0.0457	0.2517	0.166235776	-0.2675
FGFR4	-6.6671	-6.803	-6.4669	0.169072006	-0.26815
ARF6	-2.5012	-2.2185	-2.0914	0.209765639	-0.26845
FCGR3B	-4.0029	-3.8312	-3.6486	0.177177943	-0.26845
CD82	0.1863	-0.3783	0.1725	0.322062168	-0.2685
ATXN2	0.3295	-0.1333	0.3669	0.278622397	-0.2688
HIF1A	0.1483	0.5926	0.6398	0.271171096	-0.26935
COL15A1	-4.7942	-4.5618	-4.408	0.194428496	-0.27
C1RL	0.5113	0.5262	0.7891	0.156264338	-0.27035
PLTP	-6.6674	-6.0434	-6.083	0.349396508	-0.2724
ANXA3	-4.6974	-4.903	-4.5272	0.188177682	-0.273

NRTN	-1.2325	-0.1935	-0.4396	0.54295129	-0.2734
DSC2	-4.2692	-4.1112	-3.9164	0.17671959	-0.2738
TRIM26	0.3383	0.3471	0.6173	0.158601429	-0.2746
GNLY	-3.2849	-2.8311	-2.783	0.276933103	-0.275
ANKRD54	1.2168	1.3265	1.5474	0.168388074	-0.27575
RAPGEF2	-0.6344	-0.1959	-0.1391	0.271056753	-0.27605
TP73	0.4328	-0.3979	0.2956	0.44531423	-0.27815
ANP32C	-0.1954	-0.2625	0.0498	0.164396239	-0.27875
CCL15	-9.2182	-9.1029	-8.8806	0.171602807	-0.27995
SFRP1	-0.3137	-0.3804	-0.0668	0.165203642	-0.28025
NOS2	0.4452	0.0914	0.5486	0.23975607	-0.2803
NFAT5	-0.5328	-0.4474	-0.2092	0.16770478	-0.2809
NMRK2	0.317	0.0175	0.4482	0.220762384	-0.28095
PECAM1	-1.5979	-1.7754	-1.4043	0.185608198	-0.28235
EFNB2	-1.1929	-1.9476	-1.287	0.411262135	-0.28325
DPP10	-2.194	-2.3093	-1.9667	0.174324477	-0.28495
DCUN1D2	0.8202	-0.1916	0.5993	0.531986663	-0.285
IGFL4	-1.4628	-1.5804	-1.2362	0.174952832	-0.2854
CEACAM3	-0.0287	-0.0962	0.2247	0.169186593	-0.28715
CDH23	0.0573	0.1017	0.367	0.167466186	-0.2875
CKB	3.3296	3.2962	3.6006	0.166941067	-0.2877
RPL14	0.0358	-0.146	0.2329	0.189501478	-0.288
CNTN1	-4.408	-4.4993	-4.1655	0.172512985	-0.28815
DRAXIN	-4.4419	-4.1466	-4.0058	0.222564575	-0.28845
NOMO1	-0.7099	-0.4139	-0.2724	0.22325042	-0.2895
ATG4A	0.0312	0.0146	0.3135	0.167983164	-0.2906
CSF1	-1.9064	-1.7865	-1.5557	0.178248488	-0.29075
FLT1	1.8595	1.9011	2.1714	0.169348881	-0.2911
TNFRSF8	-5.1328	-5.3884	-4.9686	0.211551822	-0.292
AMBP	-7.153	-7.0999	-6.8336	0.171148892	-0.29285
FCER2	-2.4092	-2.4299	-2.1257	0.169969791	-0.29385
CRHBP	-9.832	-9.078	-9.1608	0.413497537	-0.2942
FGA	-5.9967	-5.5854	-5.4968	0.266745091	-0.29425
CCN2	-4.0793	-3.9673	-3.7279	0.179507809	-0.2954
SRP14	-1.4572	-1.4633	-1.1638	0.171182661	-0.29645
CEACAM8	-5.1462	-4.1344	-4.3437	0.534096118	-0.2966
OO1-OID3147	-6.0467	-5.5045	-5.4775	0.321117445	-0.2981
KIT	2.1224	2.2666	2.493	0.186813169	-0.2985
FCN2	-8.0845	-7.722	-7.6047	0.250124496	-0.29855
HPSE	-0.4934	-1.0444	-0.4694	0.325269632	-0.2995
CFP	-2.0695	-1.9948	-1.7324	0.177045314	-0.29975
CALCB	-2.7421	-2.8187	-2.4806	0.177276347	-0.2998
ASGR1	-3.8049	-4.5664	-3.8854	0.418354614	-0.30025
IL2RB	0.2509	0.5484	0.6999	0.228421941	-0.30025
ZBP1	-1.9148	-1.9123	-1.6127	0.173700326	-0.30085
TMEM132A	-4.1133	-3.403	-3.4567	0.395502495	-0.30145
CRYBB2	-1.2041	-1.6271	-1.1136	0.274105059	-0.302
IGFBP3	-7.3193	-7.7758	-7.2446	0.287560365	-0.30295
DNAJC6	-2.331	-1.9487	-1.8366	0.259213895	-0.30325
CLSTN3	0.154	0.1565	0.4588	0.175259132	-0.30355

CDH2	-3.4916	-3.5047	-3.1944	0.175492421	-0.30375
SIGLEC9	-6.5705	-7.0724	-6.5175	0.306220678	-0.30395
SFTPD	-5.1804	-6.6972	-5.6336	0.778601036	-0.3052
SLITRK1	-4.0652	-3.6524	-3.5524	0.271835637	-0.3064
CILP	-4.3476	-3.6011	-3.6674	0.413184781	-0.30695
ARID3A	-0.3093	-0.3757	-0.0354	0.180385818	-0.3071
CFH	-7.2997	-7.0482	-6.8662	0.217676557	-0.30775
L6-OID2127(	-2.0401	-2.1056	-1.765	0.180729365	-0.30785
GABARAP	0.4335	0.25	0.65	0.200226746	-0.30825
CD3E	-0.3096	-0.2168	0.0451	0.183945436	-0.3083
DNM3	-1.6234	-2.0694	-1.5358	0.286158092	-0.3106
FLT4	-4.6801	-4.5246	-4.2916	0.195534098	-0.31075
EFNA1	-0.9478	-1.026	-0.6742	0.184722964	-0.3127
CD3G	0.1202	-0.9246	-0.0886	0.552886438	-0.3136
TP53INP1	-0.8331	-0.9105	-0.558	0.185259737	-0.3138
F10	-3.6954	-3.3863	-3.2257	0.238730413	-0.31515
ODAM	-1.8892	-2.4061	-1.832	0.316240483	-0.31565
TYRP1	1.3901	1.4574	1.74	0.185661906	-0.31625
TOP1	-4.3594	-4.0216	-3.8741	0.248790802	-0.3164
NPHS1	-2.8592	-2.3074	-2.2666	0.330989083	-0.3167
NEB	-0.9481	-0.6033	-0.4588	0.251390062	-0.3169
CD300LG	-1.8971	-2.2327	-1.7479	0.248300571	-0.317
TIMD4	-4.845	-4.6771	-4.4428	0.202011444	-0.31825
3103A_DEFB1	-0.2837	-1.177	-0.4118	0.483033046	-0.31855
CD200	-5.4133	-7.1248	-5.9503	0.875314372	-0.31875
USP47	1.0311	0.7525	1.2116	0.231290229	-0.3198
HTR1B	-0.0925	0.2756	0.4114	0.260721582	-0.31985
SPARCL1	-3.7774	-4.127	-3.6322	0.254339091	-0.32
ICOSLG	-5.706	-4.7962	-4.9289	0.491465451	-0.3222
TNFSF14	-5.2926	-5.1982	-4.9227	0.192196783	-0.3227
CSF2RB	-1.0426	-1.856	-1.1265	0.447368048	-0.3228
LRTM1	0.0247	-0.2886	0.1915	0.243746843	-0.32345
SOX2	-0.245	0.0125	0.2073	0.226873158	-0.32355
NPR1	-0.0864	-0.2593	0.1516	0.206307707	-0.32445
ALDH5A1	0.2452	0.0847	0.4903	0.204265195	-0.32535
IL15RA	-0.899	-1.8421	-1.0447	0.507692962	-0.32585
MTPN	2.9273	2.8477	3.2138	0.192547665	-0.3263
IL11	-1.541	-0.8097	-0.8478	0.411658746	-0.32755
TBC1D23	0.1386	0.1948	0.4944	0.191273033	-0.3277
FCER1A	-1.41	-1.7584	-1.2561	0.257349652	-0.3281
MCEE	-0.2042	0.3805	0.4173	0.348685766	-0.32915
MFAP4	-5.2623	-4.9037	-4.7532	0.261542546	-0.3298
KLRB1	-7.998	-7.8844	-7.6111	0.198867401	-0.3301
GIPC3	0.1029	-0.0149	0.3769	0.20102242	-0.3329
GIP	-1.0326	-1.4782	-0.9224	0.294283605	-0.333
PPBP	-4.9479	-5.3798	-4.8305	0.289266561	-0.33335
3104A_DEFB1	-0.9482	-0.0993	-0.188	0.46661957	-0.33575
MEGF11	-0.2635	-0.1857	0.1116	0.197964702	-0.3362
CPXM1	-1.5839	-1.6039	-1.2571	0.194708534	-0.3368
DPEP2	-8.3496	-8.6242	-8.1494	0.238369545	-0.3375

DLL4	-4.131	-3.0225	-3.2389	0.587571786	-0.33785
GZMB	-0.8966	-1.2161	-0.7177	0.252483669	-0.33865
BTC	-2.7098	-2.6282	-2.3301	0.199872568	-0.3389
HYAL1	-1.2573	-0.9587	-0.7688	0.246257392	-0.3392
SPON1	-6.8101	-6.6009	-6.3663	0.22202111	-0.3392
CCL24	-4.1876	-4.139	-3.823	0.197969324	-0.3403
PTPRZ1	-5.5226	-5.257	-5.0492	0.237287364	-0.3406
PALM3	1.8669	1.7793	2.1638	0.201520727	-0.3407
FOLH1	-1.0732	-0.732	-0.5618	0.260421274	-0.3408
RALB	1.2182	0.8182	1.3595	0.280764249	-0.3413
CD38	-6.1014	-6.0187	-5.7184	0.201539235	-0.34165
GAS6	-3.1048	-3.1101	-2.7657	0.197327249	-0.34175
CNTF	-0.1132	0.1972	0.3855	0.25182889	-0.3435
ADGRE1	-3.5862	-4.1527	-3.5257	0.345859196	-0.34375
GLT8D2	0.108	0.2746	0.5353	0.215369969	-0.344
CLEC4M	-6.4552	-6.1993	-5.982	0.236862245	-0.34525
UXS1	-2.2962	-2.3792	-1.9921	0.203802282	-0.3456
ITPRIP	-0.2984	-0.0687	0.1624	0.230400354	-0.34595
JCHAIN	-4.6808	-3.483	-3.7358	0.631355378	-0.3461
CRACR2A	-2.3856	-1.3378	-1.5155	0.560734064	-0.3462
FTCD	-3.1306	-2.9235	-2.6805	0.225288489	-0.34655
CACNA1H	-0.8849	-0.4139	-0.3027	0.30907477	-0.3467
GABRA4	0.0725	0.0411	0.405	0.201645489	-0.3482
TRDMT1	-0.9062	-0.3991	-0.3038	0.323810196	-0.34885
FAM13A	-0.0139	0.327	0.5063	0.264250304	-0.34975
CNPY2	-0.2321	0.036	0.2522	0.242613046	-0.35025
SCG2	-2.5233	-2.5599	-2.1912	0.20312954	-0.3504
CA7	-0.1608	-0.4676	0.0368	0.254162468	-0.351
VTCN1	-0.4889	-0.3043	-0.0449	0.223047648	-0.3517
MAP3K5	1.0988	1.3246	1.5646	0.232936071	-0.3529
ADAMTS16	0.6615	0.2683	0.8179	0.283174669	-0.353
RETN	-7.1824	-7.3759	-6.9249	0.226255571	-0.35425
IOD1-OID305	-3.0652	-2.2975	-2.3265	0.4351019	-0.35485
KLF4	-0.0101	0.0088	0.3549	0.20549429	-0.35555
ACA5_SPACA	-0.241	-0.4537	0.0101	0.232164791	-0.35745
PLCB1	0.1067	-0.1577	0.3327	0.245450443	-0.3582
TNFSF10	-6.6388	-5.848	-5.8842	0.44648558	-0.3592
BRSK2	0.7687	0.2217	0.8554	0.343584434	-0.3602
RNASE3	-5.5003	-6.4962	-5.6372	0.539820868	-0.36105
AKT3	1.1501	1.4731	1.6737	0.264173655	-0.3621
EVPL	-0.3031	-0.7747	-0.1766	0.315206921	-0.3623
CHGA	2.8269	2.8668	3.2093	0.210209427	-0.36245
SUSD4	-2.4885	-2.9582	-2.359	0.315285315	-0.36435
CABP2	-1.2408	-1.2949	-0.9033	0.212204155	-0.36455
CD226	-2.4539	-2.1193	-1.922	0.268887232	-0.3646
OTOA	-0.3739	-0.0645	0.1457	0.261373475	-0.3649
RBP7	-6.1941	-8.1751	-6.8192	1.012717122	-0.3654
DDX4	-0.4742	0.0952	0.1759	0.354344197	-0.3654
CTF1	-0.7599	-1.224	-0.6256	0.313981916	-0.36635
ACTA2	1.7346	1.7164	2.0921	0.211852142	-0.3666

SPTBN2	-0.0902	-0.5015	0.071	0.295214436	-0.36685
MMP1	-6.214	-5.95	-5.7137	0.250277772	-0.3683
KCNC4	-0.2566	-0.3511	0.0651	0.218190887	-0.36895
CYP24A1	-1.3177	-1.4912	-1.0353	0.230107518	-0.36915
CRX	-0.2062	-0.3972	0.0678	0.233731327	-0.3695
CORO6	0.0741	0.0396	0.428	0.214976751	-0.37115
MAPT	0.3752	-0.3105	0.404	0.404459347	-0.37165
MRPL28	-0.2551	-0.1376	0.176	0.222859605	-0.37235
TPSD1	4.4927	4.4248	4.8317	0.217982805	-0.37295
BRME1	-0.6503	-0.1296	-0.017	0.337855033	-0.37295
CGREF1	-3.4021	-3.9079	-3.282	0.33216674	-0.373
MYOM2	-2.0485	-2.9101	-2.1063	0.481627422	-0.373
VSIG10	-1.4707	-1.4016	-1.0629	0.218248078	-0.37325
GATA3	0.2429	-0.2401	0.3748	0.323725352	-0.3734
CEACAM20	-0.2521	-0.2351	0.1302	0.215980856	-0.3738
SPINK8	-0.3581	-0.5338	-0.0703	0.233998426	-0.37565
FOS	-1.8781	-0.5326	-0.8286	0.707040369	-0.37675
BTNL10	-0.031	0.0872	0.4049	0.225430455	-0.3768
RASA1	0.9623	0.3526	1.0349	0.374730601	-0.37745
LRTM2	-2.2268	-2.3386	-1.9051	0.225060133	-0.3776
HEPACAM2	-3.9313	-3.9642	-3.5683	0.219692292	-0.37945
FCGR2A	1.167	1.307	1.6177	0.230674728	-0.3807
FN1	-1.0991	-0.9828	-0.6593	0.227889586	-0.38165
PENK	-2.7692	-2.64	-2.3226	0.229814041	-0.382
EPHA4	-6.8833	-6.9888	-6.5528	0.227470328	-0.38325
ITIH1	1.3942	1.3959	1.7786	0.221444327	-0.38355
DNAJB8	-0.2244	-0.4715	0.0357	0.253627765	-0.38365
PIGR	-3.326	-3.2272	-2.8929	0.226970461	-0.3837
TNFSF13B	-3.8128	-3.9119	-3.4785	0.22708767	-0.38385
CDKL5	0.363	0.0462	0.5899	0.273085927	-0.3853
TFF2	-7.3615	-7.6494	-7.1192	0.265426619	-0.38625
IL32	-5.3144	-5.6704	-5.1056	0.285579084	-0.3868
IGFBP4	-3.68	-3.8692	-3.3871	0.242901715	-0.3875
CDAN1	0.5613	0.4804	0.9085	0.2274358	-0.38765
RANGAP1	-1.0348	-0.9261	-0.5923	0.23059502	-0.38815
BAMBI	0.062	0.0943	0.4667	0.224910034	-0.38855
GLI2	0.903	0.8549	1.2677	0.225729757	-0.38875
PTEN	-0.3069	-1.1975	-0.3616	0.499147483	-0.3906
GRN	0.496	0.6432	0.9605	0.23738414	-0.3909
CEBPA	0.1297	-0.0725	0.4242	0.249775226	-0.3956
CHL1	-4.6826	-4.8561	-4.3737	0.244346482	-0.39565
ALDH1A1	-3.0412	-3.3287	-2.7893	0.269895727	-0.39565
LRG1	-0.8168	-0.8376	-0.4315	0.228694097	-0.3957
CSF2RA	-2.643	-2.5012	-2.1759	0.23948199	-0.3962
BRD3	0.4201	0.1745	0.6945	0.260132889	-0.3972
APCS	-5.3722	-4.8751	-4.7262	0.338278884	-0.39745
CTSS	-0.5283	-0.2555	0.0058	0.267070634	-0.3977
MEP1B	-3.4476	-3.8847	-3.2683	0.317057792	-0.39785
LYZL2	-0.3124	-0.6606	-0.0881	0.288475874	-0.3984
LCAT	-3.8075	-4.1415	-3.5738	0.285322911	-0.4007

RRP15	-0.3858	0.5846	0.5011	0.537779388	-0.4017
PSMC3	0.0888	0.0223	0.4573	0.234321538	-0.40175
MAP1LC3A	0.9302	0.516	1.125	0.311017063	-0.4019
ITGB6	-2.9225	-3.8173	-2.9678	0.5040452	-0.4021
CPA2	-7.6629	-8.2672	-7.5617	0.381477431	-0.40335
RASGRF1	0.2081	0.3947	0.705	0.251003074	-0.4036
AGR2	-1.846	-2.4269	-1.7318	0.372748901	-0.40465
SEMA6C	-0.3826	-0.902	-0.2375	0.349378167	-0.4048
MEPE	-9.386	-10.5533	-9.5628	0.629144628	-0.40685
SPESP1	0.0017	-0.3448	0.2361	0.292247161	-0.40765
TGFBR3	-4.4469	-4.6305	-4.1302	0.253083629	-0.4085
O1-OID3023	-5.9033	-6.1877	-5.6368	0.275498463	-0.4087
LY96	-3.8076	-3.2843	-3.1367	0.352546796	-0.40925
PAFAH1B3	0.1137	0.2726	0.603	0.249608981	-0.40985
SH3GL3	-0.9742	-1.3163	-0.7346	0.292351233	-0.41065
ITGAL	-4.3156	-4.4819	-3.9859	0.252445961	-0.41285
FETUB	-8.1528	-7.4625	-7.3947	0.419489082	-0.41295
SAG	0.2453	-0.024	0.5236	0.273812326	-0.41295
LILRA3	-4.8378	-5.6382	-4.824	0.466145943	-0.414
MYOM1	0.0369	-1.1501	-0.1426	0.639823609	-0.414
CEACAM21	-2.7062	-3.315	-2.5965	0.387064547	-0.4141
APOH	-1.3891	-1.7939	-1.1764	0.313690553	-0.4151
KLK13	-4.4245	-4.7378	-4.1657	0.286482326	-0.41545
MAPRE3	-0.5499	-0.545	-0.1301	0.24096959	-0.41735
DUSP29	-0.541	-0.8009	-0.253	0.27407007	-0.41795
ISLR2	-2.7002	-3.9919	-2.9275	0.689577351	-0.41855
FABP9	-1.9526	-1.9673	-1.541	0.241992541	-0.41895
SLC39A5	-3.2393	-3.2466	-2.8237	0.242081618	-0.41925
ANGPT2	-7.2928	-6.8971	-6.675	0.312938689	-0.41995
CLEC4C	-4.5706	-4.82	-4.2749	0.272877524	-0.4204
GALNT5	-1.3922	-1.6531	-1.1018	0.275781514	-0.42085
HS6ST2	-3.5595	-3.756	-3.2361	0.262518514	-0.42165
CLEC12A	-0.656	-0.6884	-0.2485	0.245159139	-0.4237
ACVRL1	-5.8202	-6.4906	-5.7309	0.415241845	-0.4245
PGLYRP4	-1.1843	-1.847	-1.0898	0.412604205	-0.42585
OLR1	-4.6292	-4.7626	-4.2694	0.255113648	-0.4265
LAYN	-5.0678	-5.2248	-4.7164	0.260320802	-0.4299
CD8A	-6.7765	-6.8739	-6.3945	0.25338874	-0.4307
CA12	-8.9232	-5.2069	-6.6325	1.874856774	-0.43255
CXCL9	-4.3418	-4.3121	-3.8941	0.250346886	-0.43285
SIGLEC6	1.7752	1.8055	2.2237	0.250653007	-0.43335
CXCL16	0.3292	0.3722	0.7854	0.25189339	-0.4347
TJP3	-0.1093	-0.5385	0.1115	0.330521124	-0.4354
SERPINB5	-0.1473	-1.2161	-0.2424	0.591533197	-0.4393
DPP6	-3.8102	-3.7643	-3.3479	0.254694935	-0.43935
ECI2	-0.727	-0.2655	-0.0563	0.343167933	-0.43995
FCAR	-4.0968	-4.6473	-3.9315	0.374776373	-0.44055
DEFB118	-0.4579	-0.5976	-0.0858	0.26454796	-0.44195
KIRREL1	-0.1446	-0.1436	0.3015	0.257267766	-0.4456
SIGLEC15	-0.1725	-0.3729	0.1786	0.279160533	-0.4513

BPIFA2	-4.9319	-5.2132	-4.6208	0.296324895	-0.45175
B3GNT7	-6.8684	-6.3198	-6.1417	0.37876432	-0.4524
CD300LF	-7.0053	-7.3673	-6.7282	0.320488487	-0.4581
LILRA2	-4.801	-4.5992	-4.2415	0.283346896	-0.4586
MSTN	-1.7051	-1.729	-1.2582	0.265186557	-0.45885
CCL11	-8.3017	-7.5272	-7.4549	0.469423011	-0.45955
PAIP2B	0.7092	1.3226	1.4767	0.406009569	-0.4608
SLA2	-1.4725	-1.2126	-0.8805	0.296732882	-0.46205
H2AP	-1.1934	-1.5258	-0.8969	0.314620729	-0.4627
ACAN	-4.1183	-4.6692	-3.9298	0.384216827	-0.46395
CEMIP2	-2.2739	-2.4415	-1.8918	0.281738726	-0.4659
DMP1	-7.2172	-6.8008	-6.5421	0.340605994	-0.4669
C1R	-0.6233	-0.65	-0.1695	0.270039374	-0.46715
PARD3	-2.8723	-2.6994	-2.3176	0.283830272	-0.46825
DAND5	2.1349	1.8127	2.4429	0.315126662	-0.4691
FGF20	-0.6206	-0.8986	-0.2876	0.305912297	-0.472
IGDCC3	-0.0232	0.1528	0.5368	0.286365734	-0.472
TPP1	-2.151	-2.0289	-1.6172	0.279686652	-0.47275
APOA4	-3.5897	-3.1236	-2.8832	0.359208301	-0.47345
ERCC1	0.4183	-0.4439	0.4633	0.511277107	-0.4761
PDGFC	-2.0933	-2.1598	-1.6502	0.277023471	-0.47635
BCL7A	-0.6378	-0.2517	0.0334	0.336864132	-0.47815
FH	-0.7711	0.2596	0.2235	0.584932312	-0.47925
LAIR2	-3.4581	-4.18	-3.3393	0.454977937	-0.47975
GPC1	-5.2849	-5.2525	-4.7873	0.278408142	-0.4814
TNFRSF11B	-6.2623	-6.2076	-5.7531	0.27953741	-0.48185
PITHD1	-0.8595	-1.7162	-0.8047	0.511170255	-0.48315
TNFRSF21	-6.6252	-7.2526	-6.4557	0.419803649	-0.4832
CLU	0.5539	0.6693	1.0951	0.285049773	-0.4835
MIA	-7.0536	-7.9729	-7.0297	0.537790222	-0.48355
LAMP3	-8.941	-9.7021	-8.8377	0.472075464	-0.48385
DAPK2	-1.5284	-2.2434	-1.4017	0.453823898	-0.4842
ATP6AP2	-2.3827	-2.4396	-1.925	0.282117015	-0.48615
CR2	-3.477	-3.3951	-2.9486	0.284393044	-0.48745
EIF2AK2	-3.6952	-4.0726	-3.3955	0.339292219	-0.4884
GPR101	-0.3781	-0.3206	0.1397	0.283813078	-0.48905
ATP1B2	0.0598	0.1572	0.5977	0.286607577	-0.4892
QSOX1	6.6255	6.7014	7.1548	0.286208217	-0.49135
CPE	-9.0844	-7.0732	-7.5861	1.045059387	-0.4927
NUBP1	-1.4923	-0.1504	-0.3276	0.728997204	-0.49375
CYB5A	-2.1076	-0.1504	-0.6338	1.019509183	-0.4952
ANKRA2	0.5333	-0.354	0.5851	0.527872039	-0.49545
HTR1A	-0.4086	-0.2373	0.1776	0.3014178	-0.50055
PDXDC1	0.0192	-0.3694	0.3261	0.348548854	-0.5012
CCL26	-2.6867	-2.0344	-1.8589	0.436185813	-0.50165
NAGA	-1.6722	-1.138	-0.9027	0.39430656	-0.5024
CNTN2	-8.1417	-8.5729	-7.8536	0.362014627	-0.5037
DDX53	-0.0357	-0.6966	0.14	0.441127128	-0.50615
PBLD	-1.1989	-0.9667	-0.5762	0.314685658	-0.5066
TNXB	-5.7719	-6.0346	-5.3963	0.320809793	-0.50695

MYOC	-4.2956	-4.9981	-4.139	0.457544646	-0.50785
PCDH17	-2.8911	-2.6737	-2.272	0.314088751	-0.5104
ADCYAP1R1	-3.4745	-2.7589	-2.6049	0.46404086	-0.5118
DGKZ	0.1315	0.0901	0.6231	0.296500006	-0.5123
CD84	-4.6204	-4.8079	-4.2018	0.310306145	-0.51235
DUOX2	-0.1819	0.279	0.5613	0.375159597	-0.51275
ATP1B4	0.5566	-0.1186	0.7339	0.449830816	-0.5149
PLA2G1B	-7.2963	-6.9135	-6.5898	0.353661745	-0.5151
FBLN2	5.0195	5.0209	5.5367	0.298202236	-0.5165
CDNF	-2.6749	-3.046	-2.3422	0.352074552	-0.51825
CD300C	-0.6106	-0.4106	0.0103	0.316931544	-0.5209
TERF1	0.6401	0.5787	1.1332	0.303970344	-0.5238
EHD3	-2.7714	-3.7622	-2.7425	0.580561214	-0.5243
PCSK9	-5.1436	-4.6102	-4.3518	0.403780749	-0.5251
CSF3R	-2.1789	-2.4597	-1.7937	0.334361003	-0.5256
MSMB	-4.0373	-3.261	-3.1233	0.49278105	-0.52585
TMOD4	-0.1781	0.7798	0.8272	0.567222364	-0.52635
STX16	-2.3175	-1.903	-1.5833	0.368118636	-0.52695
AIF1	-0.5273	-0.9184	-0.1938	0.362681362	-0.52905
LAMB1	-4.5871	-5.1682	-4.3467	0.42236075	-0.53095
FAM3C	-3.6566	-3.652	-3.1227	0.306928021	-0.5316
NXPE4	-0.3951	-0.5618	0.0537	0.318342211	-0.53215
PLAT	4.9607	5.0007	5.5136	0.30831932	-0.5329
TSLP	-0.1804	-0.4628	0.2113	0.338523623	-0.5329
TOP1MT	-0.5604	-0.152	0.1768	0.369315547	-0.533
GZMH	-4.4866	-4.5107	-3.9654	0.308107757	-0.53325
CSF1R	-7.4988	-6.3709	-6.4005	0.642818982	-0.53435
SLAMF8	-2.4673	-2.3405	-1.8679	0.315887469	-0.536
APOM	-7.4057	-6.8087	-6.5712	0.429962305	-0.536
EPHB6	-5.7766	-6.494	-5.5986	0.474005612	-0.5367
CFHR2	-3.294	-3.244	-2.7311	0.311561342	-0.5379
FKBP7	-0.4887	-1.5478	-0.4781	0.614554481	-0.54015
ANG	-5.1397	-4.8681	-4.4632	0.34043179	-0.5407
GP1BB	-0.7931	-1.5784	-0.6447	0.50174933	-0.54105
EFCAB14	-3.1756	-3.0569	-2.575	0.318076946	-0.54125
KYNU	-9.259	-8.3276	-8.2509	0.5611973	-0.5424
MINDY1	-1.6562	-2.1239	-1.3467	0.391274281	-0.54335
PROS1	0.5208	0.5664	1.089	0.315711197	-0.5454
CEBPB	0.101	-0.2516	0.4707	0.361183734	-0.546
FABP4	-5.918	-5.5512	-5.1878	0.365101319	-0.5468
CDON	-6.3271	-6.066	-5.6474	0.342877835	-0.54915
APOF	-7.914	-8.9415	-7.8772	0.604130916	-0.55055
ARHGAP1	-0.9528	-0.2396	-0.0447	0.478066568	-0.5515
ECSCR	-1.2945	-1.5997	-0.8951	0.353347931	-0.552
VIT	-5.3191	-5.2636	-4.735	0.322405288	-0.55635
TPPP3	-0.3058	-0.9915	-0.0902	0.470639554	-0.55845
THPO	-5.7356	-5.8265	-5.2225	0.325666092	-0.55855
CLEC1A	-4.1319	-4.7064	-3.8602	0.432035026	-0.55895
PLB1	-3.0571	-3.4193	-2.6777	0.370833242	-0.5605
SMPDL3B	-0.8173	-1.4	-0.545	0.436789724	-0.56365

GLB1	-3.6973	-4.0136	-3.2915	0.361973236	-0.56395
IL1RL1	4.5292	4.601	5.1297	0.327942876	-0.5646
ELAVL4	0.5237	1.4186	1.536	0.553681626	-0.56485
SESTD1	0.2543	-1.0403	0.1784	0.726519059	-0.5714
CLEC14A	-3.5368	-4.3913	-3.3925	0.539844759	-0.57155
CD28	-3.1154	-3.1625	-2.5662	0.331514892	-0.57275
TDGF1	-3.3107	-4.6395	-3.4001	0.742721814	-0.575
KIRREL2	1.4111	1.0649	1.8133	0.374549027	-0.5753
B4GAT1	-4.9124	-4.5801	-4.1703	0.371723854	-0.57595
FURIN	-0.6882	-0.6519	-0.0919	0.334288144	-0.57815
DUSP3	-5.5468	-5.6357	-5.0125	0.337085039	-0.57875
PPM1B	-0.6379	-0.4694	0.0258	0.344991058	-0.57945
VSTM1	-3.4005	-4.0122	-3.1266	0.453410311	-0.57975
CXCL6	-9.907	-8.2507	-8.4959	0.893929149	-0.58295
WAS	-0.4536	-0.3288	0.1946	0.34392001	-0.5858
KHDC3L	-0.9211	-0.8894	-0.3193	0.33866949	-0.58595
CD177	-5.4192	-5.1409	-4.6917	0.367080323	-0.58835
BGN	-7.9429	-8.4567	-7.6094	0.426835249	-0.5904
TPM3	-0.1328	-0.3814	0.3383	0.365537335	-0.5954
PKN3	-2.3478	-1.5035	-1.3302	0.544424029	-0.59545
RAB3GAP1	-0.3387	-0.7984	0.0271	0.41363913	-0.59565
C4BPB	-6.2259	-5.7854	-5.409	0.408868932	-0.59665
REST	-0.295	-0.1492	0.3759	0.352867884	-0.598
PTX3	-7.654	-6.368	-6.4065	0.731611748	-0.6045
CDH4	-0.8407	-0.2093	0.0827	0.4719812	-0.6077
BOLA1	-1.2098	-1.1122	-0.552	0.354976675	-0.609
ST8SIA1	-0.5822	-0.4551	0.0928	0.358695279	-0.61145
TMED10	-0.2357	-1.1081	-0.0601	0.561281439	-0.6118
FCRL6	-5.3493	-5.5314	-4.8219	0.368488222	-0.61845
DO1-OID3056	-5.9293	-6.0737	-5.377	0.367713099	-0.6245
BLOC1S2	0.2216	0.2388	0.8587	0.362966541	-0.6285
ADAM23	-7.384	-7.2545	-6.6888	0.369704788	-0.63045
FOLR2	-8.8771	-9.9244	-8.7699	0.637860928	-0.63085
CCN4	-6.1232	-6.5222	-5.6899	0.416267778	-0.6328
VCAN	-3.7315	-4.0546	-3.2577	0.400817843	-0.63535
NOP56	-1.5348	-1.2333	-0.743	0.399633912	-0.64105
RBP5	-8.3768	-7.011	-7.0521	0.776952266	-0.6418
PCARE	-1.9226	-1.4895	-1.0626	0.430003725	-0.64345
PTK7	-0.8287	-2.2148	-0.8732	0.78773346	-0.64855
IL17D	-2.9363	-3.1162	-2.3761	0.385991377	-0.65015
HEBP1	-0.2693	0.2403	0.6386	0.455085607	-0.6531
VSTM2L	-1.0573	0.1953	0.2231	0.73134622	-0.6541
BMP6	-3.42	-3.9301	-3.0208	0.455775738	-0.65425
STXBP1	-0.2412	-0.8422	0.1131	0.482929874	-0.6548
TNNI3	7.5757	7.3087	8.0978	0.401363555	-0.6556
ST6GAL1	-0.4844	-0.3373	0.2495	0.388282942	-0.66035
CSF3	-3.8323	-2.811	-2.6598	0.637791944	-0.66185
B4GALT1	-4.5788	-4.1833	-3.7182	0.430768813	-0.66285
CELA3A	-7.0014	-7.1921	-6.4293	0.396973706	-0.66745
CASP4	-4.3018	-4.0994	-3.5331	0.398447224	-0.6675

SPINK5	-5.5851	-4.7829	-4.5093	0.559125424	-0.6747
GIPR	-1.1366	-0.1446	0.0349	0.630964407	-0.6755
CD4	-6.6853	-6.3748	-5.8535	0.42032828	-0.67655
CTSB	-2.0189	-1.9088	-1.2819	0.3975539	-0.68195
ICAM5	-1.9514	-1.6779	-1.1326	0.416850849	-0.68205
PTPRM	-5.2444	-5.9118	-4.8897	0.518961023	-0.6884
NUCB2	-3.0358	-2.7041	-2.1798	0.431596142	-0.69015
TNFRSF12A	-1.5263	-1.5313	-0.8386	0.398494998	-0.6902
GFRAL	-4.9381	-3.4937	-3.5244	0.825205181	-0.6915
KLRK1	-5.3953	-4.0135	-4.0074	0.799549338	-0.697
CANT1	-1.7639	-1.7252	-1.0453	0.404175634	-0.69925
MENT	-7.7915	-7.4067	-6.8991	0.447605958	-0.7
PRRT3	-5.8917	-6.3565	-5.4222	0.46715197	-0.7019
HBEGF	-7.9884	-7.2833	-6.9337	0.537242692	-0.70215
CEACAM1	-8.016	-10.4305	-8.5211	1.273495493	-0.70215
TNF-OID2007	1.5428	1.3619	2.1588	0.417777935	-0.70645
CDHR1	-2.1866	-2.1188	-1.4457	0.409591923	-0.707
MPRIP	-3.4773	-3.6945	-2.8779	0.422944394	-0.708
ART5	-0.6338	-0.2422	0.2701	0.453291124	-0.7081
UROS	-1.0608	-1.6361	-0.6384	0.500798885	-0.71005
MAGEA3	0.5337	0.2868	1.1223	0.429237661	-0.71205
CCL18	-4.1284	-4.2167	-3.4533	0.417599581	-0.71925
CDH15	-2.5273	-3.119	-2.1007	0.511375814	-0.72245
GPC5	-5.455	-5.3167	-4.6631	0.422970736	-0.72275
CCL23	-4.1872	-3.9798	-3.3607	0.430000353	-0.7228
PALLD	-0.3701	-0.4325	0.3218	0.418646203	-0.7231
LSP1	-4.1623	-4.3245	-3.5197	0.425626331	-0.7237
DNLZ	-3.4587	-3.2468	-2.6278	0.431751746	-0.72495
MERTK	-9.4666	-6.6094	-7.311	1.488984316	-0.727
RSPO1	-3.7559	-4.4045	-3.35	0.531884546	-0.7302
IOD1-OID302	-2.8816	-3.5141	-2.4676	0.527038028	-0.73025
AGRP	-6.3301	-6.3455	-5.6074	0.42176693	-0.7304
APLP1	-4.9849	-4.5948	-4.0516	0.468738225	-0.73825
ICA1	-1.7404	-1.877	-1.0703	0.431751947	-0.7384
CTSZ	2.2454	2.3501	3.0377	0.430405843	-0.73995
SNED1	-4.6165	-5.4063	-4.2664	0.583912959	-0.745
MTSS2	-1.0769	-1.6814	-0.6309	0.527239114	-0.74825
FGF7	-2.2822	-2.3558	-1.5707	0.433595668	-0.7483
IL5RA	-2.7337	-2.2058	-1.7179	0.508031242	-0.75185
IL31	-1.488	-0.9672	-0.474	0.5070626	-0.7536
B3_CGB5_CG	8.1797	8.1952	8.9412	0.435246769	-0.75375
VSIG10L	-1.4409	-0.9165	-0.4222	0.509424109	-0.7565
ENTPD5	-2.3087	-2.3021	-1.5464	0.43822128	-0.759
TNF-OID2123	2.1695	2.5682	3.1279	0.481448568	-0.75905
GP5	-6.5784	-6.6114	-5.8355	0.43875016	-0.7594
FKBP14	-1.2282	-0.3413	-0.0249	0.62378301	-0.75985
KAZALD1	-3.9805	-4.3551	-3.4051	0.478523827	-0.7627
PGA4	0.4678	0.401	1.1983	0.442300761	-0.7639
CXCL3	-5.7797	-5.5971	-4.9241	0.450614972	-0.7643
SPARC	-4.71	-2.9354	-3.0562	0.991535194	-0.7665

LRRC37A2	-4.6446	-5.0121	-4.0618	0.479197625	-0.76655
CBLN1	-0.3743	-0.7043	0.2274	0.472406566	-0.7667
INPP5J	-3.2246	-3.6066	-2.6442	0.484596382	-0.7714
CSNK1D	0.067	-0.3985	0.6061	0.502749146	-0.77185
TNFRSF13C	-3.0914	-3.6187	-2.5831	0.517829048	-0.77195
KEL	-3.6931	-3.3384	-2.7413	0.481016933	-0.77445
BEX3	-0.4878	-0.3768	0.3437	0.451448317	-0.776
GIMAP8	-1.5174	-2.0227	-0.994	0.514376538	-0.77605
AGRN	-1.0937	-0.9252	-0.2327	0.456302075	-0.77675
TET2	-1.23	-0.5076	-0.0909	0.576346172	-0.7779
CELSR2	-4.6495	-4.6422	-3.8629	0.452051129	-0.78295
IGDCC4	-5.1796	-4.8352	-4.2189	0.48672058	-0.7885
O1-OID3105	-5.2495	-5.0364	-4.3539	0.467852758	-0.78905
PLXDC1	-3.8945	-3.6119	-2.9638	0.477161548	-0.7894
SYTL4	0.1993	-0.1507	0.8138	0.48825719	-0.7895
CHGB	-4.9763	-5.3721	-4.3808	0.498991246	-0.7934
IL1R2	-10.2345	-9.8879	-9.2656	0.490943999	-0.7956
BRDT	0.2808	0.0657	0.9697	0.47224062	-0.79645
DIPK2B	-5.4056	-4.9717	-4.3844	0.512516657	-0.80425
KLRC1	-0.5749	-0.4176	0.3106	0.47242791	-0.80685
SEMA3G	-3.648	-3.4029	-2.7185	0.48174122	-0.80695
OSM	5.5663	5.779	6.4797	0.477933283	-0.80705
GDNF	-1.5246	-2.4582	-1.1832	0.660023727	-0.8082
DOCK9	-0.6619	-0.8108	0.0739	0.473685363	-0.81025
CLEC11A	4.8314	4.8997	5.6813	0.472209957	-0.81575
ADAM12	-2.404	-2.452	-1.612	0.471728736	-0.816
LECT2	-8.6247	-8.8259	-7.9083	0.482303528	-0.817
TRIM58	-1.3366	-1.7408	-0.7204	0.513857347	-0.8183
BLNK	-2.3548	-1.6221	-1.1654	0.600013408	-0.82305
CPVL	-7.9252	-9.0242	-7.6481	0.727809112	-0.8266
CCDC134	-0.863	-0.1913	0.3012	0.584394099	-0.82835
PF4	-3.6346	-1.8381	-1.9067	1.017984661	-0.82965
PGF	-2.108	-2.0685	-1.256	0.480905483	-0.83225
SIGLEC5	1.2923	1.2786	2.1195	0.481587711	-0.83405
RELT	3.3403	3.3832	4.1971	0.48276631	-0.83535
TNF-OID2047	2.9307	3.3163	3.9594	0.519693605	-0.8359
ADIPOQ	-3.6287	-3.1988	-2.5767	0.528918144	-0.83705
PRG3	1.605	1.585	2.4329	0.483865136	-0.8379
TSPAN15	0.6404	-0.0998	1.1175	0.613370435	-0.8472
TIMP3	1.9146	2.0943	2.853	0.49808171	-0.84855
CACNB1	0.0008	-0.1001	0.8011	0.493764846	-0.85075
RNF149	-4.0598	-3.4309	-2.8944	0.583310183	-0.85095
KLRF1	-2.6287	-3.196	-2.0588	0.568600495	-0.85355
ECM1	2.9047	4.1694	4.3942	0.802974821	-0.85715
MARCO	-5.0533	-5.3429	-4.3378	0.517370663	-0.8603
GRIN2B	-0.98	-1.2797	-0.2611	0.523479267	-0.86875
ADGRF5	-4.8024	-3.6966	-3.3768	0.748042004	-0.8727
PSAPL1	-5.8653	-5.1853	-4.6523	0.60798273	-0.873
PCOLCE	-0.4989	-0.2607	0.494	0.518356656	-0.8738
GM2A	4.6323	4.6183	5.5011	0.505691817	-0.8758

SELL	1.8178	2.3021	2.9449	0.565404389	-0.88495
PAPPA	-5.0711	-3.5763	-3.4385	0.90542795	-0.8852
TMPRSS11B	-0.3123	-0.9841	0.2391	0.612586788	-0.8873
FRMD4B	0.3767	-0.6072	0.7785	0.712936058	-0.89375
ADM	-8.4621	-8.7309	-7.6965	0.536715344	-0.9
SHC1	-1.2824	-1.4699	-0.4736	0.529453709	-0.90255
PTP4A3	-3.1607	-2.5874	-1.9641	0.598474079	-0.90995
KIR2DS4	-5.476	-4.5969	-4.121	0.687425465	-0.91545
DKK4	-6.9094	-7.0294	-6.0523	0.532876599	-0.9171
ADAM9	-3.2797	-3.2761	-2.3593	0.530357012	-0.9186
VMO1	-2.6496	-4.9627	-2.8803	1.274103859	-0.92585
EBI3_IL27	-9.1458	-7.213	-7.2429	1.107372134	-0.9365
PAM	-3.2932	-3.1666	-2.2913	0.545585505	-0.9386
TNF-OID2084	2.8231	2.7009	3.7023	0.546309961	-0.9403
LIF	7.8162	7.6964	8.7077	0.552547431	-0.9514
MYH7B	-0.74	-0.6589	0.2536	0.551735809	-0.95305
CSH1	0.3734	0.1286	1.2051	0.564284825	-0.9541
CA5A	-3.0902	-2.8652	-2.0201	0.56420047	-0.9576
MYBPC1	-3.2712	-3.3741	-2.3612	0.557472693	-0.96145
TPSG1	0.1443	0.3123	1.1928	0.563154286	-0.9645
GLA	-0.8769	-0.6586	0.202	0.570425639	-0.96975
DLL1	-4.1705	-4.1889	-3.1992	0.566166692	-0.9805
FST	3.6049	3.6918	4.6324	0.569800582	-0.98405
PODXL2	-0.6527	-0.7466	0.2927	0.574854019	-0.99235
GGA1	-1.2368	-1.5802	-0.4138	0.599408253	-0.9947
BOC	-5.6	-4.5628	-4.084	0.774950459	-0.9974
TCL1B	0.4592	-0.22	1.1173	0.668677743	-0.9977
SPINK4	-0.4755	-0.5187	0.5033	0.577984959	-1.0004
CD160	-7.3316	-8.3987	-6.8433	0.795446254	-1.02185
SHISA5	3.738	3.711	4.7465	0.59020639	-1.022
VEGFA	-1.7283	-1.6003	-0.632	0.599425083	-1.0323
ADAMTSL5	-2.4684	-3.0541	-1.71	0.673896612	-1.05125
SPP1	-4.0135	-3.8571	-2.8818	0.613244913	-1.0535
ADAMTS8	-3.1042	-3.1954	-2.0884	0.61449384	-1.0614
CTSV	-3.619	-4.1074	-2.7944	0.66363503	-1.0688
CHP1	-0.5653	0.2506	0.9121	0.740043447	-1.06945
IGFBP7	-0.4632	-0.4924	0.5919	0.617764132	-1.0697
IL10	-6.9437	-4.4778	-4.6362	1.380236068	-1.07455
MANEAL	-2.8207	-1.3116	-0.9916	0.976847789	-1.07455
HGF	-8.7509	-8.6253	-7.5999	0.6314034	-1.0882
WASF3	-1.8088	1.0905	0.732	1.580618412	-1.09115
GHRHR	-0.5857	-0.6542	0.4894	0.641398631	-1.10935
CXCL12	-2.3883	-4.7147	-2.4324	1.330599806	-1.1191
FGFBP3	-2.9819	-2.9041	-1.8182	0.650567616	-1.1248
TREML2	-7.243	-6.9455	-5.9631	0.669795991	-1.13115
KIR2DL3	-3.6964	-3.9025	-2.6359	0.67963385	-1.16355
NTRK3	-6.5832	-6.3069	-5.2805	0.686399536	-1.16455
AIDA	-0.3064	-0.718	0.7053	0.732431378	-1.2175
HS3ST3B1	-3.1666	-2.8543	-1.791	0.721158043	-1.21945
GKN1	-1.972	-2.9386	-1.2241	0.85957162	-1.2312

SCARA5	-10.1203	-11.8945	-9.7757	1.136943874	-1.2317
ALS7_LGALS	-5.9446	-5.7026	-4.5898	0.722538451	-1.2338
GPIHBP1	-7.7766	-2.4587	-3.8587	2.756507477	-1.25895
CRISP2	-10.078	-7.6027	-7.5572	1.442429258	-1.28315
CASP9	-0.8659	-0.0963	0.8119	0.839853582	-1.293
IL15	-4.632	-5.2269	-3.6303	0.806891201	-1.29915
CRH	-5.3269	-5.6523	-4.1893	0.768156659	-1.3003
C2	-9.355	-12.9559	-9.8544	1.950862349	-1.30105
S100A12	-8.8994	-7.3071	-6.7607	1.1111563	-1.34255
MN1	0.4271	-1.1212	0.998	1.096522407	-1.34505
GALNT7	-4.5929	-6.8774	-4.3674	1.388637852	-1.36775
CD34	-5.1591	-6.5209	-4.4676	1.044725832	-1.3724
IGFBPL1	1.1815	1.2232	2.5827	0.79721814	-1.38035
CXCL11	-3.6833	-3.7837	-2.3455	0.802932982	-1.388
CEACAM5	-4.3257	-3.9651	-2.7359	0.833509552	-1.4095
MSLN	-6.7745	-6.0582	-5.0057	0.889709258	-1.41065
CCL8	-10.3711	-10.3029	-8.9197	0.81898875	-1.4173
GHRL	-6.8753	-6.5007	-5.2123	0.872340599	-1.4757
EGFL7	0.3173	0.6746	1.9798	0.875130141	-1.48385
KCNIP4	-0.1736	-2.0704	0.4018	1.293619486	-1.5238
AKR1C4	-1.9422	-1.3119	-0.0901	0.941660567	-1.53695
GALNT10	-3.9505	-6.9969	-3.9316	1.764321128	-1.5421
LEFTY2	-12.6067	-8.7056	-9.0192	2.167451746	-1.63695
PLXNA4	-7.7256	-4.8733	-4.6535	1.713754443	-1.64595
CXCL10	-5.2619	-5.0898	-3.5069	0.967403382	-1.66895
GPA33	-10.4304	-6.9943	-7.0402	1.970716708	-1.67215
PSPN	-6.0625	-4.3477	-3.5123	1.300125138	-1.6928
FCRL2	-12.1643	-8.6149	-8.6861	2.029005711	-1.7035
WIF1	-8.7443	-11.4726	-8.4006	1.683198076	-1.70785
TRIM40	-1.7566	-1.2124	0.2871	1.058407891	-1.7716
TNFRSF19	-4.358	-4.4658	-2.5174	1.095117327	-1.8945
PNLIPRP1	-11.2661	-5.8402	-6.6317	2.930998875	-1.92145
HS6ST1	-4.6672	-3.8917	-2.2734	1.221377199	-2.00605
CBLIF	-8.1986	-10.3476	-7.2538	1.585478187	-2.0193
GPR37	-7.1626	-5.9354	-4.4602	1.353095257	-2.0888
UPB1	-2.4965	-9.1222	-3.6298	3.543790982	-2.17955
SCN4B	-7.1043	-10.9731	-6.785	2.331299621	-2.2537
CD5	-9.4266	-9.2804	-7.0564	1.32824436	-2.2971
KIR3DL1	-9.6159	-6.4588	-5.6194	2.107284391	-2.41795
CD7	-5.6127	-4.8767	-2.7385	1.493018245	-2.5062
APOA2	-5.9514	-7.6163	-3.7812	1.923090051	-3.00265
CCL25	-12.3593	-9.7029	-7.9829	2.204833248	-3.0482
CLEC4A	-11.5762	-7.1834	-5.5926	3.099215101	-3.7872
MUC16	-8.1825	-4.6468	-1.9178	3.140994577	-4.49685
COL24A1	-7.8525	-7.994	-3.1045	2.783006064	-4.81875
