# Supplementary material for: BAG6 restricts pancreatic cancer progression by suppressing the release of IL33-presenting extracellular vesicles and the activation of mast cells
Source: Cell Mol Immunol. 2024 Jun 28;21(8):918–31. doi: 10.1038/s41423-024-01195-1 (PMC11291976; doi:10.1038/s41423-024-01195-1)
Supplement: Supplementary file 13 — Table S4 [file 41423_2024_1195_MOESM13_ESM.pdf]

Supplementary table S4: Antibodies used in this study.			
**Antibody name**	**Supplier**	**Cat Number**	**Clone**
mouse monoclonal anti-beta-Actin antibody	Sigma-Aldrich	A1978	AC-15
Anti BAG6 antibody (3E4) Mouse and Human	Self-made		
mouse monoclonal anti-CD4 antibody	Dako	M7310	4B12
mouse monoclonal anti-CD8 antibody	Dako	M7103	C8/144B
mouse monoclonal anti-CD56 antibody	Dako	M7304	123C3
mouse monoclonal anti-Alpha SMA	Progen	61001	1A4/ASM-1
polyclonal Goat-anti-mouse anti-GFP antibody	Rockland	600-101.215	polyclonal
monoclonal mouse anti-TSG101 antibody	Abcam	ab83	4A10
Mouse monoclonal Alix antibody	Santa Cruz	SC-53540	1A12
Purified Mouse Anti-Flotillin-1	BD Trans. Laboratories™	610821	18
monoclonal rabbit anti-Cd63 antibody	Abcam	ab217345	EPR21151
monoclonal mouse antu-Hsp70/72 antibody	Enzo Lifesciences	ADI-SPA-810F	C92F3A-5
Purified anti-mouse Cd9 Antibody	Biolegend	124802	MZ3
polyclonal rabbit anti-Histone H2A antibody	Cell Signaling	2578	polyclonal
Rabbit polyclonal anti-CD117 antibody	Dako	A4502	polyclonal
Rabbit anti-gapdh HRP (D16H11)	Cell signaling	8884	
Goat anti-rabbit IgG, HRP-conjugated	Cell Signaling	7074S	
Horse anti-mouse IgG, HRP-conjugated	Cell Signaling	7076S	
F(ab')2-Rabbit anti-Goat IgG (H+L), Alexa Fluor™ 48	Thermo Fisher	A-21222	polyclonal
Anti-IL-33 polyclonal antibody	Thermo Fisher	PA5-96929	polyclonal
Anti-PDGF antibody, neutralizing	Merck KGaA	06-127	polyclonal
Purified anti-human Ki-67 Antibody	Biolegend	398502	W17211A
Chicken anti-Rat IgG (H+L), Alexa Fluor™ 647	Thermo Fisher	A-21472	Polyclonal
Hoechst 33342 Lösung (Nucleus staining)	Thermo Fisher	62249	
Actin-Alexa Fluor™ 546 Phalloidin	Thermo Fisher	A22283	
ST2 Polyclonal Antibody	Thermo Fisher	PA5-20077	Polyclonal
Goat anti-Mouse IgG (H + L)	Neta Scientific (LI-COR)	IRDye® 680RD	Polyclonal
Rabbit Polyclonal Calnexin antibody	Abcam	ab22595	Polyclonal
Mouse IL-33 Antibody	R&D Systems	AF3626-SP	Polyclonal
**Antibody name (single-cell sequncing)**			
Anti-mouse Cd4 antibody	Becton Dickinson	940108	RM4-5
Anti-mouse Cd279 antibody	Becton Dickinson	940128	J43
Anti-mouse NK-1.1 antibody	Becton Dickinson	940121	PK136
Anti-mouse Cd69 antibody	Becton Dickinson	940126	H1.2F3
Anti-mouse Cd8b antibody	Becton Dickinson	940181	53-5.8
Anti-mouse Cd11b antibody	Becton Dickinson	940008	M1/70
Anti-mouse Cd335 antibody	Becton Dickinson	940140	29A1.4
Anti-mouse Cd274 antibody	Becton Dickinson	940142	MIH5
Anti-mouse Cd45R antibody	Becton Dickinson	940110	RA3-6B2
Anti-mouse Cd45 antibody	Becton Dickinson	940320	30-F11
