# Supplementary material for: BAG6 restricts pancreatic cancer progression by suppressing the release of IL33-presenting extracellular vesicles and the activation of mast cells
Source: Cell Mol Immunol. 2024 Jun 28;21(8):918–31. doi: 10.1038/s41423-024-01195-1 (PMC11291976; doi:10.1038/s41423-024-01195-1)
Supplement: Supplementary file 14 — Table S5 [file 41423_2024_1195_MOESM14_ESM.pdf]

Supplementary table S5: List of customized gene panel for single cell sequencing.					
Gene	Transcript_id	PolyA_location	avg_(%)	max_(%)	Prod.length
Abcc9	ENSMUST00000087527.10	chr6(-): 142589133	100	100	478
Ackr1	ENSMUST00000038227.5	chr1(-): 173331890	100	100	511
Acod1	ENSMUST00000022722.6	chr14(+): 103056573			344
Acsbg1	ENSMUST00000034822.11	chr9(-): 54604891	96,4	100	556
Acta2	ENSMUST00000039631.9	chr19(-): 34241525	85,2	100	353
Bcam	ENSMUST00000003061.13	chr7(-): 19756140	100	100	362
Bcat1	ENSMUST00000111742.7	chr6(-): 144999156	100	100	545
Car6	ENSMUST00000030817.4	chr4(-): 150187015			548
Cav1	ENSMUST00000007799.12	chr6(+): 17341297	99,5	100	531
Ccl7	ENSMUST00000021011.2	chr11(+): 82047520	100	100	516
Ccn3	ENSMUST00000050027.8	chr15(+): 54753533	53,3	100	399
Cd300lg	ENSMUST00000017453.11	chr11(+): 102055611	55,5	100	345
Cd300lg	ENSMUST00000017453.11	chr11(+): 102054686	43,6	86,6	521
Cdh10	ENSMUST00000040562.13	chr15(+): 19014236			447
Cdh11	ENSMUST00000075190.4	chr8(-): 102632101	50	100	387
Cdh11	ENSMUST00000075190.4	chr8(-): 102633509	49,7	92,1	578
Cdh18	ENSMUST00000164787.7	chr15(+): 23474418			379
Cdh3	ENSMUST00000080797.7	chr8(+): 106556123	75,2	100	392
Cldn5	ENSMUST00000043577.2	chr16(+): 18778257	100	100	583
Clec3b	ENSMUST00000026890.5	chr9(+): 123157428	100	100	481
Col12a1	ENSMUST00000071750.12	chr9(-): 79598992	47,5	79,3	525
Col14a1	ENSMUST00000110221.9	chr15(+): 55520203			486
Col1a1	ENSMUST00000001547.7	chr11(+): 94953035	86	100	418
Col1a2	ENSMUST00000031668.9	chr6(+): 4540973	71,1	100	583
Crabp1	ENSMUST00000034830.8	chr9(+): 54773108	100	100	348
Csf3	ENSMUST00000038886.2	chr11(+): 98703629			386
Cxadr	ENSMUST00000023572.14	chr16(+): 78340731	80,3	100	478
Cxcl12	ENSMUST00000112871.7	chr6(+): 117181364	98,5	100	570
Cxcl14	ENSMUST00000021970.10	chr13(-): 56288684	54,8	100	539
Cxcl14	ENSMUST00000021970.10	chr13(-): 56289702	44,9	100	431
Cxcl3	ENSMUST00000031326.9	chr5(+): 90788093			535
Cxcl5	ENSMUST00000031318.5	chr5(+): 90761623	99,3	100	499
Dcn	ENSMUST00000105287.10	chr10(+): 97518125	93,2	100	542
Dll4	ENSMUST00000102517.3	chr2(+): 119335640	97,9	100	482
Ednrb	ENSMUST00000022718.10	chr14(-): 103814620	79,5	100	482
Emcn	ENSMUST00000119475.5	chr3(+): 137431061	100	100	478
Ezr	ENSMUST00000064234.6	chr17(-): 6738612	48,7	100	394
Ezr	ENSMUST00000064234.6	chr17(-): 6738293	46,4	95,2	462
F11r	ENSMUST00000043839.4	chr1(+): 171464008	94,4	100	461
Fap	ENSMUST00000102732.9	chr2(-): 62500943			389
Flt4	ENSMUST00000020617.2	chr11(+): 49652726	97	100	472
Gas6	ENSMUST00000033828.6	chr8(-): 13465412	99,8	100	591
Gjb2	ENSMUST00000055698.7	chr14(-): 57098717	45,7	61,9	574
Gjb3	ENSMUST00000046532.3	chr4(-): 127325237	100	100	362
Gpm6a	ENSMUST00000033915.8	chr8(+): 55060840	59,1	75,8	533
Gpr84	ENSMUST00000079824.5	chr15(-): 103308235			379
H2-Q10	ENSMUST00000068291.6	chr17(+): 35474530	99,9	100	471
Has1	ENSMUST00000003762.7	chr17(-): 17843332	100	100	343

Hck	ENSMUST00000109799.7	chr2(+): 153151425	100	100	550
Hp	ENSMUST00000074898.7	chr8(-): 109575153	97,2	100	551
Igf1	ENSMUST00000105300.8	chr10(+): 87930893	87,3	100	490
Igfbp4	ENSMUST00000017637.12	chr11(+): 99052619	94,5	100	385
Igfbp7	ENSMUST00000163898.5	chr5(-): 77349266	99,7	100	404
Il11	ENSMUST00000094892.11	chr7(-): 4772373			569
Il13ra2	ENSMUST00000033646.8	chrX(-): 147383533	100	100	504
Irf5	ENSMUST00000004392.11	chr6(+): 29537318	99	100	399
Ism1	ENSMUST00000184404.7	chr2(+): 139758571	78,9	100	589
Itih2	ENSMUST00000042290.13	chr2(-): 10094646	100	100	444
Kcnj15	ENSMUST00000113854.7	chr16(+): 95299622	81,3	97,6	388
Klhdc8a	ENSMUST00000046071.4	chr1(+): 132305568	45,1	75	515
Lgals7	ENSMUST00000081457.4	chr7(+): 28866284	100	100	382
Lif	ENSMUST00000066283.11	chr11(+): 4272512	100	100	458
Lipg	ENSMUST00000066532.4	chr18(-): 74939368	100	100	488
Lrrn4	ENSMUST00000049787.2	chr2(-): 132868311	89,7	93,7	480
Lum	ENSMUST00000038160.5	chr10(+): 97572702	99,8	100	546
Ly6a	ENSMUST00000023248.12	chr15(-): 74994879	91,9	100	537
Ly6c1	ENSMUST00000065408.15	chr15(-): 75045040	100	100	373
Lyve1	ENSMUST00000033050.4	chr7(-): 110850648	99,5	100	513
Mmrn1	ENSMUST00000129603.3	chr6(+): 60989367	100	100	348
Mogat2	ENSMUST00000064231.7	chr7(-): 99219086	100	100	466
Msln	ENSMUST00000237359.1	chr17(-): 25748618	100	100	569
Ndufa4	ENSMUST00000204978.2	chr6(-): 11900388	99,2	100	352
Nfe2l3	ENSMUST00000005103.11	chr6(+): 51458767	100	100	401
Nid2	ENSMUST00000022340.4	chr14(+): 19811785	88,7	100	456
Nkain4	ENSMUST00000103053.9	chr2(-): 180934802	100	100	569
Nos2	ENSMUST00000018610.6	chr11(+): 78960220	88,9	100	431
Pdgfra	ENSMUST00000000476.14	chr5(+): 75198199	54,6	100	435
Pdgfrb	ENSMUST00000025522.10	chr18(+): 61085038	100	100	554
Pdpn	ENSMUST00000030317.13	chr4(-): 143267437	100	100	442
Pecam1	ENSMUST00000080853.10	chr11(-): 106654220	81,7	100	566
Pi16	ENSMUST00000114701.9	chr17(+): 29328895	97,4	100	568
Piezo2	ENSMUST00000047480.12	chr18(-): 63010233	71,3	100	580
Pkp1	ENSMUST00000027667.12	chr1(-): 135871402	100	100	378
Pnpla3	ENSMUST00000045289.5	chr15(+): 84186206			435
Ptn	ENSMUST00000101534.4	chr6(-): 36714934	100	100	370
Ramp3	ENSMUST00000045374.7	chr11(+): 6677472	100	100	447
Rrm2	ENSMUST00000020980.11	chr12(+): 24714085	69,9	100	431
Saa1	ENSMUST00000128088.3	chr7(-): 46740527	100	100	364
Saa3	ENSMUST00000006956.8	chr7(-): 46712000	100	100	411
Saa4	ENSMUST00000006952.8	chr7(-): 46728017	67,1	92,4	448
Selp	ENSMUST00000162746.1	chr1(+): 164150026			433
Shank2	ENSMUST00000105902.7	chr7(+): 144424489	19,8	85,7	467
Shank2	ENSMUST00000105902.7	chr7(+): 144424036	73,4	100	537
Slpi	ENSMUST00000109367.9	chr2(-): 164354074	98,1	100	442
Smim10l2	ENSMUST00000068106.4	chrX(+): 56377796	100	100	420
Spink5	ENSMUST00000069245.7	chr18(+): 44022148	100	100	378
Steap4	ENSMUST00000115421.2	chr5(+): 7982194	96,9	100	562
Tagln	ENSMUST00000034590.3	chr9(-): 45930003	98,9	100	353

Thbs2	ENSMUST00000170872.2	chr17(-): 14665498	42,1	100	475
Thbs2	ENSMUST00000170872.2	chr17(-): 14667118	57,9	100	563
Tll1	ENSMUST00000066166.5	chr8(-): 64014931			430
Tmem252	ENSMUST00000057243.5	chr19(+): 24678247	86,3	95,1	479
Tnnt2	ENSMUST00000179863.7	chr1(+): 135852224	98,7	100	368
Vcam1	ENSMUST00000029574.12	chr3(-): 116110141	98,7	100	390
Vim	ENSMUST00000028062.7	chr2(+): 13582778	99,4	100	529
Vwf	ENSMUST00000112254.7	chr6(+): 125686672	99,9	100	496
Myl9	ENSMUST00000088552.6	chr2(+): 156781656	100	100	539
Myod1	ENSMUST00000072514.2	chr7(+): 46379096	100	100	451
