# Supplementary material for: BAG6 restricts pancreatic cancer progression by suppressing the release of IL33-presenting extracellular vesicles and the activation of mast cells
Source: Cell Mol Immunol. 2024 Jun 28;21(8):918–31. doi: 10.1038/s41423-024-01195-1 (PMC11291976; doi:10.1038/s41423-024-01195-1)
Supplement: Supplementary file 15 — Table S6 [file 41423_2024_1195_MOESM15_ESM.pdf]

Supplementary table S6: Antibodies used for flow cytometry and nano-FCM in this study.			
**Flow cytometry**	**Supplier**	**Cat. Number**	**Clone**
monoclonal hamster anti-Cd81 Antibody	BioLegend	104905	Eat-2
monoclonal hamster IgG Isotype Ctrl, FITC-conjugated	BioLegend	400905	HTK888
monoclonal rat anti-Il-33Rα (IL1RL1, ST2), PE-conjugated	BioLegend	145303	DIH9
monoclonal rat IgG2a, κ Isotype Ctrl, PE-conjugated	BioLegend	400507	RTK2758
monoclonal rat anti-Mouse IL-33, Alexa Fluor® 647-conjugat	R&D systems	IC3626R	396118
monoclonal rat IgG2a, κ Isotype Ctrl, APC-conjugated	BioLegend	400511	RTK2758
monoclonal rat anti-mouse CD117 (c-Kit), APC-conjugated	BioLegend	105811	2B8
monoclonal rat IgG2b, κ Isotype Ctrl, APC-conjugated	BioLegend	400611	RTK4530
**nano-flow cytometry**			
monoclonal rat anti-Cd63 antibody, PE-conjugated	BioLegend	143904	NVG-2
monoclonal hamster anti-Cd81, FITC-conjugated	Miltenyi Biotec	130-102-631	16-10A1
monoclonal rat anti-Cd9, FITC-conjugated	BioLegend	124808	MZ3
monoclonal hamster Isotype control, FITC-conjugated	BioLegend	400905	HTK888
monoclonal rat isotype control, FITC	BioLegend	400506	RTK2758
monoclonal rat isotype control, PE-conjugated	BioLegend	400508	RTK2758
monoclonal rat antiCd63, FITC-conjugated	BioLegend	143919	NVG-2
